# The Copper Chaperone ATOX1 Exhibits Differential Protein–Protein Interactions and Contributes to Skeletal Myoblast Differentiation

**DOI:** 10.1080/10985549.2026.2621941

**Published:** 2026-02-04

**Authors:** Nathan Ferguson, Yu Zhang, Alexandra M. Perez, Allison T. Mezzell, Jason D. Fivush, Vinit C. Shanbhag, Michael J. Petris, Katherine E. Vest

**Affiliations:** a Department of Molecular and Cellular Biosciences, University of Cincinnati, Cincinnati, Ohio, USA;; b Department of Biochemistry, University of Missouri, Columbia, Missouri, USA;; c Departments of Ophthalmology and Biochemistry, University of Missouri, Columbia, Missouri, USA

**Keywords:** Copper homeostasis, copper distribution, ATOX1, ATP7A, myoblast differentiation, metallochaperone

## Abstract

Copper is an essential but potentially toxic nutrient required for a variety of biological functions. Mammalian cells use a complex network of copper transporters and metallochaperones to maintain copper homeostasis. Previous work investigating the role of copper in various disease states has highlighted the importance of copper transporters and metallochaperones. However, questions remain about how copper distribution changes under dynamic conditions like tissue differentiation. We previously reported that the copper exporter ATP7A is required for skeletal myoblast differentiation and that its expression changes in a differentiation dependent manner. Here, we sought to further understand the ATP7A-mediated copper export pathway by examining ATOX1, the copper chaperone that delivers copper to ATP7A. To investigate the role of ATOX1 in a dynamic cellular context, we characterized its protein–protein interactions during myoblast differentiation using the proximity labeling protein APEX2 to biotinylate proteins near ATOX1. We discovered that the ATOX1 interactome undergoes dramatic changes as myoblasts differentiate. These dynamic interactions correlate with distinct phenotypes of ATOX1 deficiency in proliferating and differentiated cells. Together, our results highlight the dynamic interactome of ATOX1 and its contribution to myoblast differentiation.

## Introduction

Copper (Cu) is a vital trace nutrient required for a variety of biological processes. It serves as a catalytic cofactor in cytochrome c oxidase during cellular respiration, supports antioxidant defense via superoxide dismutase (SOD1), facilitates extracellular matrix remodeling through lysyl oxidases (LOX), and modulates cellular signaling by allosteric regulation of kinases such as the dual specificity mitogen-activated protein kinase kinases 1 and 2 (MEK1/2).^1^ However, unregulated copper levels can be toxic. Excess copper can induce oxidative stress, disrupt the function of other metalloproteins, or trigger cell death through a recently discovered pathway known as cuproptosis.^2,3^ To maintain copper homeostasis, cells rely on a network of transporters and chaperones that tightly control import, export, and intracellular distribution. In mammalian cells, the primary importer is CTR1, which facilitates the uptake of Cu^+1^ ions which are then transferred to various metallochaperones including ATOX1.^4^ The ATOX1 protein delivers copper to the primary exporter proteins, ATP7A and ATP7B, which exhibit tissue specific expression.^5^ ATP7A is expressed in most non-hepatic tissues and is primarily localized to the *trans*-Golgi network where it provides copper to secreted cuproproteins.^1^ Under high copper conditions, ATP7A translocates to the cell membrane to move intracellular copper directly to the extracellular space.^6,7^ Thus, in mammals, ATP7A is critically important for both copper mobilization and detoxification.

Disruptions in copper homeostasis can give rise to serious disease. Loss-of-function mutations in *ATP7A* lead to Menkes disease (MD), a fatal, infantile-onset copper deficiency characterized by hypotonia, connective tissue defects, neurodegeneration, and failure to thrive.^8^ The severity of Menkes disease symptoms and overall survival is known to vary, even between related individuals with the same mutations in *ATP7A*. Given the pleiotropic nature of symptoms arising from Menkes and other copper-related diseases, understanding dynamic cellular copper metabolism across multiple tissue types and differentiation states is of utmost importance. Research from various groups has shown that copper is important for early brain development, contributing to myelination, synaptic connectivity, and neurotransmitter synthesis.^9–11^ Consistent with the important role of copper in brain development, early supplementation in infants with Menkes disease has been shown to partially rescue developmental delays.^8^ We and others have shown that copper is important for differentiation of skeletal muscle, osteoblasts, and hematopoietic progenitor cells.^12–17^ Collectively, these studies highlight the essential and multifaceted roles copper plays across multiple cell and tissue types during differentiation and development. However, many questions remain surrounding the mechanism by which copper distribution is regulated within different cellular environments, particularly under shifting metabolic conditions.

To address these questions, our group uses skeletal muscle as a model system due to its well-characterized differentiation pathway. Skeletal muscle contains approximately 23% of total body copper and is notably affected in Menkes disease, where severe muscle weakness is a hallmark symptom.^1,8,18^ In previous work, we explored the molecular requirements for copper during skeletal muscle differentiation and found that the secreted copper dependent enzyme LOX plays a vital role in the differentiation of mononucleated, proliferating skeletal myoblasts to mature multinucleated, postmitotic myotubes.^12,13^ Notably, ATP7A is required for myotube formation as it provides copper to LOX, and thus deficiency in copper, ATP7A, or LOX activity impairs myotube formation.^12,19^ The present study focuses on the role of ATOX1 during skeletal myoblast differentiation. ATOX1 and its homologs were originally identified as antioxidant proteins, with many studies demonstrating that ATOX 1 expression increases in response to oxidative stress, mitigating cellular damage.^10,20^ More recently, ATOX1 has been shown to indirectly promote copper dependent activation of the MEK/ERK signaling cascade in human BRAF^V600E^ expressing cancer cells, while the copper chaperone for SOD1 (CCS) is responsible for direct copper delivery to MEK1/2.^21,22^ Other studies have implicated ATOX1 as a putative transcription factor that controls the expression of the cyclin-D pathway.^23,24^ These diverse roles highlight the functional importance of ATOX1 across multiple cell types. However, significant gaps remain in our understanding of how ATOX1 function varies across cellular contexts, particularly during dynamic processes such as differentiation.

In this study, we investigated the multifaceted role of ATOX1 during skeletal myoblast differentiation. To do so, we generated stable skeletal myoblast lines that express a construct containing the proximity labeling protein APEX2 fused to the amino (N)-terminus of ATOX1 (APEX2-ATOX1) under the control of an inducible promoter. This construct differs from the previously studied carboxy (C)-terminal tagged ATOX1 which contained both APEX2 and an exogenous nuclear localization signal (NLS).^25^ Using comparative proteomics across different stages of myoblast differentiation, we identified dynamic changes in APEX2-ATOX1 proximal proteins. In addition, we found that loss of ATOX1 leads to increased myoblast proliferation and reduced myoblast differentiation. These fundings suggest that ATOX1 functions are differentiation state-dependent in skeletal muscle cells and support the broader model that ATOX1 mediates diverse functions across mammalian tissues.

## Results

### APEX2-ATOX1 construct production and expression

**Figure 1.**
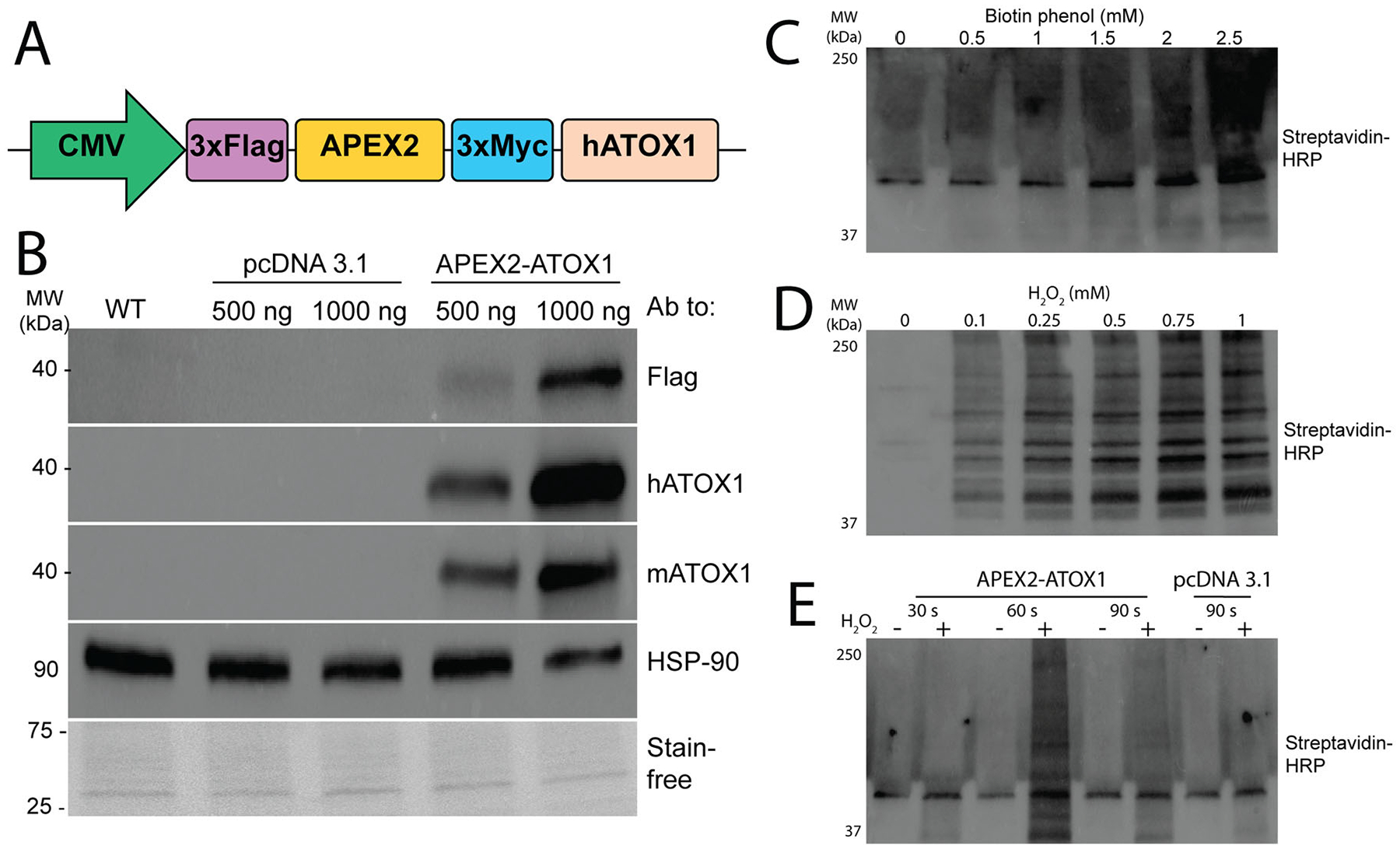
Generation of APEX2-ATOX1 constructs. (A) The APEX2-ATOX1 construct contains 3× Flag and 3× Myc tags flanking the full-length APEX2 sequence at the amino terminal end of the human ATOX1 protein. All tags are separated by glycine-serine hinges. Constructs were subcloned into a pcDNA 3.1 plasmid under the control of a CMV promoter. (B) Immunoblot of lysates from C2C12 myoblasts transfected with APEX2-ATOX1 constructs showing detection of APEX2-ATOX1 fusion constructs by antibodies to Flag, human (h) or mouse (m) ATOX1. Antibodies to HSP-90 and total proteins detected using stain-free gel imaging technology (Bio-Rad) were used as loading controls. Labeling efficiency was determined by probing blots with streptavidin-HRP in cells titrated with biotin phenol (C), hydrogen peroxide (H_2_O_2_) (D), or at variable time points in seconds (s) (E). For all, shown are representative images of three-to-five replicates of mixed populations of stable cells.

To elucidate the functional importance of ATOX1 in skeletal myoblast differentiation, we sought to identify its proximal protein binding partners using a proximity labeling approach. The APEX2 protein is an engineered ascorbate peroxidase that biotinylates proteins within approximately 20 nm of the bait protein.^26^ A construct encoding a DYKDDDDK (Flag) tag, APEX2, and a Myc tag at the amino (N) terminal end of human ATOX1 was expressed under the control of the cytomegalovirus immediate early gene promoter (CMV) in pcDNA 3.1 (Figure 1A). Expression of the construct was confirmed by immunoblot with antibodies targeting the Flag tag and was further validated using antibodies targeting human and mouse ATOX1, which detected a ~40 kDa band corresponding to the fusion protein, in contrast to the ~7 kDa endogenous mouse Atox1 (Figure 1B). Optimal labeling conditions were determined through titrations of biotin-phenol (BP) and hydrogen peroxide (H_2_O_2_) across various time points. Immunoblot analysis using streptavidin conjugated to horseradish peroxidase (HRP) to identify biotinylated proteins revealed that 2.5 mM BP, 0.75 mM H_2_O_2_, and 60 s were optimal for robust labeling (Figure 1C to E).

**Figure 2.**
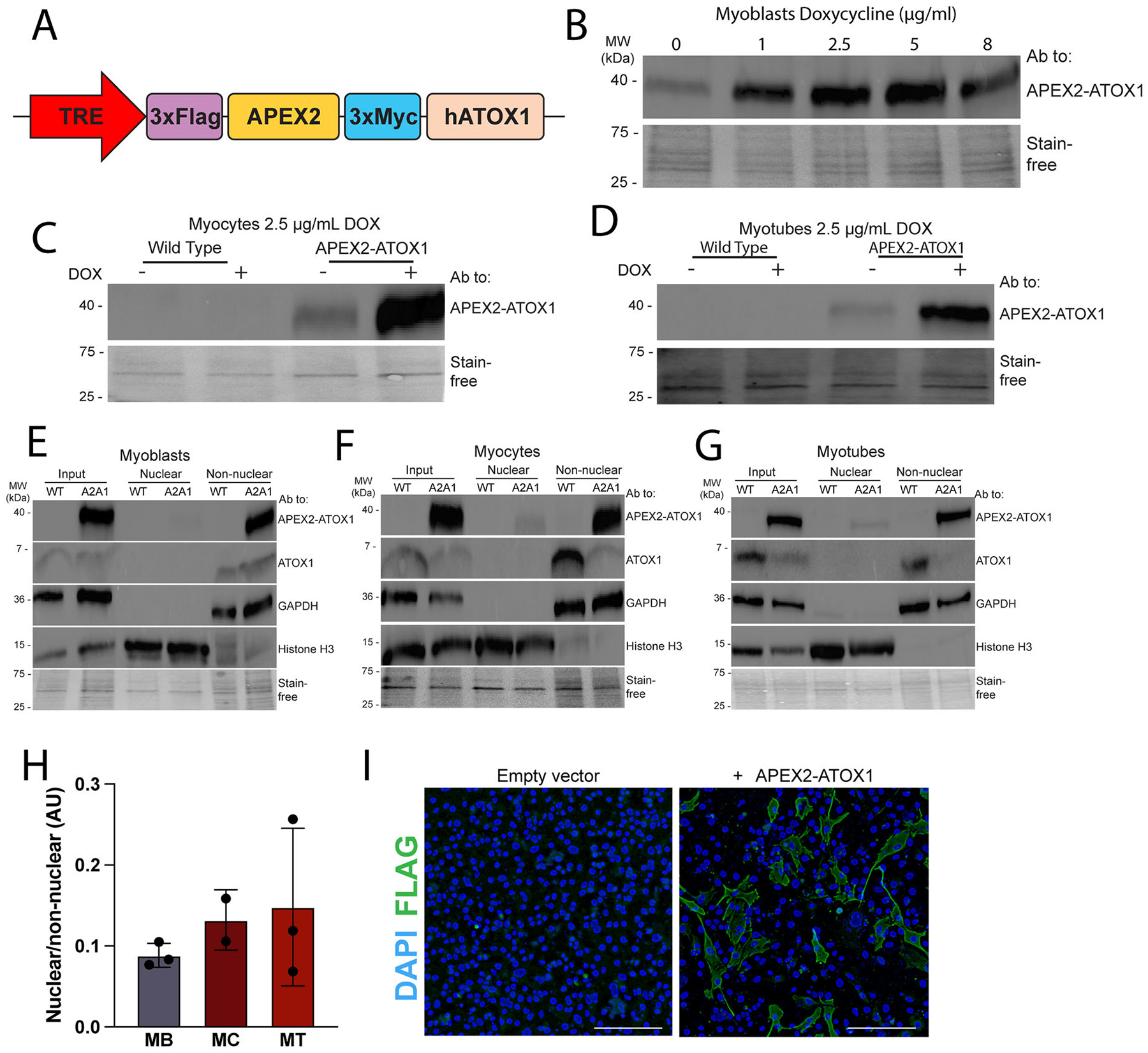
Stable cells express inducible APEX2-ATOX1. (A) Stable C2C12 cells were generated that express the APEX2-ATOX1 fusion construct under the control of a tetracycline responsive element (TRE). (B) Doxycycline (DOX) titration revealed the optimal concentration to be 2.5 μg/mL as determined by immunoblot using an antibody to ATOX1. DOX at 2.5 μg/mL was used to induce APEX2-ATOX1 expression in myocytes (C) and myotubes (D). Localization of APEX2-ATOX1 construct (A2A1) as determined by fractionation in myoblasts (E), myocytes (F), and myotubes (G) as analyzed by immunoblot using antibodies to *GAPDH* to indicate the non-nuclear fraction and histone H3 to indicate the nuclear fraction. Total protein as detected by Stain-free gel imaging technology (Bio-Rad) was used as a loading control. (H) Portion of APEX2-ATOX1 in the nuclear fraction as measured by densitometry. Shown for all is mean ± standard deviation for *n* = 2–3 experiments. Statistical significance was determined using one-way ANOVA with Dunnett’s post hoc correction for multiple comparisons. None of the comparisons were statistically significant. (I) Immunofluorescence staining using an antibody to the Flag tag in the APEX2-ATOX1 construct in transfected myoblasts showing its cytosolic localization in myoblasts. Myoblasts transfected with empty pcDNA 3.1 plasmid (empty vector) were used as a control and nuclei were visualized with DAPI. Images are representative of *n =* 3 replicates. Bar = 200 μm. For C to G, all immunoblots are representative of two-to-three replicates.

In order to generate sufficient material for streptavidin capture and comparative proteomics, the immortalized C2C12 myoblast line was used. C2C12 myoblasts can be maintained as proliferating myoblasts in normal growth medium or differentiated using low serum medium wherein they exit the cell cycle and flatten into myocytes, as defined by the molecular marker myogenin, then migrate and fuse to muscle-like myotubes as defined by multinucleated morphology and expression of embryonic myosin heavy chain (eMyHC).^27^ To eliminate variability from uneven transfection with pcDNA 3.1 plasmids, we generated stable C2C12 myoblast lines expressing APEX2-ATOX1 under the control of a doxycycline-inducible TRE promoter (Figure 2A). Cells were transduced and selected with puromycin to produce stable cells and doxycycline titration confirmed dose-dependent expression of APEX2-ATOX1 expression (Figure 2B). At all three stages of differentiation, a doxycycline concentration of 2.5 μg/mL was optimal for robust APEX2-ATOX1 expression (Figure 2B to D). Although N-terminal epitope tagging has been used to study ATOX1, we sought to confirm its localization as ATOX1 has previously been reported as a nuclear protein in some contexts.^23,28,29^ Here, after fractionation and immunoblot, we did not detect endogenous ATOX1 in the nucleus, though low abundance nuclear ATOX1 may fall below the threshold for detection by immunoblot (Figure 2E to G). Similarly, very little APEX2-ATOX1 was detected in the nuclear fraction in myoblasts, myocytes, and myotubes (Figure 2E to G). Quantification revealed that less than 25% of the APEX2-ATOX1 construct was detected in the nucleus (Figure 2H). Immunofluorescence staining using an antibody to the Flag tag on the APEX2-ATOX1 construct confirmed that it localizes primarily outside of the nucleus in myoblasts transfected with empty pcDNA 3.1 or pcDNA 3.1 encoding the APEX2-ATOX1 construct (Figure 2I). The diffuse staining pattern observed is suggestive of a primarily cytosolic localization of APEX2-ATOX1. These localization patterns align with previous observations in neuronal differentiation, where endogenous ATOX1 was not detected in nuclei throughout the differentiation process.^10^ Collectively, these results confirm that the APEX2-ATOX1 fusion construct mimics the localization of endogenous ATOX1.

### ATOX1 interacts with canonical and noncanonical binding partners in a differentiation dependent manner

**Figure 3.**
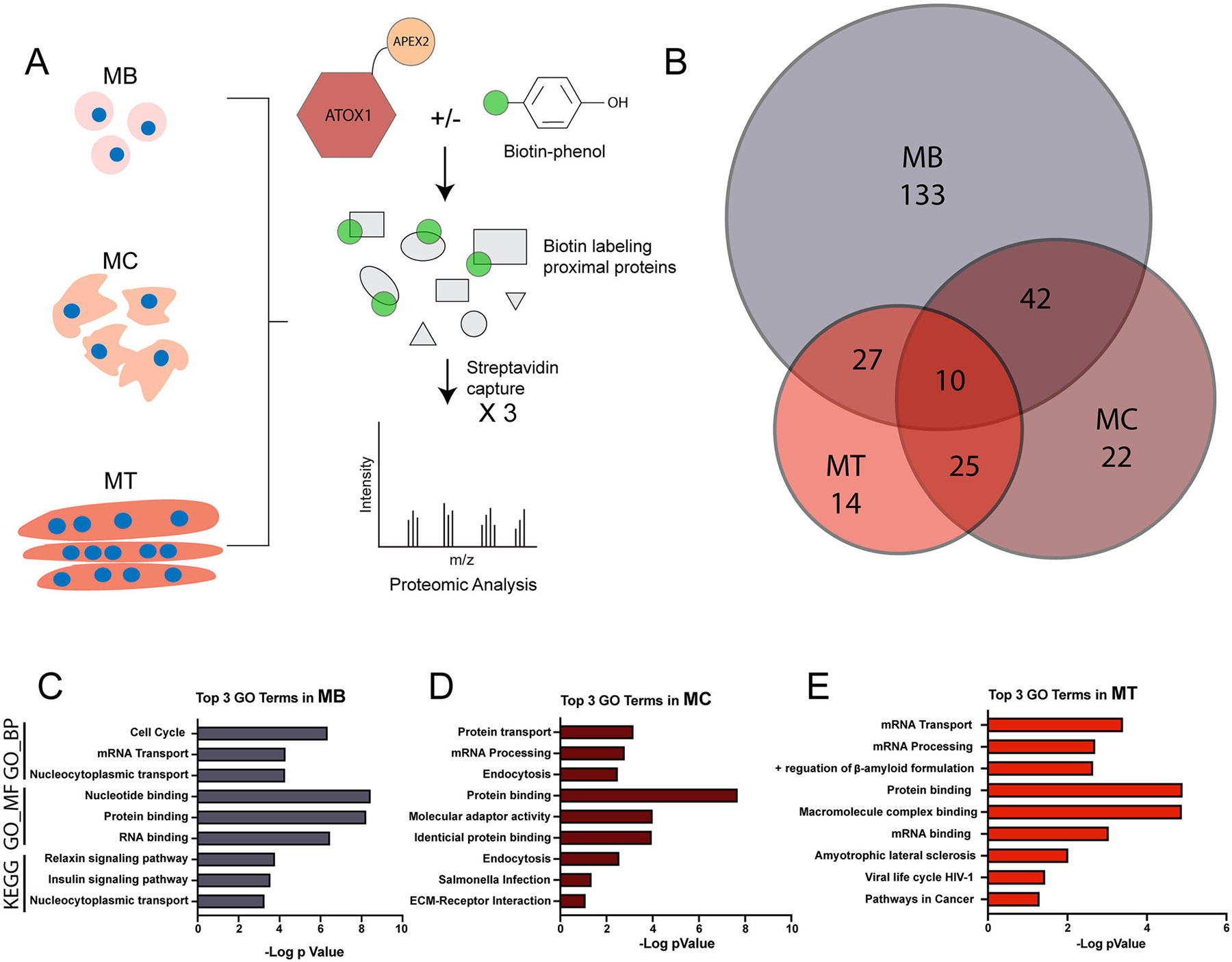
Comparative proteomic analysis and label-free quantification reveal differential ATOX1 proximal proteins detected during myoblast differentiation. (A) Expression of APEX2-ATOX1 was induced in myoblasts (MB), myocytes (MC), and myotubes (MT). After labeling, lysates were isolated with streptavidin beads and analyzed by comparative proteomics. Cells without biotin phenol were used as negative controls and three independent replicates per differentiation condition were used for proteomic analysis. (B) Venn diagram of proteins detected in all three replicates of MB, MC, and MT that were not detected in negative control cells without biotin phenol. These hits were used for gene ontology and pathway analysis. The top three hits each for GO_Biological Process (BP), GO_Molecular Function (MF), and the Kyoto Encyclopedia of Genes and Genome (KEGG) pathways are shown for MB (C), MC (D), and MT (E).

To characterize the ATOX1 interactome, we performed comparative proteomics with label-free quantification to identify proteins in proximity to APEX2-ATOX1. Construct expression was induced in myoblasts, myocytes, and myotubes, and biotinylated proteins were isolated from lysates by streptavidin-based bead capture in three independent experiments per differentiation state (Figure 3A). Induced cells without biotin phenol treatment were used as negative controls. Protein peaks were filtered to include only those with high confidence (false discovery rate of 99%) and proteins identified in -BP controls were filtered out yielding 708 total proteins. Proteins that were detected in all three replicates (and not detected in -BP controls) for each differentiation state were included for downstream functional annotation yielding 212, 99, and 76 ATOX1 proximal proteins in myoblasts, myocytes, and myotubes (Figure 3B). Summaries of proteomics data including key proteins detected can be found in Table 1, Supplementary Table 2, and Supplementary Table 3. Functional annotation of ATOX1 proximal proteins was performed using the NIH database for annotation, visualization, and integrated discovery (DAVID).^30^ Top GO terms for biological processes and molecular function and top KEGG pathways varied with differentiation state (Figure 3C to E). In myoblasts, ATOX1 proximal proteins are enriched in pathways related to cell cycle regulation and insulin signaling, reflecting a proliferative profile (Figure 3C). In myocytes, enrichment was observed in categories related to endomembrane trafficking and molecular adapter activity, which is consistent with the role of ATOX1 in delivering Cu to ATP7A (Figure 3D). In myotubes, ATOX1 proximal proteins are primarily enriched in functions related to mRNA binding, processing, and transport (Figure 3E). Comparable enrichment patters were also observed using the STRING database for pathway analysis (Supplemental Figure 1).^31^ Collectively, these findings suggest that the ATOX1 protein interaction network dynamically remodels in response to cellular differentiation state.

**Figure 4.**
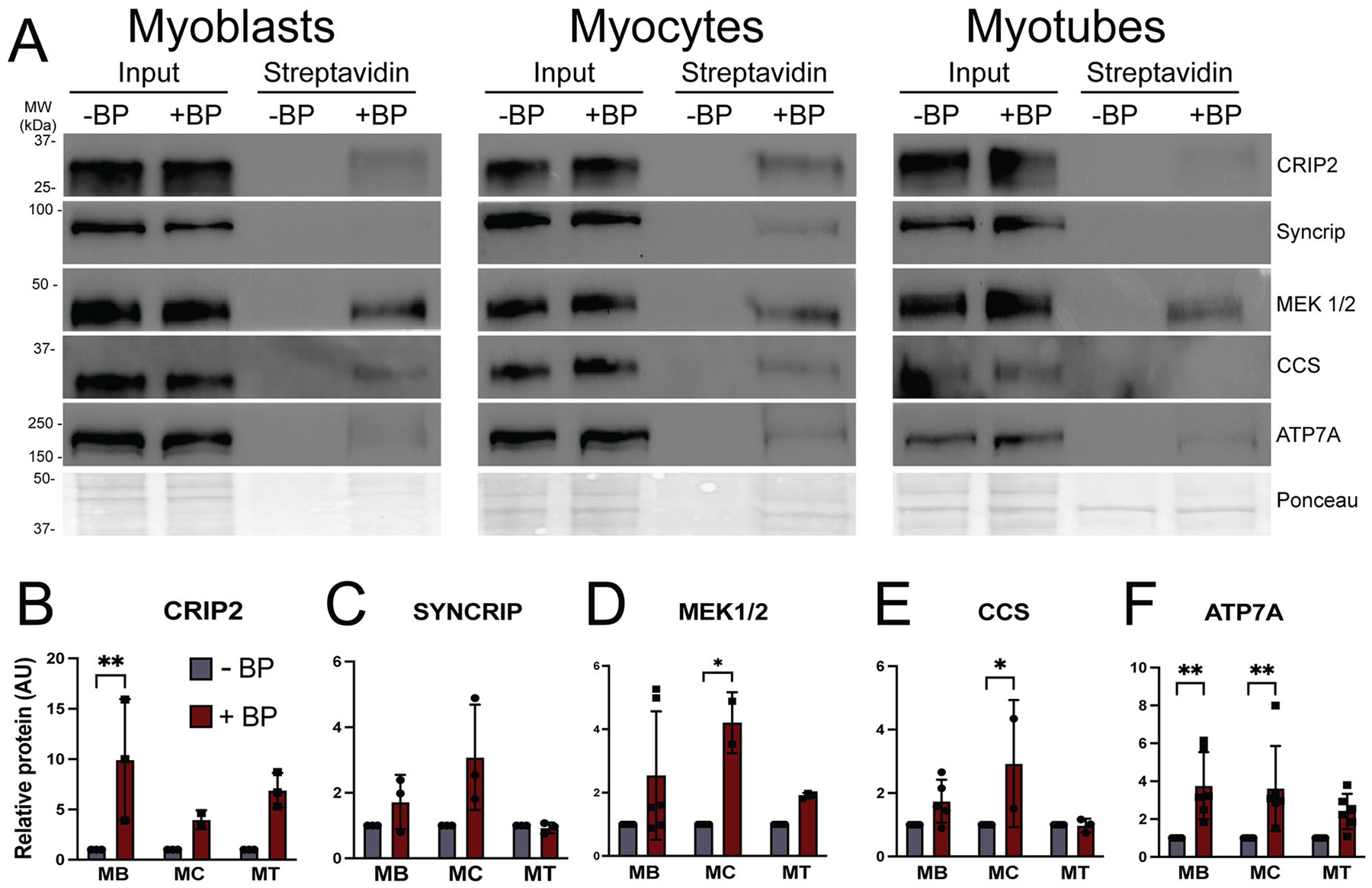
Shifting ATOX1 binding partners during myoblast differentiation detected by immunoblot. (A) Representative blots showing streptavidin bead elutions from myoblasts (MB), myocytes (MC), and myotubes (MT) expressing APEX2-ATOX1 and treated with biotin phenol (BP). Cells without BP (-BP) were used as negative controls. Total protein as detected by Ponceau stain was used as loading control. (B to F) Densitometric quantification of candidate ATOX1 interacting proteins from blots shown in (A). Shown are mean ± standard deviation for *n =* 2–5 experiments. Statistical significance was determined using two-way ANOVA with Sidak post hoc correction for multiple comparisons. *P < 0.05, **P < 0.01.

Both known and putative ATOX1 interacting proteins were labeled by APEX2-ATOX1 constructs. Recent studies identified cysteine rich intestinal protein 2 (CRIP2) as a copper-binding transcription factor that interacts with ATOX1 and plays a role in copper dependent skeletal myoblast differentiation.^25,32^ CRIP2 was detected as an ATOX1 proximal protein in proteomic data and validated by immunoblotting independent streptavidin eluates from myoblasts and myocytes (Figure 4A and B). Several proteins detected in the previous ATOX1-APEX2-NLS study that identified CRIP2 were also detected as ATOX1 proximal proteins here including the nucleic acid binding proteins FUBP1, HNRNPAB, and SYNCRIP/HNRNPQ.^25^ Immunoblot analysis of streptavidin eluates revealed that SYNCRIP was most easily detected at the myocyte stage, though enrichment over minus biotin phenol negative control was not significant (Figure 4A and C). Interestingly, though no studies have shown a direct interaction between ATOX1 and MEK1/2 kinases, they have been functionally linked in the context of copper dependent MEK signaling.^21^ Both MEK1 and MEK2 were detected as APEX2-ATOX1 proximal proteins in myoblasts in comparative proteomics and confirmed by immunoblot (Figure 4A and D), suggesting that MEK1/2 kinases may interact with ATOX1 in muscle cells. Some known ATOX1 binding proteins were not detected in the APEX2-ATOX1 proximal proteome data including the copper chaperone for SOD1 (CCS), which can bind and undergo copper transfer with ATOX1.^33^ The lack of detection of CCS in proteomics experiment may reflect a minimal or transient interaction in the context of skeletal muscle cells in contrast to the in vitro interaction that was previously demonstrated. CCS interaction with APEX2-ATOX1 was detected by immunoblot in myoblasts and myocytes (Figure 4A and E), although the bands were faint, a result that is consistent with a transient or low abundance interaction. Similarly, ATP7A was not detected in proteomics data. The lack of ATP7A detection in comparative proteomics experiments is likely due to the use of boiling during elution from streptavidin beads, which is known to cause ATP7A aggregation. However, elution using urea containing buffer without boiling allowed for detection of significantly enriched ATP7A in myoblasts and myocytes (Figure 4A and F). ATP7A was also detectable in myotubes (Figure 4A), though enrichment over -BP control was not significant (Figure 4F). Taken together, these results suggest that while ATOX1 fulfills its well-established canonical functions in the intermediate stages of differentiation (myocytes), it may also participate in distinct regulatory processes in proliferating myoblasts and fully differentiated myotubes.

### ATOX1 deficiency impacts myoblast proliferation and differentiation

**Figure 5.**
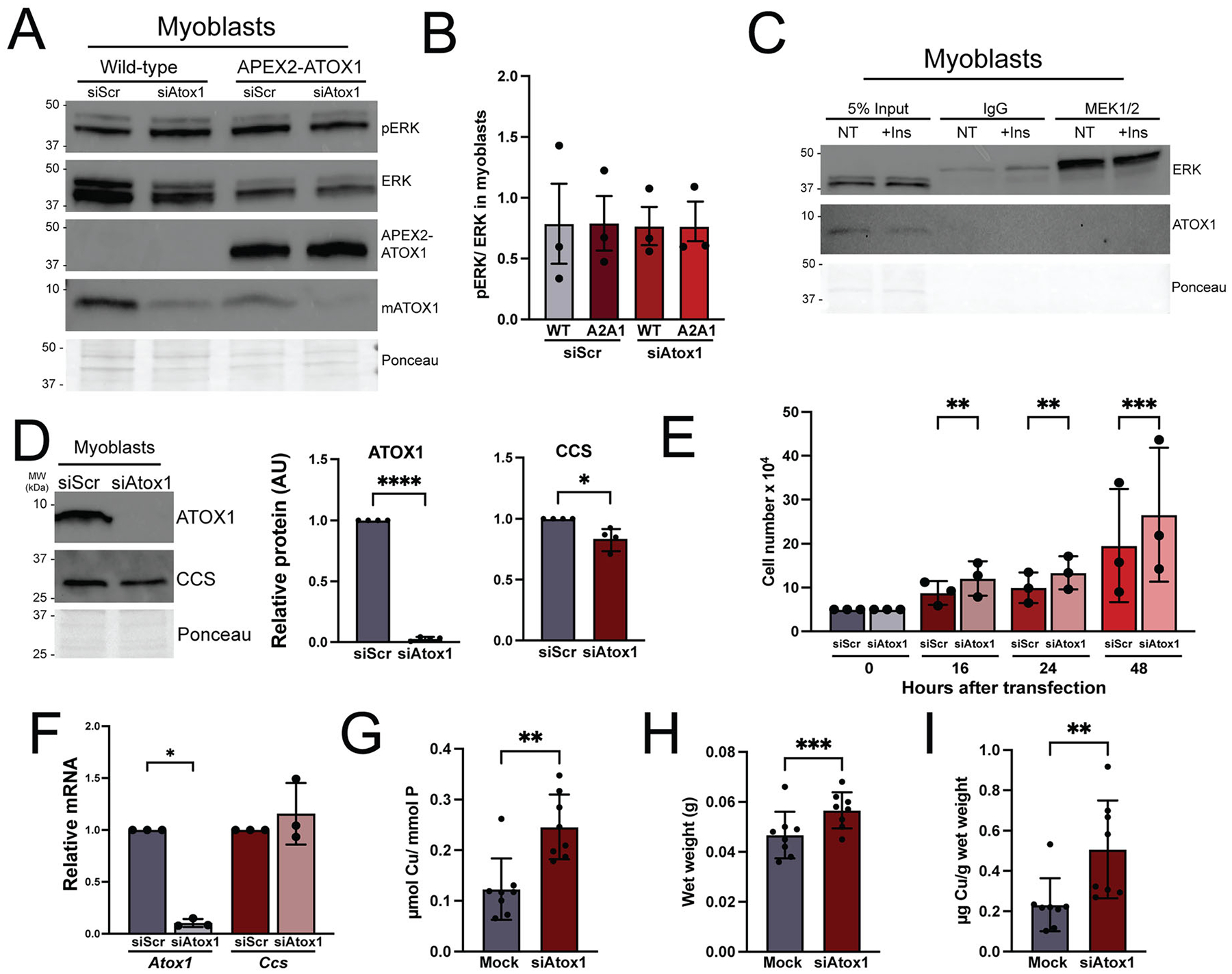
ATOX1 deficiency impacts proliferation but not ERK phosphorylation in myoblasts. (A) Immunoblot representative of *n* = 3 separate experiments showing phosphorylated ERK1/2 (pERK) and total ERK1/2 (ERK) in wild-type or APEX2-ATOX1 expressing myoblasts transfected with negative control siRNA (siScr) or *Atox1* targeting siRNA (siAtox1). Blots probed with an antibody to ATOX1 were used to show expression of APEX2-ATOX1 and endogenous ATOX1 knockdown (mATOX1). Total protein as detected by Ponceau stain was used as a loading control. (B) Ratio of pERK/ERK bands in wild-type (WT) and APEX2-ATOX1 (A2A1) expressing myoblasts with (siAtox1) and without (siScr) *Atox1* knockdown as measured by densitometry. Shown is mean ± standard deviation for quantification of *n=* 3 immunoblots. Statistical significance was determined using one-way ANOVA with Dunnett’s post hoc correction for multiple comparisons. None of the comparisons were statistically significant. (C) Immunoblot representative of *n =* 3 separate experiments showing immunoprecipitation (IP) using an antibody to MEK1/2 in wild-type myoblasts with (+Ins) or without (NT) insulin treatment to stimulate MEK signaling. The IP was probed with an antibody to ERK1/2 (ERK) showing interaction between MEK1/2 and ERK1/2 and an antibody to ATOX1 showing no interaction. Total protein as detected by Ponceau stain was used as a loading control. (D) Immunoblot representative of *n =* 4 separate experiments probed with an antibody to ATOX1 and an antibody to CCS in siScr and siAtox1 myoblasts. Quantifications showing reduced ATOX1 and CCS protein show mean ± standard deviation for *n =* 4 experiments. Statistical significance was determined by paired *t* test. ****P < 0.0001, *P < 0.05. (E) Proliferation as determined by cell counting in siScr and siAtox1 myoblasts as counted at 0, 16, 24 and 48 h after transfection. Shown is mean ± standard deviation for *n =* 3 experiments. Statistical significance was determined using two-way ANOVA with Sidak post hoc correction for multiple comparisons. *P < 0.05. (F) Quantitative PCR of *Atox1* and *Ccs* transcript levels in siScr and siAtox1 myoblasts showing no increase in *Ccs* levels. Shown is mean ± standard deviation for *n* = 3 experiments. Statistical significance was determined using two-way ANOVA with Sidak post-hoc correction for multiple comparisons. **P < 0.01, ***P < 0.0001. (G) Total Cu as measured by ICP-MS and normalized to total phosphorus (P) showing increased Cu in siAtox1 compared to siScr myoblasts. Shown is mean ± standard deviation for *n =* 8 experiments. Statistical significance was determined using paired *t* test. **P < 0.01. (H) Wet weights of myoblasts showing increased cell mass in siAtox1 compared to siScr myoblasts. Shown is mean ± standard deviation for *n* = 8 experiments. Statistical significance was determined using paired *t* test. ***P < 0.001. (I) ICP-MS measurements showing total Cu (μg) normalized to total wet weight of cells (g). Shown is mean ± standard deviation for *n* = 8 experiments. Statistical significance was determined using paired *t* test. **P < 0.01.

In myoblasts, functional annotation revealed enrichment for gene ontology terms related to cell cycle. In previous studies using mouse embryonic fibroblasts (MEFs) and BRAF^V600E^ mutant melanoma cell lines, copper was shown to promote proliferation by binding to and potentiating the activity of MEK1/2 kinases leading to increased phosphorylation of the downstream MEK targets ERK1/2 kinases.^34,35^ ATOX1 also promotes growth of BRAF^V600E^ expressing melanoma cells, though its effect on ERK1/2 phosphorylation was proposed to be indirect.^21^ In our study, both MEK1/2 kinases were detected as APEX2-ATOX1 proximal proteins in myoblasts (Figure 4A and Table 1). However, neither siRNA-mediated ATOX1 deficiency nor APEX2-ATOX1 overexpression affected MEK1/2 kinase activity as assessed by ERK1/2 phosphorylation in myoblasts (Figure 5A and B). Similarly, immunoprecipitation of MEK1/2 kinases enriched for ERK1/2 kinases, but did not yield any detectable ATOX1 protein, even in the presence of insulin stimulation of MEK/ERK signaling in myoblasts (Figure 5C). ATOX1 knockdown did, however, lead to a small but significant increase in myoblast growth as detected by cell counting (Figure 5D and E). ATOX1 deficiency led to a significant decrease in CCS protein levels (Figure 5D). This result suggests that ATOX1 deficient cells have increased copper levels or availability since CCS protein is inversely correlated with available copper and no change was detected in *Ccs* transcript levels (Figure 5F).^36,37^ Indeed, total copper levels were increased in ATOX1 deficient cells measured by inductively coupled plasma mass spectrometry (ICP-MS) and normalized to total phosphorus (Figure 5G). In agreement with increased proliferation in *Atox1* knockdown cells, we detected increased wet weights of ATOX1 deficient cells used for ICP-MS measurements (Figure 5H). Total copper was increased in ATOX1 deficient cells when normalized to wet weight (Figure 5I), confirming that phosphorus serves as an adequate internal control and verifying that increased copper is not due to increased cell mass. A very small but significant decrease in manganese was also detected but no changes in iron or zinc were noted (Supplemental Figure 2). These results suggest that ATOX1 deficiency promotes myoblast proliferation by increasing intracellular copper availability.

**Figure 6.**
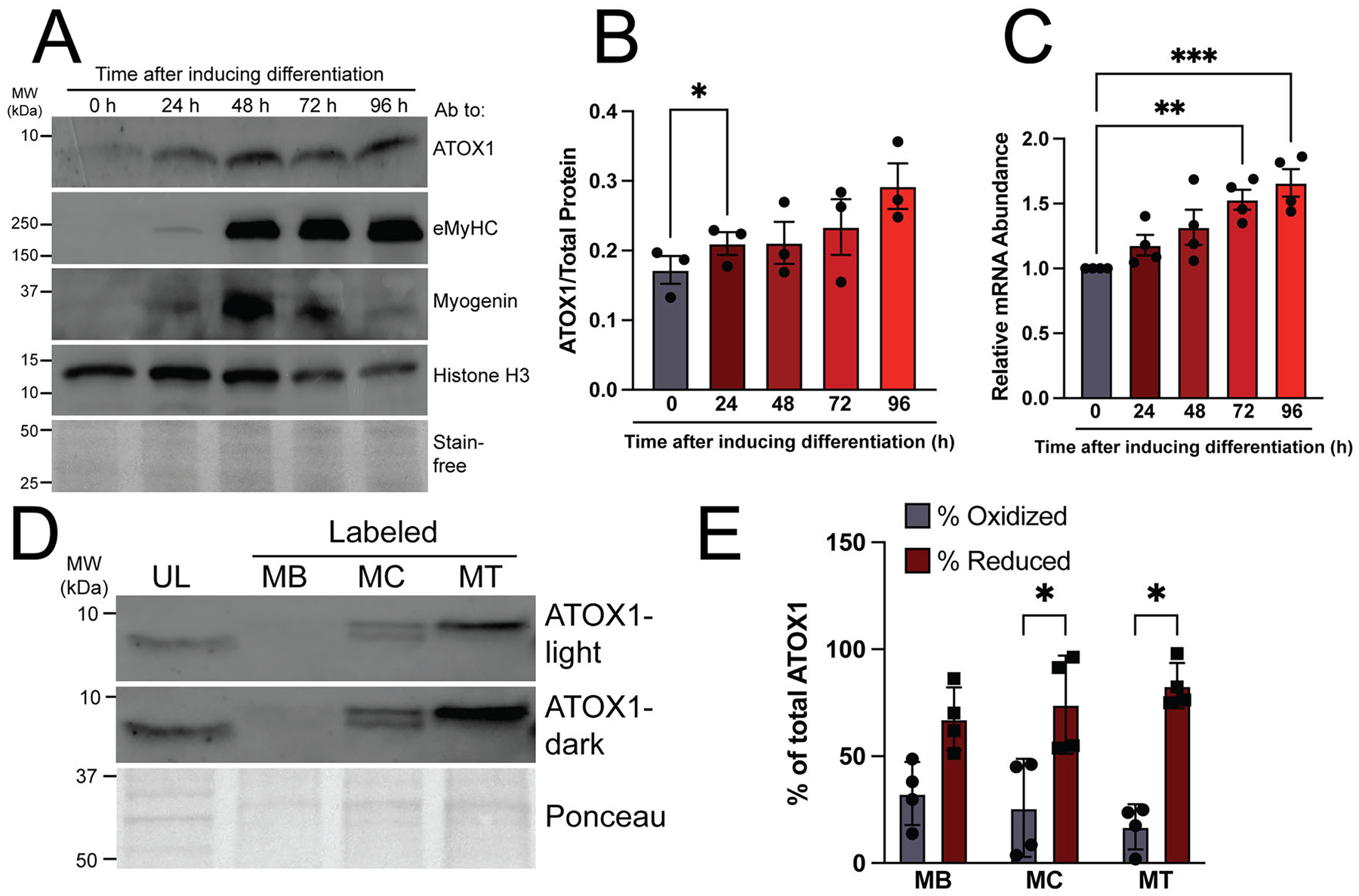
ATOX1 levels increase and redox state fluctuates during myoblast differentiation. (A) Immunoblot representative of *n* = 3 separate experiments showing increasing levels of ATOX1 at 24, 48, 72, and 96 h (h) after inducing differentiation corresponding to the myocyte (24–48), myocyte/early myotube (48–72), and mature myotube (72–96) stages. Myogenin and embryonic myosin heavy chain (eMyHC) used as intermediate and late-stage differentiation markers, respectively. Histone H3 and total protein as detected by Stain-free gel imaging technology (Bio-Rad) used as loading controls. (B) Quantification showing increased ATOX1 relative to total protein (Stain-free) during differentiation. Shown is mean ± standard deviation of immunoblot quantification for *n =* 3 experiments. Statistical significance was determined by one-way ANOVA with Dunnett’s post hoc test for multiple comparisons. *P < 0.05. (C) qRT-PCR data showing increased levels of *Atox1* transcript during differentiation. Shown is mean ± standard deviation for *n =* 4 experiments. Statistical significance was determined by one-way ANOVA with Dunnett’s post hoc test for multiple comparisons. **P < 0.01, ***P < 0.001. (D) Immunoblot representative of *n* = 4 separate experiments showing unlabeled (UL) and EZ-link maleimide PEG11-biotin labeled ATOX1 in myoblasts (MB), myocytes (MC), and myotubes (MT). Short (ATOX1-light) and long (ATOX1-dark) exposures are shown to highlight reduced (top band) and oxidized (bottom band) ATOX1 in myoblasts (MB), myocytes (MC), and myotubes (MT) lanes. Total protein as detected by Ponceau stain is used as a loading control. (E) Quantification of percent oxidized (bottom band) and reduced ATOX1 (top band) as calculated relative to total ATOX1 (top band + bottom band). Shown is mean ± standard deviation for *n =* 4 experiments. Statistical significance was determined by two-way ANOVA with Tukey’s post hoc testing for multiple comparisons. *P < 0.05.

Our group previously demonstrated that ATP7A levels increase dramatically during the early stages of myoblast differentiation before declining as cells mature into fully differentiated myotubes.^12^ Unlike ATP7A, levels of ATOX1 protein (Figure 6A and B) and RNA (Figure 6C) increase gradually throughout myoblast differentiation and remain high.^12^ Previous studies of ATOX1 revealed that its ability to bind copper is regulated by intracellular redox balance, which shifts ATOX1 to a more reduced state that is competent for copper binding during neuronal differentiation.^10^ Thiol labeling by EZ-link maleimide-PEG11-biotin allows for detection of reduced or oxidized thiols in ATOX1 by tracking the shift in molecular weight (~1 kDa) after labeling. There are three cysteine residues in ATOX1, two of which contribute to copper-bridged homodimerization and form a disulfide bond in an oxidizing environment, while the third cysteine is expected to remain free. Both labeled and unlabeled ATOX1 could be detected at each stage of differentiation, although the results were highly variable (Figure 6D). No overt change in labeled ATOX1 was observable across myoblast differentiation, but enrichment for labeled (reduced) compared to unlabeled (oxidized) ATOX1 was only significant in myocytes and myotubes as measured by densitometry quantification of the percentage of reduced (top) or oxidized (bottom) bands relative to total ATOX1 protein (top + bottom) (Figure 6E). These results suggest that thiol oxidation in ATOX1 fluctuates across differentiation states but may be enriched in more differentiated myocytes and myotubes.

**Figure 7.**
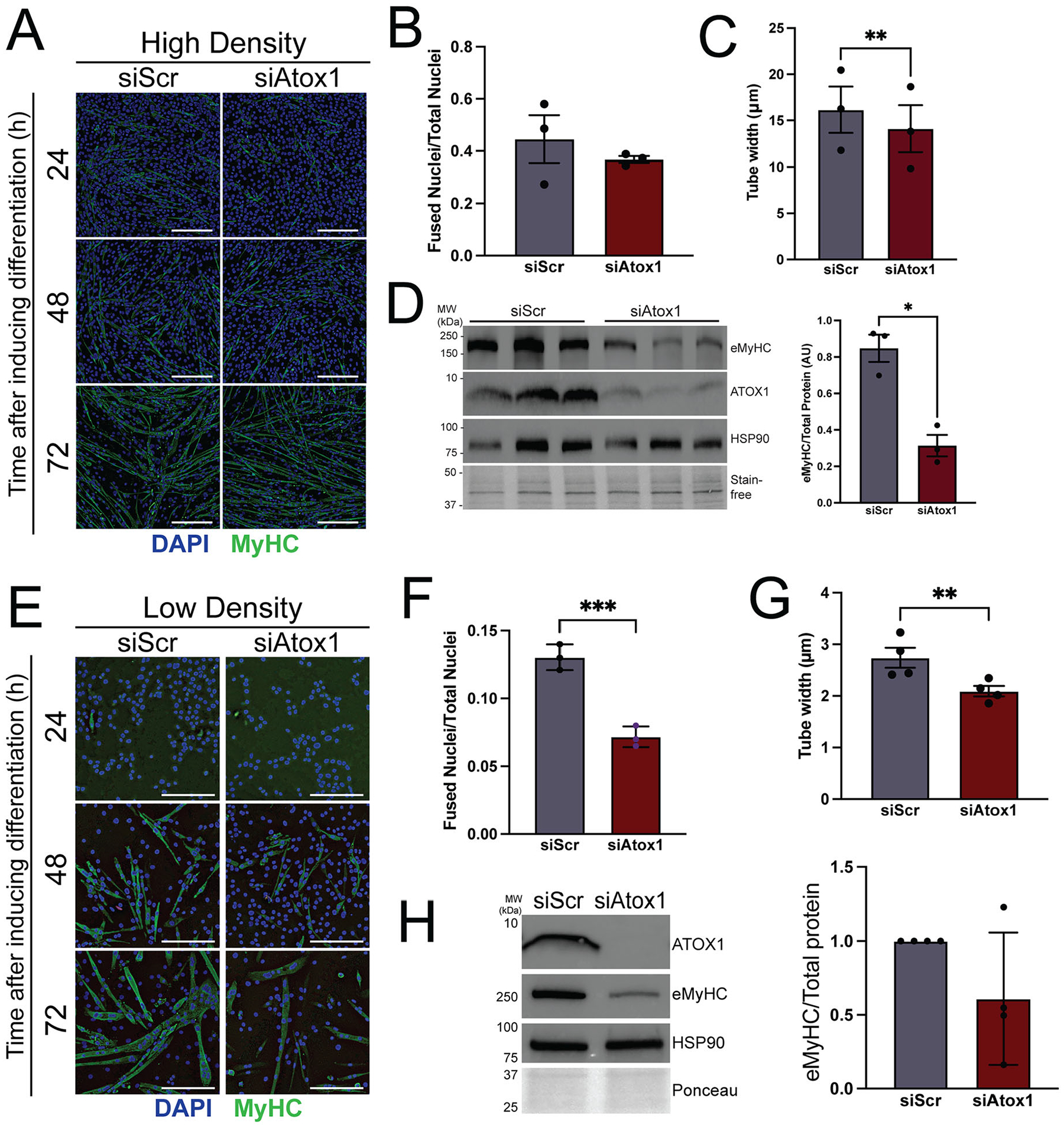
ATOX1 deficiency causes a cell density dependent differentiation defect. (A) C2C12 myotubes from control (siScr) or ATOX1 deficient (siAtox1) cells plated at high density. Myotubes are stained with an antibody to embryonic myosin heavy chain (eMyHC, green) and nuclei are stained with DAPI (blue) at 24, 48, and 72 h after differentiation representing myocytes, myocytes/early myotubes, and mature myotubes. (B) Fusion index (fraction of nuclei in myotubes relative to total nuclei) from high-density siScr and siAtox1 myotubes. (C) Tube width measurement from high density siScr and siAtox1 myotubes. (D) Immunoblot of lysates from high-density siScr and siAtox1 myotubes from *n =* 3 separate experiments probed with antibodies to eMyHC as a measure for differentiation and ATOX1 to show knockdown. HSP-90 and total protein detected using stain-free gel imaging technology (Bio-Rad) were used as loading controls. Shown are lysates from three independent experiments. Quantification shows levels of eMyHC normalized to total protein. (E) C2C12 myotubes from siScr and siAtox1 cells plated in low density and stained with an antibody to MyHC and DAPI at 24, 48, and 72 h after differentiation. (F) Fusion index from siScr and siAtox1 myotubes plated at low density. (G) Tube width from siScr and siAtox1 myotubes plated at low density. (H) Immunoblot representative of *n =* 4 separate experiments showing lysates from high-density siScr and siAtox1 myotubes probed with antibodies to eMyHC as a measure for differentiation and ATOX1 to show knockdown. HSP-90 and total protein as detected by Ponceau stain were used as loading controls. Quantification shows levels of eMyHC normalized to total protein. For all quantifications, shown is mean ± standard deviation for *n* = 3–4 experiments. Statistical significance was determined by *t* test. * *P* < 0.05, **P < 0.01, ***P < 0.001.

Our previous study revealed that ATP7A deficiency causes a marked defect in myotube formation by reducing activity of the secreted cuproenzyme LOX, but ATOX1 deficiency caused only minor perturbation of myoblast differentiation (Figure 7A and B).^12^ However, myoblast differentiation appeared to proceed slightly slower in ATOX1 deficient cells (Figure 7A). Mature myotubes were significantly thinner (Figure 7C) and levels of embryonic myosin heavy chain were reduced (Figure 7D) in ATOX1 deficient cells, suggesting that ATOX1 may be needed for late stages of differentiation such as secondary fusion or hypertrophy.^12^ It is possible that some residual copper transport by ATP7A exists in ATOX1 deficient cells and thus at high cell density there is sufficient activity of secreted cuproenzymes to promote differentiation. We hypothesized that a stronger phenotype would be observed by differentiating ATOX1 deficient myoblasts plated at lower density. Under these conditions, ATOX1 deficiency led to a significant impairment in myoblast differentiation as indicated by a reduced fusion index (Figure 7E and F), formation of smaller myotubes (Figure 7G), and a trend toward decreased embryonic myosin heavy chain levels (Figure 7H). To confirm the reduction in differentiation, we performed immunoblots using a pan-myosin heavy chain antibody (MyHC) and surprisingly detected variable degrees of reduction of total MyHC in *Atox1* knockdown myotubes when plated at high or low density (Supplemental Figure 3A and B), which may be due to detection of myosin heavy chain isoforms that are expressed across all stages of differentiation.^38,39^ Although ATOX1 proximal proteins differed substantially in fully mature myotubes compared to myoblasts or myocytes, knocking down *Atox1* after differentiation was completed did not produce any overt effect on myotube morphology (Supplemental Figure 3C to E). Taken together, these results support a model in which high cell density enables low levels of residual ATOX1 or ATOX1-independent copper delivery to ATP7A to sustain secreted cuproprotein activity and promote myotube formation.

## Discussion

In this study, we aimed to elucidate the role of ATOX1, the copper chaperone for ATP7A, during myoblast differentiation by characterizing its differentiation dependent protein interactions and activities. To this end, we generated stable C2C12 cells expressing an inducible APEX2-ATOX1 proximity labeling construct and discovered that the ATOX1 interactome undergoes dramatic changes as differentiation progresses. These shifting protein–protein interactions correlate with variable ATOX1 functions, wherein ATOX1 appears to suppress myoblast proliferation while promoting myoblast differentiation in a cell density dependent manner. The results reported here agree with a growing list of studies suggesting that variable ATOX1 regulation and activities contribute to tissue differentiation, development, and cancer.^10,21,24,40,41^

In proliferating myoblasts, ontologies related to cell cycle and insulin signaling, which included MEK1/2 kinases, were enriched in ATOX1-proximal proteins. The MEK/ERK signaling pathway is active in proliferating myoblasts but must decrease to promote differentiation into myotubes.^42^ ATOX1 has been implicated in promoting copper dependent MEK1/2 activity, though it was proposed to play an indirect role.^21^ Although ATOX1-MEK1/2 interactions were suggested by proximity labeling, we were unable to detect ATOX1 by reverse-immunoprecipitation using an antibody to MEK1/2. Given this discrepancy, it is possible that interactions between ATOX1 and MEK1/2 are transient or representative of small proportions of both proteins in myoblasts. In ATOX1 deficient myoblasts, no significant effect on MEK1/2 kinase activity was detected, though surprisingly, some of our data (Figure 5B and not shown) suggest a slight increase in phosphorylated ERK in *Atox1* knockdown cells. This result is likely due to increased copper availability in ATOX1 deficient cells. Indeed, total copper levels were increased upon *Atox1* knockdown in myoblasts, suggesting reduced copper export. Levels of CCS protein following *Atox1* knockdown decreased, a result that is consistent with increased copper as CCS protein stability is inversely correlated with copper availability.^36,37,43^ ATOX1 may also play additional undefined roles in regulating myoblast proliferation. ATOX1 proximal proteins in myoblasts also include negative regulators of the cell cycle like ERBB receptor feedback inhibitor 1 (ERRFI1) and transforming growth factor beta regulator 1 (TBRG1), both of which inhibit cancer cell growth.^44,45^ These results are contrasted with other studies that have identified a role for ATOX1 in tumor growth and metastasis in breast and colon cancers.^21,41,46,47^ However, considering that the ATOX1 proximal proteome shifts substantially even according to myoblast differentiation state, we propose a model wherein ATOX1 function is in part defined by cellular context.

A contextual model of ATOX1 function is supported by the fact that detection of ATP7A as an ATOX1 proximal protein was most significant in myoblasts and myocytes. This result agrees with the importance of ATP7A in early differentiation that we previously reported in muscle and others have reported in neuronal cells.^10,12,13^ The ATOX1 proximal proteins identified in myocytes by comparative proteomics include multiple proteins related to endosomal trafficking, cytoskeleton, and post-Golgi sorting, all of which are consistent with ATOX1 functioning to deliver copper to ATP7A. In fact, several ATOX1 proximal proteins were also detected in proximity labeling and standard immunoprecipitation studies to probe the protein binding partners of ATP7A including COPA, SNX9, and SCAMP3 (Supplementary Table 4).^48,49^ ATP7A itself was not detected since boiling during the streptavidin elution step renders the ATP7A protein insoluble. ATOX1 deficiency slowed differentiation and resulted in generation of smaller myotubes. This phenotype was exacerbated when myoblasts were plated at low density prior to differentiation, which is likely due to the role of ATOX1 in delivering copper to ATP7A for assembly into secreted cuproproteins like LOX.^12^ In contrast, cells plated at high density likely secrete sufficient levels of copper-bound and active cuproenzymes to promote formation of small myotubes. Thus, in intermediate stages of tissue differentiation, ATOX1 functions to promote copper delivery to tissue-specific and/or differentiation associated secreted cuproproteins via ATP7A.

Consistent with previous findings suggesting that ATOX1 can localize to the nucleus, several nucleic acid binding proteins were among the detected ATOX1 proximal proteins.^23,29,50^ Our findings agree with results from a previous study identifying the transcription factor CRIP2 as an ATOX1 and copper binding protein in a study that employed APEX2 tagged ATOX1 with an added nuclear localization signal (NLS).^25^ CRIP2 has also been reported as a copper responsive transcription factor essential for myoblast differentiation.^32,51^ Our data support a model wherein ATOX1, even in the absence of exogenous NLS, interacts with CRIP2 and other transcription factors in myoblasts and myocytes. Interestingly, a number of RNA binding proteins were also detected as ATOX1 proximal proteins in this work in line with the previous study of NLS-tagged ATOX1-APEX2. In our study, RNA binding proteins were particularly enriched in the ATOX1 proximal protein data set from myotubes. Although RNA binding proteins are sometimes identified as contaminants in proteomics experiments, they tend to be expressed at much lower levels in fully differentiated myotubes compared to myoblasts and myocytes, suggesting this result is not merely an artifact of protein abundance.^52–55^ Additional studies of ATOX1-RNA binding protein interactions are needed to better understand the importance of copper and ATOX1 in RNA binding protein function in skeletal muscle.

This study reveals shifting binding partners and differential functions of ATOX1 during myoblast differentiation. Some limitations should be noted, including the potential for detection of nonspecific binding partners and the use of hydrogen peroxide for APEX2 labeling, which may disrupt copper binding or transfer from ATOX1. However, the ATOX1-ATP7A interaction, which likely requires copper, is readily detectable using APEX2-ATOX1, suggesting that copper transfer function by APEX2-ATOX1 remains intact.^29,56^ Furthermore, detection of the expected ATOX1 localization and known binding partners strengthens our confidence in the results reported here. The results presented here establish dramatic shifts in ATOX1 interactors and function across different stages of myoblast differentiation and include a useful data set of potential ATOX1 binding proteins for future studies. Taken together, these data provide a window into the context-dependent function of ATOX1 in a model of mammalian tissue development.

## Materials and Methods

### Growth and differentiation of cell lines in culture

C2C12 immortalized skeletal myoblasts (purchased from American Type Culture Collection [ATCC]) and HEK293T cells (a kind gift from Dr Bill Miller, University of Cincinnati) were grown in growth medium (GM) consisting of Dulbecco’s modified Eagle medium (DMEM, Corning, MT10013CV) in the presence of 10% fetal bovine serum (FBS, Cytiva SH3091003), 50 μg/mL penicillin and streptomycin (pen/strep, Corning MT30001CI), and 2.5 μg/mL Plasmocin treatment (InVivogen, NC9698402) at 37 °C in 5% CO_2_. C2C12 myoblasts were induced to differentiate by seeding 2.0 × 10^5^ cells/well in a 6-well plate and growing to about 90% confluency in GM then switching to differentiation medium (DM) consisting of DMEM with 2% horse serum (Cytiva, SH3007403) and 50 μg/mL pen/strep and incubating at 37 °C in 5% CO_2_. Myocytes were harvested after 48 h of incubation while myotubes were harvest after 72–96 h of incubation once formation of large myotubes in control plates was clearly visible. To generate myotubes from low density cells, C2C12 myoblasts were plated at 1.2 × 10^5^ cells/well in a 6-well plate and allowed to grow to ~50% confluence and induced to differentiate. For cell counting, 5 × 10^4^ cells were plated in a 6-well plate, transfected with control or *Atox1* targeting siRNA and counted at 16, 24, and 48 h after inducing *Atox1* knockdown.

### Plasmids and cloning

The sequence encoding the APEX2-ATOX1 construct was synthesized by Azenta/Genewiz and subcloned from commercial vector into pcDNA 3.1 (ThermoFisher V79020) and then into the lentiviral vector pCW57.1 using EcoRI restriction enzyme recognition sites. The pCW57.1 was donated to Addgene by Dr David Root (Addgene plasmid # 41393; http://n2t.net/addgene:41393; RRID:Addgene_41393). Plasmids were confirmed by Sanger sequencing and whole plasmid sequencing prior to use.

### Immunoblot analysis

Plated cells were washed 3x in ice-cold phosphate buffered saline (PBS: 137 mM NaCl, 2.7 mM KCl, 10 mM Na2HPO4, 1.8 mM KH2PO4, Fisher: BP665–1). Cells were then scraped into radio immunoprecipitation assay (RIPA) lysis buffer (25 mM Tris HCl pH 7.6, 150 mM NaCl, 1% NP-40, 1% sodium deoxycholate, 0.1% SDS) supplemented with protease inhibitor (Pierce, PIA32953). For lysates collected for analysis of phosphorylated ERK, RIPA buffer was also treated with phosphatase inhibitor cocktail (Sigma Aldrich P2850). Total protein in lysates was quantified by Bradford assay (Bio-Rad, 5000205) according to the manufacturer’s instructions. All immunoblots were loaded with 10 μg of protein unless otherwise noted. Blots probed with pan-myosin heavy chain antibody were loaded with 30 μg of protein. Protein samples were separated on 4–20% Mini-Protean^®^ TGX^™^ stain free protein gels (Bio-Rad, 4568094) and then transferred to nitrocellulose membrane (Cytiva, 45–004–001) using a Bio-Rad Transblot (1704150) following the manufacturer’s instructions. Total protein transferred was observed using Stain-free gel imaging technology (BioRad) or Ponceau staining (Thermo A40000278). Membranes were blocked for 5–10 min in EveryBlot blocking buffer (Bio-Rad 12010020) at room temperature with agitation. Blots were incubated with appropriate primary antibody resuspended in tris buffered saline with Tween-20 (TBST: 20 mM Tris, 150 mM NaCl, 1% Tween-20, pH 7.6) for 1 h at room temperature or overnight at 4 °C (antibody information can be found in Supplementary Table 1). Primary anti-ATP7A was incubated in 5% nonfat dry milk in TBST overnight. After primary antibody incubation, membranes were washed three times for 5 min with TBST before incubation in appropriate secondary antibody for either 1 h at room temperature or overnight at 4 °C. Membranes were then washed three times for 5 min with TBST and developed with either Pico Plus ECL substrate (Thermo, PI34580) or Femto Plus ECL substrate (Thermo, PI34095) for 1 min at room temperature before being visualized with a Bio-Rad Chemi-doc imager. Relative protein expression was quantified by densitometry using Bio-Rad Image Lab software.

### Generation of doxycycline-inducible APEX2-ATOX stable cell line

Six-well plates were treated with Matrigel (Corning, 354234) diluted in DMEM for 1 h at ambient temperature and washed with PBS. HEK293T cells were seeded at 3.5 × 10^5^ cells/well and incubated overnight at 37 °C with 5% CO_2_. Approximately 16–18 h after seeding, medium on HEK 293 T cells was changed to growth medium without penicillin-streptomycin (transfection medium). Plasmids encoding APEX2-ATOX1 were co-transfected alongside PsPax2, a packaging construct containing HIV GAG-Pol, and PMDG.2, a viral envelope expressing plasmid containing VSVG envelope at a ratio of 4:2:1. After approximately 15–16 h, transfection medium was removed, and growth media was added to each well. To test for transfection efficiency, puromycin selection was used by adding puromycin (Gibo A1113803) at a final concentration of 2 μg/mL to each well. Approximately 24 h after addition of puromycin, low passage C2C12 myoblasts were seeded into 100 mm plates at low density. Then, 48–72 h later, enriched medium was removed from HEK293T cells and immediately placed on ice. Fresh growth medium was added back onto HEK293T cells to collect and store a second round of virus production. Enriched medium was centrifuged twice at 500 *g* for 5 min to remove cellular debris, moving the supernatant to a fresh tube after the first spin, and further clarifying by filtration through a Whatman PES 0.45 μm syringe filter. In growth medium containing 8 μg/mL final polybrene (Thermo TR1003G) 3 mL of enriched medium containing lentiviral particles were added. Remaining vector was frozen overnight in a Styrofoam container at −80 °C and moved to liquid nitrogen the following day. To ensure proper distribution of vector, plates that received enriched medium were rocked back and forth repeatedly every 30 min for 8 h. Approximately 24 h after addition of APEX2-ATOX1 containing vector, fresh growth medium containing puromycin (2 μg/mL final) was added onto transduced C2C12 myoblasts and again 48 h after transduction. Selection pressure was maintained by treating with puromycin (2 μg/mL final) for 48 h after every passage or upon thawing fresh cell stocks.

### Induced expression of doxycycline inducible APEX2-ATOX1 and labeling

For labeling experiments in proliferating myoblasts, transduced cells were grown to approximately 60% confluency in GM. Doxycycline (Fisher, BP26535) was then added to a final concentration of 2.5 μg/mL (optimal concentration determined through titration shown in Figure 2) and incubated 24 h. Cells were then incubated in GM with biotin-phenol (Sigma, SML2135–50MG) at a final concentration of 2.5 mM for 30 min at 37 °C in 5% CO_2_. After incubation, cells were washed twice with sterile PBS then incubated in 0.75 mM H_2_O_2_ (Fisher, H325–500) in PBS for exactly 1 min. The reaction was quenched by washing cells twice with stop/wash buffer (PBS supplemented with 10 mM MgCl_2_, 20 mM CaCl_2_, 10 mM NaN_3_, 5 mM Trolox, 10 mM sodium ascorbate) followed by two more washes of sterile PBS before total cell lysates were collected in 250–500 μL of RIPA lysis buffer for analysis. Labeling experiments in myocytes and myotubes followed the same protocol as described above with the following modifications. Transduced cells for myocyte experiments were grown to approximately 90% confluency in GM before being switched to DM for 24 h before doxycycline treatment. Transduced cells for myotube experiments were grown to 90% confluency in GM before switching to DM for 72–96 h before doxycycline treatment.

### Cell fractionation

Medium was aspirated from cells and then washed once with cold PBS. Cells were then scraped into 1 mL of cold PBS with a rubber scraper and centrifuged for 5 min at 500 *g*. After centrifugation, supernatant was removed and 100 μL of NP-40 lysis buffer (50 mM Tris HCl pH 7.4, 150 mM NaCl, 1% NP-40, 5 mM EDTA) was added and mixed by gently pipetting up and down. Cells were then incubated for 5 min on ice with occasional gentle mixing. After incubation, 30–40 μL of total lysate was collected as the input fraction while the remaining cell lysate was centrifuged for 5 min at 500 *g*. After centrifugation, the supernatant was collected as the non-nuclear/fraction. The remaining nuclear fraction pellet was washed with 100 μL of cold PBS by gently pipetting up and down and then centrifuged for 5 min at 500 *g*. The supernatant was removed, and the nuclear pellet fraction was resuspended in 60–70 μL of RIPA lysis buffer. All samples were then either frozen at −80 °C for long-term storage or sonicated on ice with an ultrasonic membrane disruptor (Fisher Model 100) twice for 5–10 s each and then centrifuged for 30 min at 21,000 *g* in preparation for SDS-PAGE and immunoblotting.

### Streptavidin affinity precipitation and analysis

Total cell lysates were scraped into 250 μL of RIPA lysis buffer supplemented with protease inhibitor and incubated on ice for 30 min. Cell lysates were sonicated on ice with an ultrasonic membrane disruptor (Fisher Model 100) twice for 5–10 s each and centrifuged at 21,000 *g* for 30 min. Supernatants were transferred to new tubes and protein concentration was determined through Bradford assay according to the manufacturer’s instructions (Bio-Rad, 5000205). 100 μL of Pierce high-capacity streptavidin agarose beads (PI20357) per sample were washed twice with 500 μL of IP buffer (50 mM Tris HCl pH 7.5, 150 mM NaCl, 5 mM MgCl_2_, 1% NP-40) supplemented with protease inhibitor with rotation for 5 min. Beads were pelleted by centrifugation for 1 min at 500 *g* and blocked for 2–3 h in 500 μL of 1 × IP blocking buffer (IP Buffer + 1% bovine serum albumin (Fisher: BP9703–100) at 4 °C. Beads were pelleted by centrifugation and then washed twice for 5 min in IP Buffer. Then 500 μg of protein lysates were added to agarose beads and incubated overnight with rotation at 4 °C. After incubation, bead slurry was washed five times for 5 min with 500 μL of IP buffer with rotation. Proteins were eluted from beads by adding 50 μL of 2X Laemmli sample buffer (Bio-Rad, 161–0747) and 50 μL of IP buffer supplemented with 6.5 M urea (Fisher, 434720010) followed by incubation at 95 °C for 5 min. Total protein recovered was evaluated via silver stain of SDS-PAGE gels according to the manufacturer’s instructions (Thermo-Fisher, 24612) and visualized with a Bio-Rad Chemi-doc imager. Biotinylation efficiency was determined via immunoblot analysis using streptavidin-HRP (Supplementary Table 1).

### LC-MSMS for ATOX 1 proximity profiling and functional annotation

Streptavidin eluates from APEX2-ATOX1 expressing myoblasts, myocytes and myotubes from three independent experiments were used for proteomic profiling. Cells without biotin phenol treatment were used as negative controls. Enriched biotinylated proteins in Laemmli sample buffer were prepared for and subjected to in-gel trypsin direction and recovery followed by a label-free quantitative nanoLC-MS/MS workflow as detailed previously.^57^ Briefly, samples were all run approximately 2 cm into an Invitrogen 4–12% Bis-Tris gel using MOPS buffer with pre-stained molecular weight marker lanes in between. The full 2 cm protein sections were excised, reduced with dithiothreitol, alkylated with iodoacetic acid, and digested overnight with trypsin. The peptides were subsequently recovered followed by nanoLC-MSMS on a Dionex Ultimate 3000 RSLCnano coupled to a Thermo Orbitrap Eclipse mass spectrometry system. Data were collected using Xcaliber 4.3 software (Thermo Scientific) with label free quantitation comparative profiling of proteins detected from each sample group achieved using Proteome Discoverer 3.0 (Thermo Scientific) against the complete *Mus musculus* protein database. Proteins with the minimum of 2 high (99%) confidence peptides with significant proteins differences (*P* < 0.05) and minimum of 2-fold change between the groups are reported. Proteins detected in all three replicates and not detected in -BP controls were used for downstream functional annotation using DAVID and STRING databases.

### Crosslinking reverse co-immunoprecipitation

C2C12 cells were grown to approximately 80% then washed with 1× PBS twice and crosslinked with 0.1 mM DSP (Thermo 22585) in PBS for 30 min at 37 °C. Cells were rinsed twice with PBS and the reaction quenched with 20 mM Tris-HCl (VWR 1185–53–1) for 15 min at room temperature. Cells were then rinsed twice more with 1× PBS and scraped into 500 μL of RIPA lysis buffer for total cell lysate preparation as described below. Total cell lysate protein concentration was calculated using Bradford assay. Protein A agarose beads (Pierce 22810) were used for immunoprecipitation of MEK1/2 (Supplementary Table 1). 35 μL of beads were rinsed twice with IP buffer and then incubated with MEK1/2 antibody according to manufacturer recommendations overnight with rotation at 4 °C. Protein lysates (250–500 μg) were precleared in 25 μL of rinsed beads for 1 h at 4 °C. After preclearing, cell lysates were incubated with bead-antibody mixtures overnight at 4 °C with rotation. Beads were washed with 500 μL of IP buffer five times for 5 min each then incubated with 50 μL of 2× Laemmli buffer (BioRad 161–0747), 45 μL IP buffer, and 5 μL of 1 M dithiothreitol (DTT) for 30 min at 37 °C with periodic shaking. Bead mixture was then boiled for 5 min and then eluates were removed for analysis via immunoblot according to the protocol described below.

### Transfection and siRNA knockdowns

Knockdown of *Atox1* was performed a previously described.^12^ Briefly, dicer substrate siRNAs targeting the *Atox1* 3^′^ untranslated region (UTR) were purchased from IDT. Myoblasts growing in transfection medium (growth medium without penicillin-streptomycin) were transfected with *Atox1* targeting siRNAs or non-targeting negative control siRNAs using Lipofectamine 3000 (Thermo Fisher L3000008). Transfecting cells were refreshed with fresh growth medium or differentiation medium without transfection mix after ~16 h in transfection mix. Knockdown of ATOX1 was confirmed by immunoblot.

### Quantitative reverse transcriptase PCR (qRT-PCR)

RNA was isolated using TRIzol (Invitrogen 15596018) according to manufacturer’s instructions. cDNA was synthesized using the Maxima First Strand cDNA Synthesis kit (Fisher K1672) according to manufacturer’s instructions. Quantitative reverse transcriptase PCR was then performed using SYBR Select Master Mix (Applied biosystems 4472919) on a QuantStudio 3 Real-Time PCR System (Applied Biosystems) using primers targeting *Atox1* (F: *cgagttctccgtggacatga* R: *cctcccagcttgttgaggac*), *Ccs* (F: *gtgttggtgcagacgactct* R: *tagctgtaggaagcggacca*) and *Rplp0* (F: *gggcgacctggaagtccaact* R: *cccatcagcaccacagccttc*). Results were quantified using comparative Ct method with *Rplp0* as a normalizer.^58^

### Inductively coupled plasma mass spectrometry (ICP-MS) of *Atox1* knockdown cells

Myoblasts were grown to confluence in 100 mm dishes and treated with mock transfection mix (Mock) or mix containing *Atox1* targeting siRNAs (siAtox1). Cells were harvested after 48 h by washing three times in sterile saline in ultrapure water (Ricca Chemical 9150) in acid-washed tubes and cells pelleted by centrifugation at 800 *g* for 5 min. Wet weights of all cell pellets were measured and recorded. Cell pellets were digested in 150 μl of 70% trace metals grade nitric acid (Fisher Chemical A509P500) at 60 °C for 4 h, centrifuged at high speed, and resulting supernatant was diluted in ultrapure water to 3 mL final volume. Total metal levels were quantified using an Agilent 7850 ICP-MS using RF power 1550 W, sample depth 8.0 mm, nebulizer flow 1.05 L/min, spray chamber temperature 2 °C, extraction lens 1, 2–0, −190.0 V) with argon used as primary reaction gas and helium used as secondary gas flow to minimize polyatomic interference (at 3.0 mL/min). Metals were quantified based on external calibration using dilution series of 0, 0.5, 2, 10, and 20 ppb for copper, iron, zinc, and manganese and 0, 1.5, 5, 20, 125 ppm for phosphorus (Agilent 5190–8348, 8472, 8557, 8483, 8499). Germanium was used as an internal standard for copper, zinc, phosphorus and scandium was used as an internal standard for iron and manganese (Agilent 5191–4570). All ICP-MS analysis included analysis of drinking water certified reference material (National Research Council of Canada AQUA-1) with copper measurements detected within 25% of the accepted value. Data were analyzed using Agilent MassHunter Software v. 5.2 and calculations were performed to normalize metals to total phosphorus (internal control) or wet weight of cell pellet (external control) using Microsoft Excel.

### EZ-link maleimide labeling

Maleimide labeling of reduced cysteines in ATOX1 was performed as previously described.^10^ Cells were grown to desired stage of differentiation then scraped into MOPS lysis buffer (50 mM MOPS pH 7,500 mM sucrose, 300 mM NaCl) and incubated on ice for 10 min. Cells were then sonicated and protein concentration was determined by Bradford assay. Oxidized control sample was prepared by incubating lysate in 1 mM H_2_O_2_ for 5 minutes at room temperature before labeling. Samples were labeled by adding 1 μL of 250 mM EZ-link Maleimide (Thermo 21911) stock reagent to 99 μL of protein lysates for a final concentration of 2.5 mM. Mixtures were incubated on ice for 3 h and then quenched by addition of 10 μL of 1 M cysteine. 10–15 μg of labeled protein lysates were analysis via immunoblot.

### Immunofluorescence staining

Cells were washed twice with PBS and fixed with 3.7% formaldehyde (Fisher, BP531500) for 10 min at room temperature. Cells were washed twice with PBS then permeabilized in PBS supplemented with 0.3% Triton-X (Fisher, BP151 100) by incubating at room temperature for 15 min. After incubation, cells were rinsed once with PBS and incubated with IF Blocking buffer (PBS supplemented with 0.1% Triton-X and 3% bovine serum albumin) for 1–2 h at 4 °C. After blocking, cells were rinsed once with PBS then incubated with primary antibody diluted in 0.5X IF blocking buffer overnight at 4 °C (antibody information can be found in Supplementary Table 1). After incubation cells were washed three times with 0.5X IF blocking buffer then incubated with secondary antibody diluted in 0.5X IF blocking buffer for 1–2 h at room temperature protected from light. After incubation cells were washed three times with 0.5X IF blocking buffer then incubated with 4^′^,6-diamidino-2-phenylindole (DAPI: Fisher, D3571) diluted 1:1000 in PBS for 5–10 min. After incubation, cells were washed twice with PBS and kept in fresh PBS while imaging. Cells were imaged with an Olympus IX83 inverted fluorescence microscope using a U Plan fluorite 10× objective lens (NA 0.3 WD 10 mm). Images were captured using a DP74 Color CMOS Camera (cooled 20.8 MP pixel-shift, 60 FPS) using CellSens Dimension V2 software. Images were subjected to 2D-deconvolution and exported as red, green, blue (RGB) TIF files for analysis in FIJI. Three images were taken at random positions for each sample and experiments were completed in triplicate.

### Quantification of differentiation

Fully formed myotubes were stained with anti-eMyHC and DAPI. Three images were taken at random positions in the well for each sample and exported as red, green, blue tiff images to FIJI. Fusion index was determined by calculating the ratio of nuclei in multinucleated eMyHC positive cells to total nuclei in each image. Fusion index calculations from each image were averaged and analyzed by ANOVA in GraphPad Prism v. 10.4.0. Myotube width was measured in FIJI by choosing 10 myotubes randomly for each image and quantifying width in nanometers. Fusion index and myotube width analyses were performed by investigators blinded to the identity of the samples being analyzed.

### Statistical analysis

Statistical analysis was performed using GraphPad Prism 10 for macOS. For all quantitation, figures show mean ± standard deviation. Unless otherwise noted, for experiments comparing one variable in two groups, *t* test was used for statistical comparison. One-way ANOVA with Dunnett’s post hoc correction for multiple comparisons was used when comparing three or more groups. Experiments comparing more than one related variable in two or more groups, two way ANOVA was used with Tukey or Sidak post hoc correction for multiple barriers as noted. In all cases *P* < 0.05 was considered statistically significant. For all comparisons, number of replicates, statistical test, and *P* value ranges were noted in the figure legends.

## Supplementary Material

Supp 1

Supplemental data for this article can be accessed online at https://doi.org/10.1080/10985549.2026.2621941.

## Figures and Tables

**Table 1.** Summary of proteomics data for key candidate ATOX1 proximal proteins

Protein	Average Abundance		
	Myoblasts	Myocytes	Myotubes
CRIP2	10606072.8	21899884.8	N/A^a^
MEK1	2827168.06	N/A^a^	N/A^a^
MEK2	1436630.71	N/A^a^	N/A^a^
SYNCRIP	4122848.08	4488650.63	7127159.27

a N/A: protein did not meet inclusion criteria for these samples.

## Data Availability

Proteomics data used for this study openly available on figshare https://doi.org/10.6084/m9.figshare.29497352.v1
