# Supplementary material for: The Copper Chaperone ATOX1 Exhibits Differential Protein–Protein Interactions and Contributes to Skeletal Myoblast Differentiation
Source: Mol Cell Biol. Author manuscript; Available in PMC 2026 Mar 14. (PMC12983349; doi:10.1080/10985549.2026.2621941)
Supplement: Supp 1 [file NIHMS2149681-supplement-Supp_1.pdf]

Supplemental material

The copper chaperone ATOX1 exhibits differential protein-protein interactions and contributes to skeletal myoblast differentiation

Nathan Ferguson [a], Yu Zhang [a], Alexandra M. Perez [a], Allison T. Mezzell [a], Jason Fivush [a], Vinit C. Shanbhag [b], Michael J. Petris [c], and Katherine E. Vest [a]*

[a]Department of Molecular and Cellular Biosciences, University of Cincinnati, Cincinnati, Ohio USA; [b]Department of Biochemistry and Christopher S. Bond Life Sciences Center, University of Missouri, Columbia, Missouri, University, City, Country [c]Departments of Biochemistry, Molecular Microbiology and Immunology, Ophthalmology, and Christopher S. Bond Life Sciences Center, University of Missouri, Columbia, Missouri, University, City, Country

*Corresponding author: Katherine E. Vest, katherine.vest@uc.edu

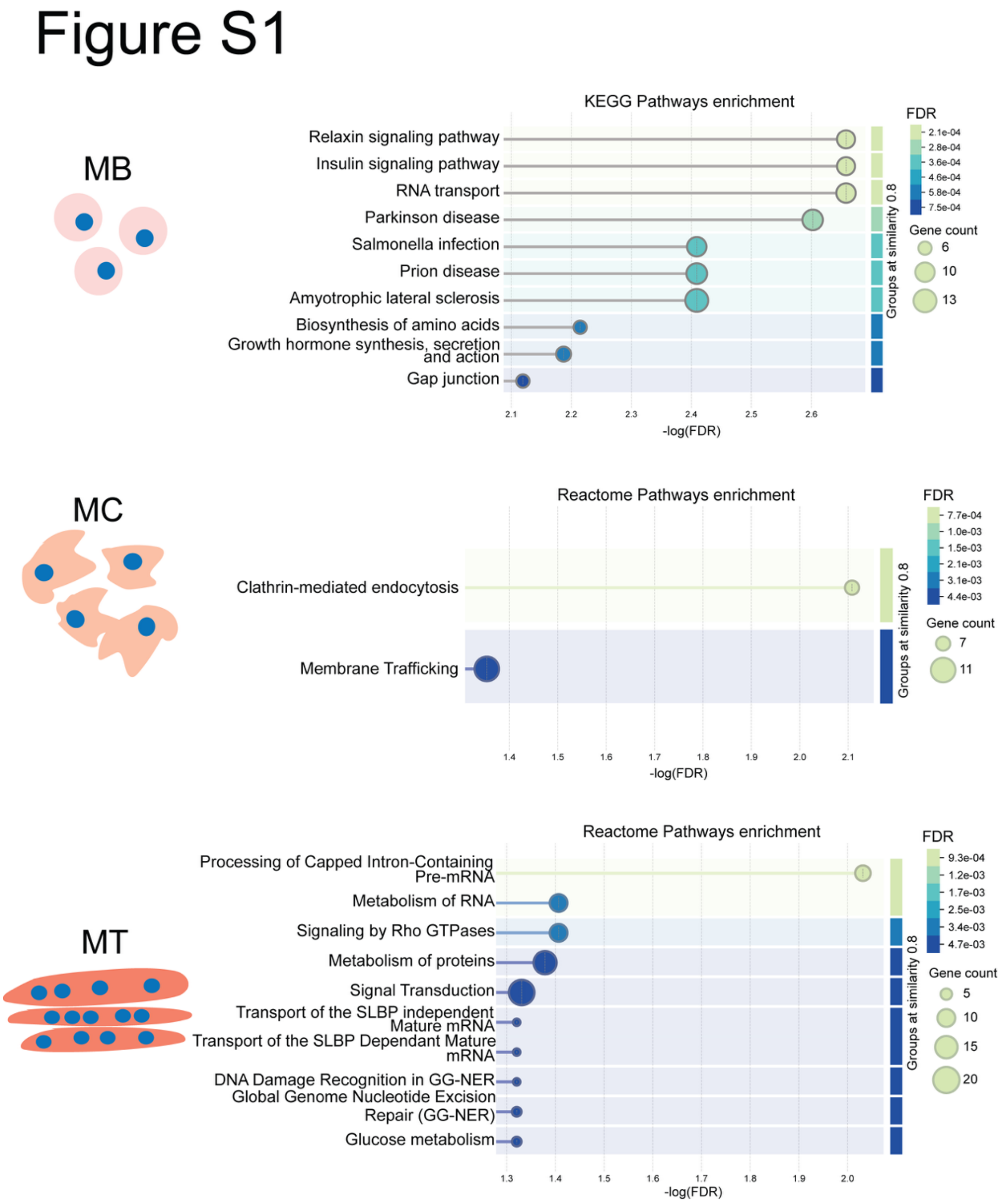

Supplemental Figure 1: Additional functional annotation of ATOX1 proximal proteins
Shown are enriched KEGG pathway in myoblasts (MB) and Reactome pathways in myocytes (MC) and myotubes (MT). No KEGG pathway enrichment was detected in MC or MT and no Reactome pathway enrichment was detected in MB. Pathway analysis was performed using the STRING database.

Supplemental Figure 2

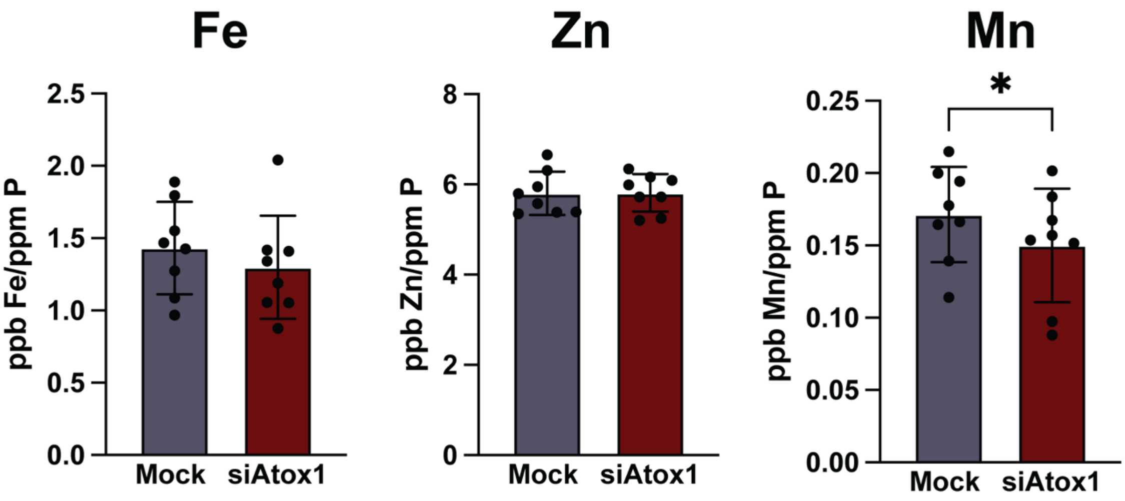

Supplemental Figure 2: ICP-MS measurements of other metals in *Atox1* knockdown myoblasts Total iron (Fe), zinc (Zn), and manganese (Mn) as measured by ICP-MS and normalized to total phosphorus (P) showing no change in Fe or Zn and a small but significant decrease in Mn in *Atox1* knockdown (siAtox1) compared to control (siScr) myoblasts. Shown is mean ± standard deviation for n = 8 experiments. Statistical significance was determined using paired t-test. * $p < 0.05$.

Figure S3

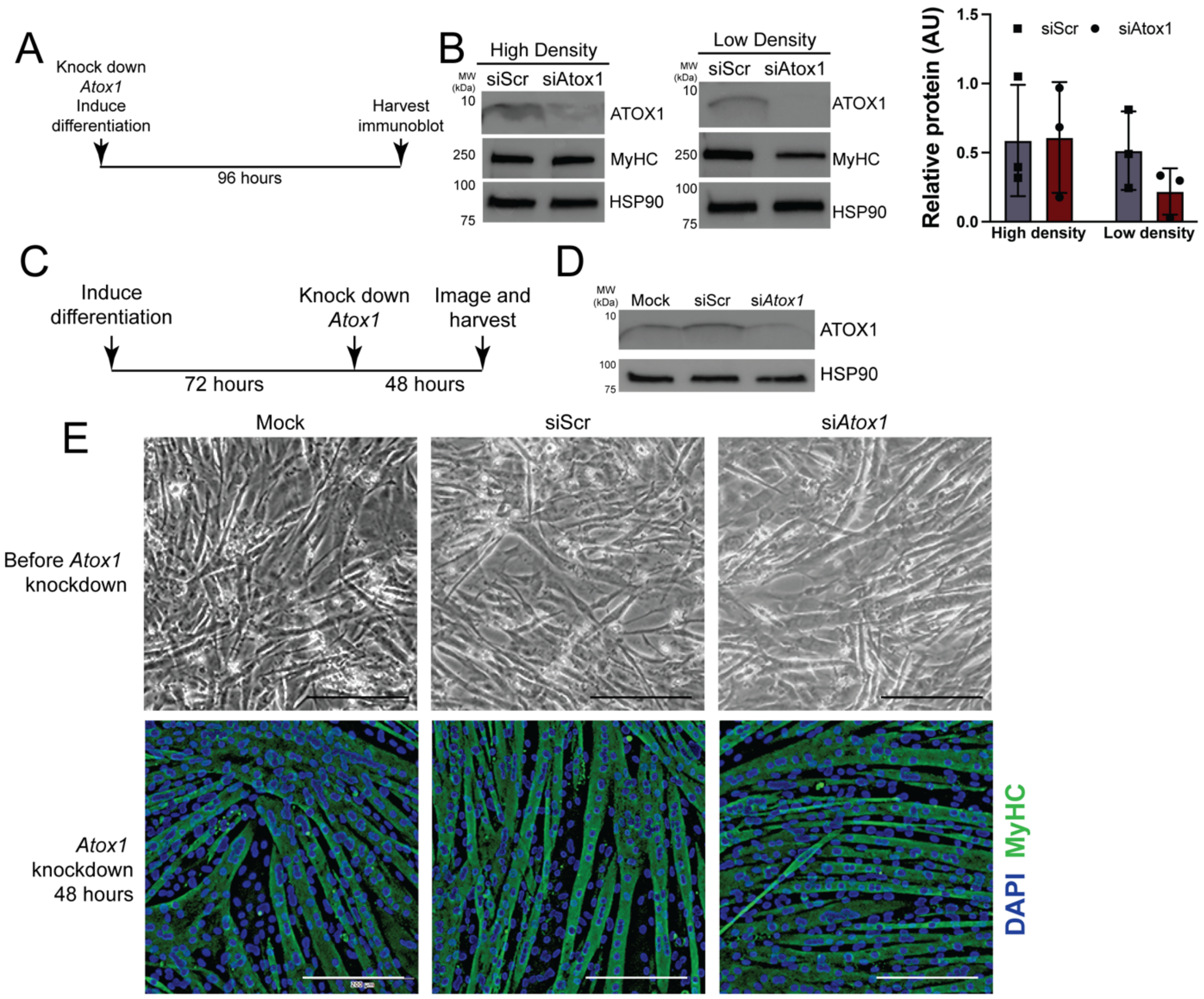

Supplemental Figure 3: Effect of ATOX1 knockdown on myotubes ***A)*** Schematic of *Atox1* knockdown induced prior to differentiation. ***B)*** Immunoblots and quantification for n = 3 separate experiments probed with a pan-myosin heavy chain antibody (MyHC) in *Atox1* knockdown myotubes after plating at high density (left) and low density (right) showing variable levels of MyHC. ***C)*** Schematic of experiment in fully differentiated myotubes. Briefly, myoblasts were allowed to differentiate for 72 hours, transfected with control or *Atox1* targeting siRNA (siAtox1), and harvested 48 hours later. ***D)*** Immunoblot representative of n = 3 separate experiments probed with an antibody to ATOX1 showing reduced ATOX1 protein in *Atox1* knockdown myotubes compared to mock transfected (Mock) or non-targeting siRNA (siScr) control myotubes. Antibody targeting HSP90 was used as a loading control. ***E)*** Phase contrast images of myotubes prior to transfection with Atox1 targeting siRNA (top) and immunofluorescence images 48 hours after transfection (bottom) showing no overt phenotype in myotubes transfected with siAtox1 and stained with an antibody to myosin heavy chain (MyHC). Bar = 200 µm.

Supplemental Table 1: Antibodies used in this study

Company	Antibody	Catalog #	Dilution
Custom made[59]	ATP7A	N/A	1:1000
Proteintech	ATOX1	22641-1-AP	1:1000
DSHB* hybridoma supernatant	Embryonic myosin heavy chain (eMyHC)	F1.652 clone[60]	1:10 (stain)<br>1:100 (blot)
DSHB* hybridoma supernatant	Flag	12C6c (RRID:AB_2890618)	1:1000 (blot)
Sigma Aldrich	Flag	F1804	1:1000 (stain)
Cell Signaling Technology	Histone H3	4499s	1:4000
Cell Signaling Technology	GAPDH	5174S	1:1000
Santa Cruz	CCS	sc-55561	1:1000
Cell Signaling Technology	MEK1/2	9122	1:1000
Cell Signaling Technology	ERK 1/2	4695	1:1000
Cell Signaling Technology	Phospho-ERK1/2 (T202, Y204)	4370	1:2000
DSHB* hybridoma supernatant	Pan myosin heavy chain	MF-20 clone[61]	1:1000 (stain)<br>1:2000 (blot)
DSHB* hybridoma supernatant	Myogenin	FD5 clone[62]	1:1000
Cell Signaling Technology	HSP90	4877S	1:4000
Proteintech	CRIP2	14801-1-AP	1:1000
Proteintech	SYNCRIP	14024-1-AP	1:1000
Jackson Laboratory	Mouse-HRP	115-035-003	1:10000
Jackson Laboratory	Rabbit-HRP	111-035-003	1:10000
Cell Signaling Technology	Streptavidin-HRP	3999S	1:10,000
Jackson Laboratory	Mouse-Alexa Fluor 488	715-545-151	1:500

*DSHB: Developmental Studies Hybridoma Bank

Supplemental Table 2: Complete proteomics data

708 Proteins identified

High (99%) FDR confidence, master protein, 2 peptides and no contamination

Protein FDR Confidence: Combined	Accession	Description	Gene Symbol	Coverage [%]	# Peptides	# PSMs	# Unique Peptides	# AAs	MW [kDa]	calc. pI	Entrez Gene ID	Ensembl Gene ID	Gene ID	Abundance: F3: Sample, Myoblasts (MB), PLUS Bis-Phenol (BP)	Abundance: F5: Sample, Myoblasts (MB), PLUS Bis-Phenol (BP)	Abundance: F6: Sample, Myoblasts (MB), PLUS Bis-Phenol (BP)	Abundance: F10: Sample, Myocytes (MC), PLUS Bis-Phenol (BP)	Abundance: F11: Sample, Myocytes (MC), PLUS Bis-Phenol (BP)	Abundance: F12: Sample, Myocytes (MC), PLUS Bis-Phenol (BP)	Abundance: F16: Sample, Myotubes (MT), PLUS Bis-Phenol (BP)	Abundance: F17: Sample, Myotubes (MT), PLUS Bis-Phenol (BP)	Abundance: F19: Sample, Myotubes (MT), PLUS Bis-Phenol (BP)
High	Q9QXS1-3	Isoform PLEC-1A of Plectin OS=Mus musculus OX=10090 GN=Plec	Plec	43	234	1951	1	4543	517	5.8			Q9QXS1-3		424590.8	626467.2						
High	P52480	Pyruvate kinase PKM OS=Mus musculus OX=10090 GN=Pkm PE=1 SV=4	Pkm	74	43	681	3	531	57.8	7.47	18746	ENSMUSG0	mmu:1874	4.73E+08	1.17E+08	2.53E+08	3.27E+08	1.89E+08	4.33E+08	1.78E+08	3.73E+08	94848260
High	P52480-2	Isoform M1 of Pyruvate kinase PKM OS=Mus musculus OX=10090 GN=Pkm	Pkm	66	41	661	1	531	57.9	7.14			P52480-2	1357345	841895	487220.7		504184.1			3812587	2804277
High	Q8BMK4	Cytoskeleton-associated protein 4 OS=Mus musculus OX=10090 GN=Ckap4 PE=1 SV=2	Ckap4	79	63	607	63	575	63.7	5.64	216197	ENSMUSG0	B2RRB4; m	4.54E+08	41276584	1.2E+08	98833616	77716863	1.48E+08	1.19E+08	3.39E+08	1.45E+08
High	P27773	Protein disulfide-isomerase A3 OS=Mus musculus OX=10090 GN=Pdia3 PE=1 SV=2	Pdia3	65	43	521	43	505	56.6	6.21	14827	ENSMUSG0	mmu:1482	3.18E+08	99066735	2.63E+08	2.85E+08	1.19E+08	3.55E+08	2.07E+08	6.23E+08	1.58E+08
High	P62631	Elongation factor 1-alpha 2 OS=Mus musculus OX=10090 GN=Eef1a2 PE=1 SV=1	Eef1a2	17	10	523	1	463	50.4	9.03	13628	ENSMUSG0	mmu:1362									
High	A2AQ07	Tubulin beta-1 chain OS=Mus musculus OX=10090 GN=Tubb1 PE=1 SV=1	Tubb1	11	8	461	2	451	50.4	5.07	545486	ENSMUSG0	A2AQ07; m	489580.3	1122725	2467770	1670924	1093812	3962824			
High	Q922R8	Protein disulfide-isomerase A6 OS=Mus musculus OX=10090 GN=Pdia6 PE=1 SV=3	Pdia6	49	25	242	25	440	48.1	5.14	71853		mmu:7185	80300439	15262473	56139288	65439085	13051359	90572709	47400635	1.5E+08	34933365
High	P17183	Gamma-enolase OS=Mus musculus OX=10090 GN=Eno2 PE=1 SV=2	Eno2	24	10	260	1	434	47.3	5.11	13807	ENSMUSG0	mmu:1380									
High	P17156	Heat shock-related 70 kDa protein 2 OS=Mus musculus OX=10090 GN=Hspa2 PE=1 SV=2	Hspa2	23	18	226	2	633	69.6	5.67	15512	ENSMUSG0	mmu:1551									
High	Q3U0V1	Far upstream element-binding protein 2 OS=Mus musculus OX=10090 GN=Khsrp PE=1 SV=2	Khsrp	35	24	223	20	748	76.7	7.33	16549	ENSMUSG0	E9QKH3; m	6754590	43580091	1.15E+08	65723969	49079683	73599531	62652528	37150158	6932016
High	Q6GSS7	Histone H2A type 2-A OS=Mus musculus OX=10090 GN=Hist2h2aa1 PE=1 SV=3	Hist2h2aa1	43	9	300	1	130	14.1	10.9	15267; 319	ENSMUSG0	mmu:1526		610625.8							1739259
High	P09103	Protein disulfide-isomerase OS=Mus musculus OX=10090 GN=P4hb PE=1 SV=2	P4hb	60	46	290	46	509	57	4.88	18453	ENSMUSG0	mmu:1845	1.95E+08	39860087	79521938	67243582	36337676	47285902	73236646	1.15E+08	52690173
High	Q8BGD9	Eukaryotic translation initiation factor 4B OS=Mus musculus OX=10090 GN=Eif4b PE=1 SV=1	Eif4b	27	20	201	20	611	68.8	5.67	75705	ENSMUSG0	mmu:7570	8602274	74800951	98146006	63634511	45392997	41412485	43802539	18055326	5544518
High	Q6NZJ6	Eukaryotic translation initiation factor 4 gamma 1 OS=Mus musculus OX=10090 GN=Eif4g1 PE=1 SV=1	Eif4g1	24	35	267	3	1600	176	5.4	208643	ENSMUSG0	mmu:2086	23617425	95329232	1.55E+08	79569477	41422916	74105139	60748443	1.23E+08	25118984
High	Q64524	Histone H2B type 2-E OS=Mus musculus OX=10090 GN=H2bc21 PE=1 SV=3	H2bc21	56	9	257	1	126	14	10.32	319190	ENSMUSG0	mmu:3191									
High	Q6NZJ6-2	Isoform 2 of Eukaryotic translation initiation factor 4 gamma 1 OS=Mus musculus OX=10090 GN=Eif4g1	Eif4g1	23	33	238	1	1586	174.5	5.38			Q6NZJ6-2		774370.1	1050836						
High	P09055	Integrin beta-1 OS=Mus musculus OX=10090 GN=Itgb1 PE=1 SV=1	Itgb1	19	17	143	17	798	88.2	5.94	16412	ENSMUSG0	F6R105; P0	49550712	12775353	69305363	63870506	26056609	43938713	26850456	31945391	8616779
High	P14873	Microtubule-associated protein 1B OS=Mus musculus OX=10090 GN=Map1b PE=1 SV=2	Map1b	22	43	246	42	2464	270.1	4.83	17755	ENSMUSG0	E9QM11; m	19193558	75088453	44934983	44104044	38425137	49109774	38252052	52165905	10899168
High	P07091	Protein S100-A4 OS=Mus musculus OX=10090 GN=S100a4 PE=1 SV=1	S100a4	53	8	97	8	101	11.7	5.31	20198	ENSMUSG0	mmu:2019	535379.1	3078463	4489237	20570636	2082816	11128357	20177777	99158464	37562853
High	P08003	Protein disulfide-isomerase A4 OS=Mus musculus OX=10090 GN=Pdia4 PE=1 SV=3	Pdia4	37	29	205	29	638	71.9	5.31	12304		mmu:1230	88413990	23926803	76116594	53930966	13689692	1.27E+08	54952871	68550601	11476254
High	P16627	Heat shock 70 kDa protein 1-like OS=Mus musculus OX=10090 GN=Hspa1l PE=1 SV=4	Hspa1l	17	13	146	3	641	70.6	6.24	15482	ENSMUSG0	mmu:1548	4223702	9190227	20911355	7582902	799988.5	2965406	13676275		1194395
High	Q61033	Lamina-associated polypeptide 2, isoforms alpha/zeta OS=Mus musculus OX=10090 GN=Tmpo PE=1 SV=4	Tmpo	45	27	152	15	693	75.1	8.05	21917	ENSMUSG0	B2RUB9; Q6	17099090	49004412	71645717	22291223	20706692	35858554	14550999	17023613	10469738
High	O08807	Peroxiredoxin-4 OS=Mus musculus OX=10090 GN=Prdx4 PE=1 SV=1	Prdx4	49	12	117	10	274	31	7.15	53381	ENSMUSG0	B1AZS7; m	73292328	7774893	12530713	14842105	5651022	29321408	10511981	1.06E+08	60095607
High	O08795	Glucosidase 2 subunit beta OS=Mus musculus OX=10090 GN=Prkcsh PE=1 SV=1	Prkcsh	23	16	122	16	521	58.8	4.46	19089	ENSMUSG0	mmu:1908	1.19E+08	35705947	72759387	36252647	16215881	66959461	39875328	1.36E+08	43083852
High	Q71LX4	Talin-2 OS=Mus musculus OX=10090 GN=Tln2 PE=1 SV=3	Tln2	7	18	135	3	2375	253.5	5.8	70549		E9QM49; m		709304.4	575699.2	1834871		1928928	1411307		
High	P17879	Heat shock 70 kDa protein 1B OS=Mus musculus OX=10090 GN=Hspa1b PE=1 SV=3	Hspa1b	24	16	130	6	642	70.1	5.72	15511	ENSMUSG0	mmu:1551	3369079	2683509	8088023	1243717	1116580	2122275	978916.3	7186050	1392583
High	Q6IRU2	Tropomyosin alpha-4 chain OS=Mus musculus OX=10090 GN=Tpm4 PE=1 SV=3	Tpm4	39	13	105	7	248	28.5	4.68	326618	ENSMUSG0	mmu:3266	3465800	9950801	29578236	23275751	6759959	14280417	17206614	50864908	18148072
High	Q60749	KH domain-containing, RNA-binding, signal transduction-associated protein 1 OS=Mus musculus OX=10090 GN=Khdrbs1 PE=1 SV=2	Khdrbs1	26	14	121	14	443	48.3	8.72	20218	ENSMUSG0	A2ACH3; B2	4420041	49582722	74472752	45694195	67912997	66135520	46094596	13640667	3338299
High	Q62095	ATP-dependent RNA helicase DDX3Y OS=Mus musculus OX=10090 GN=Ddx3y PE=1 SV=2	Ddx3y	36	23	113	1	658	73.4	7.53	26900	ENSMUSG0	mmu:2690									
High	Q3UZ39	Leucine-rich repeat flightless-interacting protein 1 OS=Mus musculus OX=10090 GN=Lrrfip1 PE=1 SV=2	Lrrfip1	30	19	80	12	729	79.2	4.82	16978	ENSMUSG0	mmu:1697	1878846	22874922	38908489	5057999	17340423	5623832	2780387	1508197	1006187
High	P51125	Calpastatin OS=Mus musculus OX=10090 GN=Cast PE=1 SV=2	Cast	24	16	75	16	788	84.9	5.52	12380	ENSMUSG0	mmu:1238	964427.8	8873974	13150871	31422944	22069447	5066363	18797910	3647285	405169.5
High	Q9DCL9	Bifunctional phosphoribosylaminoimidazole carboxylase/phosphoribosylaminoimidazole succinocarboxamide synthetase OS=Mus muscu	Paics	22	11	89	11	425	47	7.23	67054	ENSMUSG0	mmu:6705	9643757	25756419	44049466	23767936	7237222	37926485	12979021	39667865	11711338
High	Q60865	Caprin-1 OS=Mus musculus OX=10090 GN=Caprin1 PE=1 SV=2	Caprin1	21	17	132	17	707	78.1	5.25	53872	ENSMUSG0	mmu:5387	6605959	30306866	1.06E+08	38436618	26856373	36755262	12764997	13295061	9479597
High	Q61029	Lamina-associated polypeptide 2, isoforms beta/delta/epsilon/gamma OS=Mus musculus OX=10090 GN=Tmpo PE=1 SV=4	Tmpo	35	15	86	3	452	50.3	9.45	21917	ENSMUSG0	mmu:2191	1415111	1090934	3772220	1158678		1076370	1532729		
High	Q9WU78	Programmed cell death 6-interacting protein OS=Mus musculus OX=10090 GN=Pdcd6ip PE=1 SV=3	Pdcd6ip	29	25	140	25	869	96	6.52	18571	ENSMUSG0	mmu:1857	14210431	1646469	35255895	16358853	3036596	33055516	7831076	71537050	24138616
High	Q9Z1Q9	Valine--tRNA ligase OS=Mus musculus OX=10090 GN=Vars1 PE=1 SV=1	Vars1	13	13	59	13	1263	140.1	7.77	22321	ENSMUSG0	mmu:2232	11045845	4655607	20193099	17427422	5851890	11184299	3817824	7338798	2130691
High	Q8CAQ8	MICOS complex subunit Mic60 OS=Mus musculus OX=10090 GN=Immt PE=1 SV=1	Immt	24	18	100	2	757	83.8	6.61	76614	ENSMUSG0	mmu:7661	21403869	13735973	44468285	16819324	9629885	30268394	19618316	33787111	17413574
High	Q91YQ5	Dolichyl-diphosphooligosaccharide--protein glycosyltransferase subunit 1 OS=Mus musculus OX=10090 GN=Rpn1 PE=1 SV=1	Rpn1	29	17	61	17	608	68.5	6.46	103963	ENSMUSG0	mmu:1039	18287669	3617459	7581171	5261102	2595246	13297420	13135083	10012863	3575631
High	P35564	Calnexin OS=Mus musculus OX=10090 GN=Canx PE=1 SV=1	Canx	21	14	67	14	591	67.2	4.64	12330	ENSMUSG0	mmu:1233	17091571	7192360	17100260	391693	366151.5	5694196	6647052	15588919	16957746
High	Q8CAQ8-3	Isoform 3 of MICOS complex subunit Mic60 OS=Mus musculus OX=10090 GN=Immt	Immt	24	17	98	1	709	78.7	7.25			Q8CAQ8-3	416563.9	305073.8	1484284	755395.4	365408.3	838010.8			492782.1
High	P97379	Ras GTPase-activating protein-binding protein 2 OS=Mus musculus OX=10090 GN=G3bp2 PE=1 SV=2	G3bp2	12	6	61	5	482	54.1	5.62	23881	ENSMUSG0	mmu:2388	1241274	13436811	17907050	9834244	12686780	8080286	9024964	2701440	2036815
High	Q8BHN3	Neutral alpha-glucosidase AB OS=Mus musculus OX=10090 GN=Ganab PE=1 SV=1	Ganab	9	7	63	7	944	106.8	6.06	14376	ENSMUSG0	mmu:1437	13783818	2059666	14843793	15664627	4580945	19941326	8065057	21566967	1890109
High	Q9JKS4	LIM domain-binding protein 3 OS=Mus musculus OX=10090 GN=Ldb3 PE=1 SV=1	Ldb3	15	9	47	2	723	76.4	7.75	24131	ENSMUSG0	B2RSB0; B7				9515903	1461396	3715699	6452005		
High	Q91ZX7	Prolow-density lipoprotein receptor-related protein 1 OS=Mus musculus OX=10090 GN=Lrp1 PE=1 SV=1	Lrp1	4	19	70	19	4545	504.4	5.36	16971		mmu:1697	15453187	11641492	21547914	19195983	13159140	13942992	18646309	31876796	17575030
High	P68510	14-3-3 protein eta OS=Mus musculus OX=10090 GN=Ywhah PE=1 SV=2	Ywhah	17	7	51	2	246	28.2	4.89	22629	ENSMUSG0	mmu:2262	898759.2	11817955	13429936	18806169	9984165	11574056	8937091	12969722	2368034
High	Q8BWW4	La-related protein 4 OS=Mus musculus OX=10090 GN=Larp4 PE=1 SV=2	Larp4	25	18	85	18	719	79.7	6.39	207214		mmu:2072	9323227	14725033	73534442	13383816	15553716	18343710	5439719	14013027	3459675
High	Q3UMC0	Ribosome biogenesis protein SPATA5 OS=Mus musculus OX=10090 GN=Spata5 PE=1 SV=2	Afg2a	5	3	41	2	893	97.2	8.24	57815	ENSMUSG0	A2ACU2; A3									
High	P22437	Prostaglandin G/H synthase 1 OS=Mus musculus OX=10090 GN=Ptgs1 PE=1 SV=1	Ptgs1	25	17	72	16	602	69	6.83	19224	ENSMUSG0	mmu:1922	40186194	5862836	11291144	4072113	2676840	18307051	3960154	23174154	4708623
High	Q8K298	Anillin OS=Mus musculus OX=10090 GN=Anln PE=1 SV=2	Anln	9	9	42	9	1121	122.7	6.98	68743	ENSMUSG0	mmu:6874	10183530	9572276	26827901	7579267	3222055	4670577	2386581	7772653	1347383
High	P57776	Elongation factor 1-delta OS=Mus musculus OX=10090 GN=Eef1d PE=1 SV=3	Eef1d	43	12	49	12	281	31.3	5.02	66656		mmu:6665	2397696	4685770	15121204	9022747	1631691	11572547	3989632	26791661	39411146
High	Q9JKS4-2	Isoform 2 of LIM domain-binding protein 3 OS=Mus musculus OX=10090 GN=Ldb3	Ldb3	18	10	43	3	684	72.7	7.78			Q9JKS4-2		1437829	1880663	48960885	4642858	3653648	84721411	4226581	992095.1
High	P63101	14-3-3 protein zeta/delta OS=Mus musculus OX=10090 GN=Ywhaz PE=1 SV=1	Ywhaz	23	7	44	3	245	27.8	4.79	22631	ENSMUSG0	mmu:2263	599518.9	1551226	2691010	1692661	532441.2	2105737	1544269	12968311	6611879
High	Q9R0B9	Procollagen-lysine,2-oxoglutarate 5-dioxygenase 2 OS=Mus musculus OX=10090 GN=Plod2 PE=1 SV=2	Plod2	12	9	49	9	737	84.4	6.89	26432	ENSMUSG0	E9QM54; m	22914797	1419042	13210958	4516341	512649.7	13809353	4404643	28227456	9381388
High	Q8CG48	Structural maintenance of chromosomes protein 2 OS=Mus musculus OX=10090 GN=Smc2 PE=1 SV=2	Smc2	23	28	68	28	1191	134.2	8.41	14211	ENSMUSG0	mmu:1421	7267628	4919399	39676262	9626500	5111658	7308359	2702744	4690454	1411081
High	Q91VX2	Ubiquitin-associated protein 2 OS=Mus musculus OX=10090 GN=Ubap2 PE=1 SV=1	Ubap2	15	13	59	13	1132	117.9	7.72	68926	ENSMUSG0	mmu:6892	2241888	13603174	29946414	27736102	11383104	14509305	13641980	33992588	15527293
High	Q9CT10	Ran-binding protein 3 OS=Mus musculus OX=10090 GN=Ranbp3 PE=1 SV=2	Ranbp3	7	2	36	2	491	52.5	5.12	71810	ENSMUSG0	A6H6I9; m	337396.3	9573833	11684779	7846773	7003727	6602669	8354895	4315633	
High	Q91WJ8	Far upstream element-binding protein 1 OS=Mus musculus OX=10090 GN=Fubp1 PE=1 SV=1	Fubp1	12	9	45	5	651	68.5	7.93		ENSMUSG0	Q8C0Y8; Q9	266222.7	1196996	4116212	1609263	668196.5	2006414	1654521	1496762	1415347
High	P17751	Triosephosphate isomerase OS=Mus musculus OX=10090 GN=Tpi1 PE=1 SV=5	Tpi1	37	9	50	9	249	26.7	7.3	21991	ENSMUSG0	mmu:2199	3173157	3887283	8520482	16718037	2338744	12403856	12017394	46769431	35913517
High	Q8BI84	Transport and Golgi organization protein 1 homolog OS=Mus musculus OX=10090 GN=Mia3 PE=1 SV=2	Mia3	12	19	77	19	1930	213.5	4.75	338366		A0JLX8; m	14668184	17560036	13341162	7531324	7356722	6548175	6297810	16080506	3694923
High	Q3V3R1	Monofunctional C1-tetrahydrofolate synthase, mitochondrial OS=Mus musculus OX=10090 GN=Mthfd1l PE=1 SV=2	Mthfd1l	13	12	48	11	977	105.7	7.02	270685	ENSMUSG0	mmu:2706	3648570		12681953	2905875	1238943	9721004	2672605	6653260	5988658
High	P08121	Collagen alpha-1(III) chain OS=Mus musculus OX=10090 GN=Col3a1 PE=1 SV=4	Col3a1	12	13	56	11	1464	138.9	6.52	12825	ENSMUSG0	mmu:1282	12254553	12826720	18187374	15844777	21878234	32660689	27346698	15757968	2341154
High	Q921T2	Torsin-1A-interacting protein 1 OS=Mus musculus OX=10090 GN=Tor1aip1 PE=1 SV=3	Tor1aip1	10	5	29	5	595	66.7	7.05	208263	ENSMUSG0	E9QLK1; m	6891755	4799212	5516606	14874118	11600766	4153947	21373008	1526148	1487277
High	P05202	Aspartate aminotransferase, mitochondrial OS=Mus musculus OX=10090 GN=Got2 PE=1 SV=1	Got2	30	12	29	12	430	47.4	9	14719	ENSMUSG0	mmu:1471	658787.4		477978.3						45027723
High	A2AGT5	Cytoskeleton-associated protein 5 OS=Mus musculus OX=10090 GN=Ckap5 PE=1 SV=1	Ckap5	9	16	55	16	2032	225.5	7.96	75786		A0PJ77; A2	1404720	9215126	19161547	7167857	4673750	7615193	2618627	6196504	58947.01
High	Q3UPL0	Protein transport protein Sec31A OS=Mus musculus OX=10090 GN=Sec31a PE=1 SV=2	Sec31a	10	12	58	12	1230	133.5	6.76	69162	ENSMUSG0	mmu:6916	798141.1	2155604	13016232	13689391	4537799	8443533	9577006	7469606	4549386
High	Q8BQ30	Phostensin OS=Mus musculus OX=10090 GN=Ppp1r18 PE=1 SV=1	Ppp1r18	16	9	53	9	594	65.6	5.39	76448	ENSMUSG0	B8JJ63; B8	2871874	8791286	18085255	8537601	9713038	6641310	5303116		488223.1
High	P57759	Endoplasmic reticulum resident protein 29 OS=Mus musculus OX=10090 GN=Erp29 PE=1 SV=2	Erp29	22	6	34	6	262	28.8	6.15	67397	ENSMUSG0	mmu:6739	10004807	3353693	7404769	6682537	1734812	9855910	5089276	16611916	4072173
High	Q61881	DNA replication licensing factor MCM7 OS=Mus musculus OX=10090 GN=Mcm7 PE=1 SV=1	Mcm7	24	16	46	16	719	81.2	6.37	17220	ENSMUSG0	mmu:1722	7657070	957646.3	21055542	1279761	1368339	8569628	600864.8	3861938	1455287
High	Q7M6Y3-5	Isoform 5 of Phosphatidylinositol-binding clathrin assembly protein OS=Mus musculus OX=10090 GN=Picalm	Picalm	16	7	46	7	655	70.9	8.37			Q7M6Y3-5	3180772	4183363	10053730	5082327	3809340	9317452	4224098	6075104	1890809
High	Q8CH18	Cell division cycle and apoptosis regulator protein 1 OS=Mus musculus OX=10090 GN=Ccar1 PE=1 SV=1	Ccar1	6	7	39	7	1146	132	5.76	67500	ENSMUSG0	mmu:6750	1753787	9653984	34220839	14934620	11140526	18521142	8438091	7670003	1026071
High	Q61171	Peroxiredoxin-2 OS=Mus musculus OX=10090 GN=Prdx2 PE=1 SV=3	Prdx2	46	9	52	9	198	21.8	5.41	21672	ENSMUSG0	mmu:2167	4366830	5949752	13365329	23683343	7421472	29174191	20741520	99507780	52997410
High	G5E870	E3 ubiquitin-protein ligase TRIP12 OS=Mus musculus OX=10090 GN=Trip12 PE=1 SV=1	Trip12	6	11	51	11	2025	224	8.35	14897	ENSMUSG0	G5E870; m	1675175	8783690	15172468	3726774	6153546	8819434	2408861	4350976	923702
High	P16110	Galectin-3 OS=Mus musculus OX=10090 GN=Lgals3 PE=1 SV=3	Lgals3	24	7	39	7	264	27.5	8.38			P16110	1176145	1848281	9417330	20208292	1509933	14280983	17574787	19725646	5854612
High	P24452	Macrophage-capping protein OS=Mus musculus OX=10090 GN=Capg PE=1 SV=2	Capg	8	3	31	3	352	39.2	7.2			P24452		2211777	3578192	9828536	2202451	8206023	9105168	3890363	972720.3
High	E9PVX6	Proliferation marker protein Ki-67 OS=Mus musculus OX=10090 GN=Mki67 PE=1 SV=1	Mki67	8	19	47	19	3177	350.7	9.72	17345	ENSMUSG0	E9PVX6; m	5485990	15254707	23374998	11214698	5136612	22759756	10881165	7103078	1120493
High	Q8R1X6	Spartin OS=Mus musculus OX=10090 GN=Spart PE=1 SV=1	Spart	10	4	28	4	671	72.6	5.86	229285	ENSMUSG0	mmu:2292	960953.3	3712569	8613773	4953127	2876800	5011358	2954759		884163.3
High	Q8CHH9	Septin-8 OS=Mus musculus OX=10090 GN=Septin8 PE=1 SV=4	Septin8	7	5	43	3	429	49.8	6	20362	ENSMUSG0	B1AQY7; m		8704846	6628873	5417117	7372540	5522997	11026304		1243906
High	Q61595	Kinectin OS=Mus musculus OX=10090 GN=Ktn1 PE=1 SV=1	Ktn1	10	14	45	14	1327	152.5	5.86	16709		mmu:1670	12064819	6531125	28130480	4541500	5332223	14562140	9801555	14049519	6942258
High	D3YXK2	Scaffold attachment factor B1 OS=Mus musculus OX=10090 GN=Safb PE=1 SV=2	Safb	11	10	39	7	937	105	5.35	224903	ENSMUSG0	D3YXK2; m	613753.4	7212029	20951436	9095683	6596398	9470319	6706809	8499887	3133438
High	P62137	Serine/threonine-protein phosphatase PP1-alpha catalytic subunit OS=Mus musculus OX=10090 GN=Ppp1ca PE=1 SV=1	Ppp1ca	16	4	24	4	330	37.5	6.33	19045	ENSMUSG0	mmu:1904	4455868	1496973	3986717	2474152	520463.4	12725055	1351554	20457911	5751881
High	Q3THS6	S-adenosylmethionine synthase isoform type-2 OS=Mus musculus OX=10090 GN=Mat2a PE=1 SV=2	Mat2a	20	8	36	8	395	43.7	6.48	232087	ENSMUSG0	mmu:2320	3108784	3917922	7781673	4734960	973186.6	10424941	2160322	7244570	1874803
High	Q8C052	Microtubule-associated protein 1S OS=Mus musculus OX=10090 GN=Map1s PE=1 SV=2	Map1s	11	11	51	11	973	102.9	7.02	270058	ENSMUSG0	E9QKR8; m	2422519	1687515	16294040	20211706	5901918	11312294	11145563	10879651	5115386
High	E9Q0S6	Tensin-1 OS=Mus musculus OX=10090 GN=Tns1 PE=1 SV=1	Tns1	10	14	62	13	1888	201.3	7.75	21961	ENSMUSG0	E9Q0S6; m	1506428	11150194	9910925	28189706	13968287	4011818	10573071	6276618	4756306
High	Q9DBR1	5'-3' exoribonuclease 2 OS=Mus musculus OX=10090 GN=Xrn2 PE=1 SV=1	Xrn2	6	4	21	4	951	108.6	7.59	24128	ENSMUSG0	mmu:2412	1618031	2309709	9277974	5889091	5896313	3064706			

High	Q2KN98	Cytospin-A OS=Mus musculus OX=10090 GN=Specc1l PE=1 SV=1	Specc1l	7	7	34	7	1118	124.4	5.76	74392	ENSMUSG0	mmu:7439	939243.7	10999713	14713072	8355256	9121738	6042277	8039481	2363172	592161.9
High	Q9DBZ1-2	Isoform 2 of Inhibitor of nuclear factor kappa-B kinase-interacting protein OS=Mus musculus OX=10090 GN=Ikbip	Ikbip	12	4	26	2	345	38.5	8.66			Q9DBZ1-2	1427093	452019.8	741862.9	1923955	966136.9	2126113	4389894	7393577	1657285
High	Q61166	Microtubule-associated protein RP/EB family member 1 OS=Mus musculus OX=10090 GN=Mapre1 PE=1 SV=3	Mapre1	29	5	29	5	268	30	5.22	13589	ENSMUSG0	mmu:1358			2431303	12430335	699394.6	12563350	12233217	8326532	2393524
High	Q05769	Prostaglandin G/H synthase 2 OS=Mus musculus OX=10090 GN=Ptgs2 PE=1 SV=1	Ptgs2	24	14	40	13	604	69	7.37	19225	ENSMUSG0	mmu:1922	33751811	789759.5	8110602		351056.4	4476937	7027015	8051184	5031753
High	O88543	COP9 signalosome complex subunit 3 OS=Mus musculus OX=10090 GN=Cops3 PE=1 SV=3	Cops3	5	2	17	2	423	47.8	6.65	26572	ENSMUSG0	mmu:2657	239797.2	206924	829487.4	2051345	370249.7	4233210	651854.3	8346861	4228744
High	Q60715	Prolyl 4-hydroxylase subunit alpha-1 OS=Mus musculus OX=10090 GN=P4ha1 PE=1 SV=2	P4ha1	25	12	48	1	534	60.9	5.9	18451	ENSMUSG0	mmu:1845	757031.1	173298.5	473997				1231401	2526904	1241525
High	Q3UZ39-2	Isoform 2 of Leucine-rich repeat flightless-interacting protein 1 OS=Mus musculus OX=10090 GN=Lrrfip1	Lrrfip1	14	9	33	2	628	71.3	6.04			Q3UZ39-2									
High	P08122	Collagen alpha-2(IV) chain OS=Mus musculus OX=10090 GN=Col4a2 PE=1 SV=4	Col4a2	9	11	41	11	1707	167.2	8.48	12827	ENSMUSG0	mmu:1282	23667557	2005197	9100169	6815324	644235.4	6394830	2853742	7008033	1612052
High	Q99LJ0	CTTNBP2 N-terminal-like protein OS=Mus musculus OX=10090 GN=Cttnbp2nl PE=1 SV=1	Cttnbp2nl	17	7	21	7	638	69.8	7.71	80281	ENSMUSG0	mmu:8028	672443.3	8240272	14256701	4608295	5905669	3814532	3182344		
High	Q99LE6	ATP-binding cassette sub-family F member 2 OS=Mus musculus OX=10090 GN=Abcf2 PE=1 SV=1	Abcf2	11	7	27	7	628	71.7	7.05	27407	ENSMUSG0	mmu:2740	5750764	6177880	5287831	2967761	1261597	4517436	2360373	12777733	6433754
High	Q60715-2	Isoform 2 of Prolyl 4-hydroxylase subunit alpha-1 OS=Mus musculus OX=10090 GN=P4ha1	P4ha1	25	13	53	2	534	60.8	5.81			Q60715-2	18264792	3527415	5260733	4290189	3910177	8927916	5607593	33423740	12191020
High	Q8VDW0	ATP-dependent RNA helicase DDX39A OS=Mus musculus OX=10090 GN=Ddx39a PE=1 SV=1	Ddx39a	19	8	61	1	427	49	5.68	68278	ENSMUSG0	mmu:6827	1674152		666958.3		565236.9		2422557		1141483
High	P97855	Ras GTPase-activating protein-binding protein 1 OS=Mus musculus OX=10090 GN=G3bp1 PE=1 SV=1	G3bp1	26	9	58	8	465	51.8	5.59	27041	ENSMUSG0	mmu:2704	1723714	17243485	34552253	13660725	11628505	11616972	11213720	10251030	4136021
High	P24547	Inosine-5'-monophosphate dehydrogenase 2 OS=Mus musculus OX=10090 GN=Impdh2 PE=1 SV=2	Impdh2	8	4	18	4	514	55.8	7.28	23918	ENSMUSG0	mmu:2391	2617879	2034041	9125910		405163.9	5770462		11261767	5765969
High	Q9EP71	Ankycorbin OS=Mus musculus OX=10090 GN=Rai14 PE=1 SV=1	Rai14	15	13	29	13	979	108.8	6.27	75646	ENSMUSG0	mmu:7564	6174198	2915511	13588336	3726401	2360886	4590357	3805489	17659826	5706298
High	O35609	Secretory carrier-associated membrane protein 3 OS=Mus musculus OX=10090 GN=Scamp3 PE=1 SV=3	Scamp3	10	2	20	2	349	38.4	7.64	24045	ENSMUSG0	mmu:2404	395871.3	1786471	2664770	8343835	2519945	1246751	8699962		
High	Q9DBZ1	Inhibitor of nuclear factor kappa-B kinase-interacting protein OS=Mus musculus OX=10090 GN=Ikbip PE=1 SV=2	Ikbip	9	3	25	1	373	42.5	5.1	67454	ENSMUSG0	mmu:6745									
High	Q9Z1N5	Spliceosome RNA helicase Ddx39b OS=Mus musculus OX=10090 GN=Ddx39b PE=1 SV=1	Ddx39b	19	8	61	1	428	49	5.67	53817	ENSMUSG0	mmu:5381	11515399	3694709	9405180	9542618	2121725	28815466	4856532	43492588	12502662
High	O55131	Septin-7 OS=Mus musculus OX=10090 GN=Septin7 PE=1 SV=1	Septin7	31	14	61	13	436	50.5	8.57	235072		mmu:2350	3184348	6150787	11565684	12476748	6013353	15544374	12947715	25019780	16978958
High	Q60716	Prolyl 4-hydroxylase subunit alpha-2 OS=Mus musculus OX=10090 GN=P4ha2 PE=1 SV=1	P4ha2	25	12	53	11	537	61	5.8	18452	ENSMUSG0	mmu:1845	18209015	3861601	7614129		5401455	5568207	1231527	2850437	4265650
High	Q6A065	Centrosomal protein of 170 kDa OS=Mus musculus OX=10090 GN=Cep170 PE=1 SV=2	Cep170	12	20	53	19	1588	174.9	7.17	545389	ENSMUSG0	Q6A065; Q	1521993	11043861	21193090	1.03E+08	9015155	50214759	92557474	17389204	846611.9
High	Q8CCS6	Polyadenylate-binding protein 2 OS=Mus musculus OX=10090 GN=Pabpn1 PE=1 SV=3	Pabpn1	8	2	13	2	302	32.3	5.17	54196	ENSMUSG0	mmu:5419		1188221	1770975		1126601	1464853			566057.9
High	Q80XI3	Eukaryotic translation initiation factor 4 gamma 3 OS=Mus musculus OX=10090 GN=Eif4g3 PE=1 SV=2	Eif4g3	4	7	31	6	1579	174.8	5.53	230861		mmu:2308	2174689	5450692	9939667	2699757	4144956	930131.6	4228129	2293429	
High	Q62418-2	Isoform 2 of Drebrin-like protein OS=Mus musculus OX=10090 GN=Dbnl	Dbnl	12	5	31	5	433	48.4	4.92			Q62418-2		3584153	3004047	5639491	3985269	1827004	5970753	3415296	
High	Q3UDE2	Tubulin--tyrosine ligase-like protein 12 OS=Mus musculus OX=10090 GN=Ttll12 PE=1 SV=1	Ttll12	12	6	18	6	639	74	5.63	223723	ENSMUSG0	mmu:2237	1246750	1523635	5026932	1797055	674581.3	1712444	1970143	2300704	
High	Q61191	Host cell factor 1 OS=Mus musculus OX=10090 GN=Hcfc1 PE=1 SV=2	Hcfc1	9	15	48	15	2045	210.3	7.18	15161	ENSMUSG0	B1AUX1; m	3172013	9624931	20562940	10686160	4990251	5895158	12075583	16639437	7800590
High	Q3U7R1	Extended synaptotagmin-1 OS=Mus musculus OX=10090 GN=Esyt1 PE=1 SV=2	Esyt1	9	9	25	9	1092	121.5	5.95	23943	ENSMUSG0	mmu:2394	1564980	1846736	1676572	4864528	1934420	5317769	1795032	4116985	2408485
High	Q9DCT8	Cysteine-rich protein 2 OS=Mus musculus OX=10090 GN=Crip2 PE=1 SV=1	Crip2	39	4	51	4	208	22.7	8.63	68337	ENSMUSG0	mmu:6833	642040.3	17458925	13717253	41730960	8284663	15684032	51947985	20490255	11338991
High	Q8BJY1	26S proteasome non-ATPase regulatory subunit 5 OS=Mus musculus OX=10090 GN=Psmd5 PE=1 SV=4	Psmd5	11	6	22	6	504	55.9	5.21	66998	ENSMUSG0	A2AUB0; m	1818718	2758065	6557851	3539339	1837459	5975486	3597516	8601167	5509724
High	P56959	RNA-binding protein FUS OS=Mus musculus OX=10090 GN=Fus PE=1 SV=1	Fus	12	6	24	6	518	52.6	9.36	233908	ENSMUSG0	mmu:2339	15582107	815310.4	9478715	15074973	6947848	35207481	11418590	92397368	60701588
High	Q9QYR6	Microtubule-associated protein 1A OS=Mus musculus OX=10090 GN=Map1a PE=1 SV=2	Map1a	5	10	29	9	2776	300	5	17754	ENSMUSG0	A2ARN9; m	1157879	4396682	2902220	2508609	1622444	2183738	2640289	3602012	316791.2
High	P60229	Eukaryotic translation initiation factor 3 subunit E OS=Mus musculus OX=10090 GN=Eif3e PE=1 SV=1	Eif3e	22	9	30	9	445	52.2	6.04	16341	ENSMUSG0	mmu:1634	3732458	5406435	5564803	966907.8		3186332		9526711	3518726
High	Q8BFZ9	Erlin-2 OS=Mus musculus OX=10090 GN=Erlin2 PE=1 SV=1	Erlin2	24	10	26	6	340	37.8	5.5	244373	ENSMUSG0	mmu:2443	35299990	1767032	10079761	3022000	1149428	6932964	2013416	15944074	6541646
High	P97310	DNA replication licensing factor MCM2 OS=Mus musculus OX=10090 GN=Mcm2 PE=1 SV=3	Mcm2	11	10	24	10	904	102	5.72	17216	ENSMUSG0	mmu:1721	4630513	3607044	15702968	2797789	5420923	10858967		6706418	1396686
High	P47809	Dual specificity mitogen-activated protein kinase kinase 4 OS=Mus musculus OX=10090 GN=Map2k4 PE=1 SV=2	Map2k4	7	3	21	3	397	44.1	8.07	26398	ENSMUSG0	mmu:2639	654942.6	3800469	4738298	2340755	1771405	2279370	3579276		562558.8
High	P25206	DNA replication licensing factor MCM3 OS=Mus musculus OX=10090 GN=Mcm3 PE=1 SV=2	Mcm3	12	9	25	9	812	91.5	5.55	17215	ENSMUSG0	mmu:1721	3287842	1429601	7897091	1416545	1168148	4763406		2001400	268119.3
High	Q6PAM1	Alpha-taxilin OS=Mus musculus OX=10090 GN=Txlna PE=1 SV=1	Txlna	14	5	14	5	554	62.3	6.74	109658	ENSMUSG0	mmu:1096	384451.1	5413135	5659168		3001242	1225916	913847	4128061	
High	P50247	Adenosylhomocysteinase OS=Mus musculus OX=10090 GN=Ahcy PE=1 SV=3	Ahcy	7	4	12	4	432	47.7	6.54	11615; 269	ENSMUSG0	mmu:2693	2819996	577343.3	1622499	2647688	831732.5	2438429	1017491		
High	Q61576	Peptidyl-prolyl cis-trans isomerase FKBP10 OS=Mus musculus OX=10090 GN=Fkbp10 PE=1 SV=2	Fkbp10	31	17	56	17	581	64.7	5.64	14230	ENSMUSG0	A2A4I0; m	18102587	2240623	12361654	12178583	1769312	12602626	8812787	16710934	5719367
High	P60122	RuvB-like 1 OS=Mus musculus OX=10090 GN=Ruvbl1 PE=1 SV=1	Ruvbl1	16	7	37	7	456	50.2	6.42	56505	ENSMUSG0	mmu:5650	2315517	7326968	12394344	8951794	5253051	6963689	10894899	15034065	5995802
High	Q7TMK9-2	Isoform 2 of Heterogeneous nuclear ribonucleoprotein Q OS=Mus musculus OX=10090 GN=Syncrip	Syncrip	14	7	26	7	562	62.6	7.56			Q7TMK9-2	303074.6	3019112	9046358	5752529	3231236	4482188	8959507	8904761	3517210
High	Q6R0H7	Guanine nucleotide-binding protein G(s) subunit alpha isoforms XLas OS=Mus musculus OX=10090 GN=Gnas PE=1 SV=1	Gnas	7	6	47	5	1133	121.4	4.81	14683	ENSMUSG0	A2A607; A2	6723360	3881630	6496822	5905948	1803998	11089217	2505616	21978675	7820470
High	Q9DBT5	AMP deaminase 2 OS=Mus musculus OX=10090 GN=Ampd2 PE=1 SV=1		4	3	21	3	798	92	6.23				1051535	7616481	12253536	10032522	2761753	4476678	8961520	3403083	2145957
High	Q61749	Translation initiation factor eIF-2B subunit delta OS=Mus musculus OX=10090 GN=Eif2b4 PE=1 SV=2	Eif2b4	16	6	24	6	524	57.6	9.25	13667	ENSMUSG0	mmu:1366	3470791	5857610	9812090	1349607	4181199	6038548	4539814	18815431	5720853
High	P02469	Laminin subunit beta-1 OS=Mus musculus OX=10090 GN=Lamb1 PE=1 SV=3	Lamb1	8	13	30	13	1786	197	4.94	16777	ENSMUSG0	E9PXZ9; P0	16548111	760931.8	3719250	1466309	1177445	2810321	1595578	8817603	2874405
High	Q9D024	PAT complex subunit CCDC47 OS=Mus musculus OX=10090 GN=Ccdc47 PE=1 SV=2	Ccdc47	10	6	26	6	483	55.8	4.84	67163	ENSMUSG0	B1AR94; m	6031664	9885504	13890780	4689929	8053111	7445018	11006480	11773966	3994578
High	Q7TPM1	Protein PRRC2B OS=Mus musculus OX=10090 GN=Prrc2b PE=1 SV=1	Prrc2b	14	16	46	13	1486	160.8	8.16	227723	ENSMUSG0	A2AN31; m	10567304	9378617	15109514	9443975	5308029	9816588	9336598	36128451	10933110
High	Q921Q7	Ras and Rab interactor 1 OS=Mus musculus OX=10090 GN=Rin1 PE=1 SV=1	Rin1	13	8	37	8	763	83	8.72	225870	ENSMUSG0	mmu:2258	1369438	2400848	7525848	14620246	4521908	8911957	5053687	10009827	3647918
High	Q9Z0U1	Tight junction protein ZO-2 OS=Mus musculus OX=10090 GN=Tjp2 PE=1 SV=2	Tjp2	7	6	18	6	1167	131.2	6.79	21873	ENSMUSG0	mmu:2187	446730	2681708	9889631		2025194	2454001	1500427	4014759	328297.6
High	Q1HFZ0	RNA cytosine C(5)-methyltransferase NSUN2 OS=Mus musculus OX=10090 GN=Nsun2 PE=1 SV=2	Nsun2	13	9	20	9	757	85.4	6.58	28114	ENSMUSG0	A0PJD6; m	2732799	334620.5	8518196		381001.4	3315259	4039924	1261873	
High	Q8BI72	CDKN2A-interacting protein OS=Mus musculus OX=10090 GN=Cdkn2aip PE=1 SV=1	Cdkn2aip	12	5	19	5	563	59.7	9.16	70925	ENSMUSG0	mmu:7092	464822.6	3511043	6132288	3495376	2302553	2260542	2235471	2268722	
High	Q99MN1	Lysine--tRNA ligase OS=Mus musculus OX=10090 GN=Kars1 PE=1 SV=1	Kars1	15	10	28	10	595	67.8	5.94	85305	ENSMUSG0	mmu:8530	2227470	5816012	8842229	4221529	2833463	2352032	7067067	9986237	8791263
High	P26516	26S proteasome non-ATPase regulatory subunit 7 OS=Mus musculus OX=10090 GN=Psmd7 PE=1 SV=2	Psmd7	13	4	26	4	321	36.5	6.77	17463	ENSMUSG0	mmu:1746	1569762	1129680	4481451	2704870	1866394	7261956	2059121	16967106	7314534
High	O70194	Eukaryotic translation initiation factor 3 subunit D OS=Mus musculus OX=10090 GN=Eif3d PE=1 SV=2	Eif3d	18	9	31	9	548	63.9	6.05	55944	ENSMUSG0	mmu:5594	13453328	5724424	8500419	1868704	1812482	8119050	5917576	11625272	2684594
High	Q68FL6	Methionine--tRNA ligase, cytoplasmic OS=Mus musculus OX=10090 GN=Mars1 PE=1 SV=1	Mars1	11	10	24	10	902	101.4	7.14	216443	ENSMUSG0	mmu:2164	7647604	1442693	10927260	4365439		5492265	1786172	5272543	2125211
High	Q8BH64	EH domain-containing protein 2 OS=Mus musculus OX=10090 GN=Ehd2 PE=1 SV=1	Ehd2	10	6	23	5	543	61.1	6.51	259300	ENSMUSG0	mmu:2593	662986.1	713614.3	535293.4		1228719	3309993		3756839	1387459
High	Q7TSG2	RNA polymerase II subunit A C-terminal domain phosphatase OS=Mus musculus OX=10090 GN=Ctdp1 PE=1 SV=1	Ctdp1	9	6	19	6	960	104.5	5.39	67655	ENSMUSG0	mmu:6765	199329.2	5540216	8573971	1449770	4697381	1818379	2660227	1504495	
High	O08788	Dynactin subunit 1 OS=Mus musculus OX=10090 GN=Dctn1 PE=1 SV=3	Dctn1	5	6	20	6	1281	141.6	5.9	13191	ENSMUSG0	E9QLJ1; m	1467504	582433.2	6547453	1808901	1351199	4594906	3527999	6614345	3351289
High	P62717	60S ribosomal protein L18a OS=Mus musculus OX=10090 GN=Rpl18a PE=1 SV=1	Rpl18a	18	3	19	3	176	20.7	10.71	76808	ENSMUSG0	mmu:7680	5297339	1983428	4768014	5121606	3217430	5296026	3961928	2956726	882176.5
High	Q9D0R4	Probable ATP-dependent RNA helicase DDX56 OS=Mus musculus OX=10090 GN=Ddx56 PE=1 SV=1	Ddx56	7	3	16	3	546	61.2	9.17	52513	ENSMUSG0	mmu:5251	2048491	1389883	2463860		2469576				
High	Q99LD4	COP9 signalosome complex subunit 1 OS=Mus musculus OX=10090 GN=Gps1 PE=1 SV=1	Gps1	13	7	23	7	471	53.4	6.84			B0QZT1; Q8		2374058	4023258	3557520	2541553	2026926	5237648	5938706	3389767
High	P62259	14-3-3 protein epsilon OS=Mus musculus OX=10090 GN=Ywhae PE=1 SV=1	Ywhae	10	3	23	1	255	29.2	4.74	22627	ENSMUSG0	mmu:2262	609805.9		819954.4	1445341		2303379	2715901		2611871
High	O70133	ATP-dependent RNA helicase A OS=Mus musculus OX=10090 GN=Dhx9 PE=1 SV=2	Dhx9	10	9	27	9	1380	149.4	6.83	13211		mmu:1321	4946889	974149.7	3750011	4760845	807204.6	6173994	3043498	1014628	546864.6
High	Q8VI75	Importin-4 OS=Mus musculus OX=10090 GN=Ipo4 PE=1 SV=1	Ipo4	5	4	19	4	1082	119.2	5.03	75751	ENSMUSG0	mmu:7575	1788520	1364983	6375306		4267786	4862537		3498992	902108.8
High	Q8CHY6	Transcriptional repressor p66 alpha OS=Mus musculus OX=10090 GN=Gatad2a PE=1 SV=2	Gatad2a	21	10	33	10	629	67.3	9.89	234366	ENSMUSG0	mmu:2343	5903176	6914628	15407225	25287856	2018174	20766245	1146144	5706387	2328547
High	Q6P9Z1	SWI/SNF-related matrix-associated actin-dependent regulator of chromatin subfamily D member 3 OS=Mus musculus OX=10090 GN=Smar	Smarcd3	8	3	17	3	483	55	9.35	66993	ENSMUSG0	mmu:6699		5155385	6042070		8468743		1759944		501351.7
High	Q8R081	Heterogeneous nuclear ribonucleoprotein L OS=Mus musculus OX=10090 GN=Hnrnpl PE=1 SV=2	Hnrnpl	9	5	22	5	586	63.9	8.1	15388	ENSMUSG0	mmu:1538	579722.2	4273522	5426414	6953727	6181604	7872338	7001679	1881744	348051
High	Q9WU42	Nuclear receptor corepressor 2 OS=Mus musculus OX=10090 GN=Ncor2 PE=1 SV=3	Ncor2	9	15	29	15	2472	269.6	7.44	20602	ENSMUSG0	E9Q9V0; m		5552138	4725297		543307.6				
High	Q8K2Z4	Condensin complex subunit 1 OS=Mus musculus OX=10090 GN=Ncapd2 PE=1 SV=2	Ncapd2	5	6	16	6	1392	155.6	6.38	68298		mmu:6829	3129728	1686983	11501180	810545.4	1592076	3556619	2185950	1969159	409129.1
High	P70333	Heterogeneous nuclear ribonucleoprotein H2 OS=Mus musculus OX=10090 GN=Hnrnph2 PE=1 SV=1	Hnrnph2	12	4	24	1	449	49.2	6.3	56258	ENSMUSG0	mmu:5625									
High	Q8BMA6	Signal recognition particle subunit SRP68 OS=Mus musculus OX=10090 GN=Srp68 PE=1 SV=2	Srp68	15	6	22	6	625	70.5	8.57	217337	ENSMUSG0	A2AAN1; m	68524896	26553222	84617763	53512108	16893544	1.71E+08	19572181	1.3E+08	44187256
High	Q9DC51	Guanine nucleotide-binding protein G(i) subunit alpha-3 OS=Mus musculus OX=10090 GN=Gnai3 PE=1 SV=3	Gnai3	7	2	36	1	354	40.5	5.69	14679	ENSMUSG0	A2AE36; m									
High	Q8CBY1	Protein Smaug homolog 1 OS=Mus musculus OX=10090 GN=Samd4a PE=1 SV=2	Samd4a	3	2	20	2	711	78.3	7.97	74480	ENSMUSG0	A6H6C5; m		2355955	4916904	639234.7	1447131	610689	2651403		554336.6
High	E9PVA8	eIF-2-alpha kinase activator GCN1 OS=Mus musculus OX=10090 GN=Gcn1 PE=1 SV=1	Gcn1	5	11	32	11	2671	292.8	7.36	231659	ENSMUSG0	B2RWW6; E	1423388	2430815	5633794	3242224	1238110	7197102	2518309	8378460	
High	F8VPU2	FERM, ARHGEF and pleckstrin domain-containing protein 1 OS=Mus musculus OX=10090 GN=Farp1 PE=1 SV=1	Farp1	3	2	16	2	1048	118.8	7.88	223254	ENSMUSG0	C4IXU2; F8		1360804	3098223	6750539	2315544	4986231	3456159	2355273	1363659
High	Q9WU40	Inner nuclear membrane protein Man1 OS=Mus musculus OX=10090 GN=Lemd3 PE=1 SV=2	Lemd3	11	6	24	6	921	100.2	7.55	380664		mmu:3806	448711.5	5185806	11770607	1619352	4806254		3198393		
High	O08784	Treacle protein OS=Mus musculus OX=10090 GN=Tcof1 PE=1 SV=1	Tcof1	9	8	20	8	1320	134.9	9.35	21453	ENSMUSG0	mmu:2145	1077588	9098737	8474795		4153262	2832492	2954519	12540728	2597200
High	P08553	Neurofilament medium polypeptide OS=Mus musculus OX=10090 GN=Nefm PE=1 SV=4	Nefm	6	5	36	3	848	95.9	4.77	18040		A2VCT5; m	4296351	3451687	6001752	1401350	1753149	1101517			
High	Q9D2R0	Acetoacetyl-CoA synthetase OS=Mus musculus OX=10090 GN=Aacs PE=1 SV=1	Aacs	7	5	16	5	672	75.2	6.71	78894	ENSMUSG0	A1IG47; m	1383212	1784041	2457597	6118410	437897.7	6710191	4260690	28832279	7248148
High	Q9Z277	Tyrosine-protein kinase BAZ1B OS=Mus musculus OX=10090 GN=Baz1b PE=1 SV=2	Baz1b	2	4	15	4	1479	170.5	8.6	22385	ENSMUSG0	B9EJ99; m	1444395	2040573	6774417	1094398	919063.7	994954.3	1134371		
High	Q6PDH0	Pleckstrin homology-like domain family B member 1 OS=Mus musculus OX=10090 GN=Phldb1 PE=1 SV=1	Phldb1	11	12	22	12	1371	150	8.87	102693	ENSMUSG0	mmu:1026	2059652	3881790	7429326	3296309	1660834	3910999	3161608	11210359	4987650
High	P68254	14-3-3 protein theta OS=Mus musculus OX=10090 GN=Ywhaq PE=1 SV=1	Ywhaq	15	4	25	1	245	27.8	4.78	22630	ENSMUSG0	mmu:2263									
High	P97311	DNA replication licensing factor MCM6 OS=Mus musculus OX=10090 GN=Mcm6 PE=1 SV=1	Mcm6	13	10	24	10	821	92.8	5.45	17219	ENSMUSG0	mmu:1721	5103119	2596636	12711049		241416	4713134	4768827	8047488	279624.2
High	Q91W90	Thioredoxin domain-containing protein 5 OS=Mus musculus OX=10090 GN=Txndc5 PE=1 SV=2	Txndc5	22	9	31	9	417	46.4	5.78	105245	ENSMUSG0	mmu:1052	12057405	2556223	5879607	2826535	401668.4	6610549	2929130	7965908	2647846
High	Q6A0A9	Constitutive coactivator of PPAR-gamma-like protein 1 OS=Mus musculus OX=10090 GN=FAM120A PE=1 SV=2	FAM120A	6	5	20	5	1112	121.6	8.92	218236	ENSMUSG0	A0PJK6; B3	2406481	2527753	7098468	5303352	2908809	5662113	5423739	4319077	4459691
High	P28740-2	Isoform 2 of Kinesin-like protein KIF2A OS=Mus musculus OX=10090 GN=Kif2a	Kif2a	6	3	14	3	659	74.8	6.46			P28740-2	2432871	1243863	5956421	1190774	2406026	3077495		1465908	
High	Q810A7	ATP-dependent RNA helicase DDX42 OS=Mus musculus OX=10090 GN=Ddx42 PE=1 SV=3	Ddx42	11	12	27	12	929	101.9	6.98	72047	ENSMUSG0	mmu:7204	1270984	1222140	9954424	1149351	2665413		2739854	6210989	366724.7
High	P24860	G2/mitotic-specific cyclin-B1 OS=Mus musculus OX=10090 GN=Ccnb1 PE=1 SV=3	Ccnb1	4	2	9	2	430	48	7.43	268697	ENSMUSG0	mmu:2686	901918.8	2721511	6253536		844456.9	794605.9		1726267	611707.2
High	Q00612	Glucose-6-phosphate 1-dehydrogenase X OS=Mus musculus OX=10090 GN=G6pdx PE=1 SV=3	G6pdx	10	5	18	5	515	59.2	6.49	14381	ENSMUSG0	mmu:1438	6242245	1901370	4164711	2022939	2518673	6369507	1147914	4201858	3318901
High	P48722	Heat shock 70 kDa protein 4L OS=Mus musculus OX=10090 GN=Hspa4l PE=1 SV=2	Hspa4l	10	8	35	7	838	94.3	5.74	18415	ENSMUSG0	mmu:1841	1886567	1324708	8108556	5119369	3175974	6595890	2788876	7227890	
High	Q61738	Integrin alpha-7 OS=Mus musculus OX=10090 GN=Itga7 PE=1 SV=3	Itga7	5	5	16	5	1179	129.2	5.71			O88731; O8	6383879	173344.5	1922041	13690697	906726.5	3331804	3792434	23664030	234049.7
High	Q91YR7	Pre-mRNA-processing factor 6 OS=Mus musculus OX=10090 GN=Prpf6 PE=1 SV=1	Prpf6	7	6	21	6	941	106.7	8.09	68879	ENSMUSG0	mmu:6887	3509605	568292.5	9827303	3908718	796297.2	4815715	845064.3	4041657	1318448
High	Q99K51	Plastin-3 OS=Mus musculus OX=10090 GN=Pls3 PE=1 SV=3	Pls3	16	8	34	8	630	70.7	5.62	102866	ENSMUSG0	B1AX56; m	400387.1	2672235	5426834	3899260	2885985	6504592	5949667	6653744	4559933
High	Q6P9R2	Serine/threonine-protein kinase OSR1 OS=Mus musculus OX=10090 GN=Oxsr1 PE=1 SV=1	Oxsr1	6	4	16	4	527	58.2	6.43	108737	ENSMUSG0	mmu:1087	1106908	3203284	5614196	2515991	5083226	2610365	2298095	5471569	2864370
High	Q8CI08	SLAIN motif-containing protein 2 OS=Mus musculus OX=10090 GN=Slain2 PE=1 SV=2	Slain2	12	5	17	5	581	62.3	9.47	75991	ENSMUSG0	mmu:7599	2314927	3752357	5703805	4373012	2970930	2330686	2623493	6426475	
High	Q8BKX1	Brain-specific angiogenesis inhibitor 1-associated protein 2 OS=Mus musculus OX=10090 GN=Baiap2 PE=1 SV=2	Baiap2	16	5	18	5	535	59.2	9.04	108100	ENSMUSG0	mmu:1081	1119835	2643282	4772702		1853988	1178137			445367.3
High	Q9R0Y5	Adenylate kinase isoenzyme 1 OS=Mus musculus OX=10090 GN=Ak1 PE=1 SV=1	Ak1	40	6	14	6	194	21.5	5.81	11636	ENSMUSG0	A2AK80; m	316567.1			986978.6		1217571	2083432	15837612	7286536
High	Q6NVF9	Cleavage and polyadenylation specificity factor subunit 6 OS=Mus musculus OX=10090 GN=Cpsf6 PE=1 SV=1	Cpsf6	8	4	18	4	551	59.1	7.15	432508	ENSMUSG0	mmu:4325	1072690	5519695	10392444	7181382	5262018	8393401	7367840	813074.3	2033887
High	Q925I1	ATPase family AAA domain-containing protein 3 OS=Mus musculus OX=10090 GN=Atad3 PE=1 SV=1	Atad3	13	6	17	6	591	66.7	9.29	108888	ENSMUSG0	A2AD89; m	848323.9	2789746	2832410		1329186	1052150			

High	Q52KR3	Protein prune homolog 2 OS=Mus musculus OX=10090 GN=Prune2 PE=1 SV=2	Prune2	2	5	15	5	3084	339.3	4.46	353211	ENSMUSG0	mmu:3532	831974.7	3493758	2967061	5101086	3204485	2448318	6056141	1739039	1611694
High	P70297	Signal transducing adapter molecule 1 OS=Mus musculus OX=10090 GN=Stam PE=1 SV=3	Stam	7	4	27	4	548	59.7	4.84	20844	ENSMUSG0	mmu:2084		2333831	5202314	1807743	3185069	3489330	5512147	5748502	1303534
High	P12382	ATP-dependent 6-phosphofructokinase, liver type OS=Mus musculus OX=10090 GN=Pfkl PE=1 SV=4	Pfkl	4	3	18	1	780	85.3	7.17	18641	ENSMUSG0	mmu:1864	1362659	1301796	2579625	5800212	1113593	2512683	4363006	3373899	
High	Q62266	Cornifin-A OS=Mus musculus OX=10090 GN=Sprr1a PE=1 SV=1	Sprr1a	71	9	39	9	144	15.8	7.85	20753	ENSMUSG0	mmu:2075	18941117	31937907	17604309	992689.3	2287273	1977557		847882.8	352016.4
High	P14685	26S proteasome non-ATPase regulatory subunit 3 OS=Mus musculus OX=10090 GN=Psmd3 PE=1 SV=3	Psmd3	26	13	18	13	530	60.7	8.44	22123	ENSMUSG0	mmu:2212	10063351	235265.9				1215983	2089595	8507169	1934415
High	P28656	Nucleosome assembly protein 1-like 1 OS=Mus musculus OX=10090 GN=Nap1l1 PE=1 SV=2	Nap1l1	26	9	24	7	391	45.3	4.46	53605	ENSMUSG0	mmu:5360	10144267	2410889	6428304	5110430	6509782	4974883	699192.2	7457822	2690214
High	P98083	SHC-transforming protein 1 OS=Mus musculus OX=10090 GN=Shc1 PE=1 SV=3	Shc1	6	2	11	2	579	62.6	6.54	20416	ENSMUSG0	mmu:2041	745095.1	2420936	3971278	3195424	1987614	2692585	2254242		
High	Q78ZA7	Nucleosome assembly protein 1-like 4 OS=Mus musculus OX=10090 GN=Nap1l4 PE=1 SV=1	Nap1l4	22	6	24	4	375	42.7	4.67	17955	ENSMUSG0	mmu:1795	2090077	3010356	3275517	3954082	6765590	4167434	4188218	4104337	1560940
High	P30285	Cyclin-dependent kinase 4 OS=Mus musculus OX=10090 GN=Cdk4 PE=1 SV=1	Cdk4	8	2	18	1	303	33.7	6.62	12567	ENSMUSG0	mmu:1256	1404067		1460993			2402357		4036616	
High	Q8C8U0	Liprin-beta-1 OS=Mus musculus OX=10090 GN=Ppfibp1 PE=1 SV=3	Ppfibp1	9	6	27	5	969	108.5	5.49	67533	ENSMUSG0	mmu:6753	1275726	519309.6	3807837	2231093	983869.9	1074955	1149361	4821151	1739904
High	P05977	Myosin light chain 1/3, skeletal muscle isoform OS=Mus musculus OX=10090 GN=Myl1 PE=1 SV=2	Myl1	33	5	14	3	188	20.6	5.03	17901	ENSMUSG0	mmu:1790				2569663			4276037	11835103	6072824
High	Q8R5H1	Ubiquitin carboxyl-terminal hydrolase 15 OS=Mus musculus OX=10090 GN=Usp15 PE=1 SV=1	Usp15	6	4	16	4	981	112.3	5.17	14479	ENSMUSG0	mmu:1447	488738.7		6062087		2128857	1274462		9395141	1279721
High	Q6P4S6	Serine/threonine-protein kinase SIK3 OS=Mus musculus OX=10090 GN=Sik3 PE=1 SV=4	Sik3	3	2	12	2	1369	150.6	7.02	70661	ENSMUSG0	E9PU87; m	32052483	12096039	31976991	13751347	5040772	25402109	15738086	29291943	11385971
High	Q9WUK2	Eukaryotic translation initiation factor 4H OS=Mus musculus OX=10090 GN=Eif4h PE=1 SV=3	Eif4h	15	3	15	3	248	27.3	7.23	22384	ENSMUSG0	mmu:2238		3825791	4302976	13647021	3406474	4360396	13595425		
High	Q04750	DNA topoisomerase 1 OS=Mus musculus OX=10090 GN=Top1 PE=1 SV=2	Top1	9	7	18	7	767	90.8	9.33	21969	ENSMUSG0	A2A4B7; m	1937241	1049843	3320521		621384	1566891		6731936	1927946
High	P32233	Developmentally-regulated GTP-binding protein 1 OS=Mus musculus OX=10090 GN=Drg1 PE=1 SV=1	Drg1	23	8	28	7	367	40.5	8.9	13494	ENSMUSG0	mmu:1349	4119326	642385.3	2913121	3477951	399510.5	6563946	868831.4	11060630	4104172
High	Q9JIH2	Nuclear pore complex protein Nup50 OS=Mus musculus OX=10090 GN=Nup50 PE=1 SV=3	Nup50	6	2	12	2	466	49.5	6.24	18141	ENSMUSG0	mmu:1814	433927.1	1919040	3041810	802346.9	2172347	1635503	969791.1	1129234	352099.6
High	Q8K1M6	Dynamin-1-like protein OS=Mus musculus OX=10090 GN=Dnm1l PE=1 SV=2	Dnm1l	12	9	39	9	742	82.6	7.05	74006	ENSMUSG0	mmu:7400	1088483	2192259	6181802	2923399	2266391	5133491	2077023	11938352	2880349
High	Q99KV1	DnaJ homolog subfamily B member 11 OS=Mus musculus OX=10090 GN=Dnajb11 PE=1 SV=1	Dnajb11	12	5	20	5	358	40.5	6.32	67838	ENSMUSG0	A6X957; m	8773399	5036086	7269314	5691771	2800964	3347847	1116497	4358950	3786742
High	Q91ZR2	Sorting nexin-18 OS=Mus musculus OX=10090 GN=Snx18 PE=1 SV=1	Snx18	7	3	10	3	614	67.9	6.67			Q91ZR2		2347772	4467051	3158431	1724356	1428674	724531.1	3582886	
High	Q6DFW4	Nucleolar protein 58 OS=Mus musculus OX=10090 GN=Nop58 PE=1 SV=1	Nop58	17	8	23	8	536	60.3	8.34	55989	ENSMUSG0	mmu:5598	4981320	504772.8	2447238	2006899	1323925	7833749	834819.9	7451012	2858380
High	Q9ER72	Cysteine--tRNA ligase, cytoplasmic OS=Mus musculus OX=10090 GN=Cars1 PE=1 SV=2	Cars1	4	4	15	4	831	94.8	6.76	27267	ENSMUSG0	mmu:2726	1296329	1147305	6033562	3703975	1063772	4905089	3475872		443244.8
High	Q8C650	Septin-10 OS=Mus musculus OX=10090 GN=Septin10 PE=1 SV=1	Septin10	5	2	16	1	452	52.4	6.6	103080		mmu:1030									
High	Q69ZN7	Myoferlin OS=Mus musculus OX=10090 GN=Myof PE=1 SV=2	Myof	4	8	21	8	2048	233.2	6.16	226101	ENSMUSG0	mmu:2261	4254491	8296232	2644335	10704099	3007845	7747943	6497029	2968075	
High	Q9CXW3	Calcyclin-binding protein OS=Mus musculus OX=10090 GN=Cacybp PE=1 SV=1	Cacybp	28	8	23	8	229	26.5	7.87	12301	ENSMUSG0	mmu:1230	2635610	5863275	9986787	2346214	2553744	9658032	7617231	22251852	3278548
High	P83741	Serine/threonine-protein kinase WNK1 OS=Mus musculus OX=10090 GN=Wnk1 PE=1 SV=2	Wnk1	4	10	35	10	2377	250.8	6.43	232341	ENSMUSG0	A6H6V1; B2	1756898	10245915	10275982	9979782	5333034	5223865	11517468	3785990	3310752
High	Q9ER73	Elongator complex protein 4 OS=Mus musculus OX=10090 GN=Elp4 PE=1 SV=2	Elp4	10	3	16	3	422	46.3	8.78	77766	ENSMUSG0	mmu:7776		3065363	1719990		1076928	1589507			
High	Q9JK48	Endophilin-B1 OS=Mus musculus OX=10090 GN=Sh3glb1 PE=1 SV=1	Sh3glb1	18	7	19	7	365	40.8	6.04	54673	ENSMUSG0	mmu:5467	430128.4	5018312	7582294	21676629	5564972	8174330	14061100	14130335	3233134
High	Q80U87	Ubiquitin carboxyl-terminal hydrolase 8 OS=Mus musculus OX=10090 GN=Usp8 PE=1 SV=2	Usp8	6	5	18	5	1080	122.5	8.47	84092	ENSMUSG0	A2AI53; mn	1808834	2989946	8468531	3899040	2641879	1795124	3260399		
High	O88844	Isocitrate dehydrogenase [NADP] cytoplasmic OS=Mus musculus OX=10090 GN=Idh1 PE=1 SV=2	Idh1	13	4	26	3	414	46.6	7.17	15926	ENSMUSG0	mmu:1592				1553714			3192444		556143.1
High	O54988	STE20-like serine/threonine-protein kinase OS=Mus musculus OX=10090 GN=Slk PE=1 SV=2	Slk	14	14	33	14	1233	141.4	5.14	20874	ENSMUSG0	A2RRK4; m	625938.5	10862473	15457606	9109855	6637037	8681541	7340603	13819018	8853199
High	O88532	Zinc finger RNA-binding protein OS=Mus musculus OX=10090 GN=Zfr PE=1 SV=2	Zfr	6	4	11	4	1074	116.8	9.04	22763	ENSMUSG0	mmu:2276			2088850		714376.6				
High	Q8VBT9	Tether containing UBX domain for GLUT4 OS=Mus musculus OX=10090 GN=Aspscr1 PE=1 SV=1	Aspscr1	13	5	23	5	550	59.8	6.96	68938	ENSMUSG0	A2ABZ7; A2		4006464	3177893	5785642	4443575	2299710	5388675		
High	P62874	Guanine nucleotide-binding protein G(I)/G(S)/G(T) subunit beta-1 OS=Mus musculus OX=10090 GN=Gnb1 PE=1 SV=3	Gnb1	11	3	7	2	340	37.4	6	14688	ENSMUSG0	mmu:1468	157102.9			798166.5	368155.5		880018.4	3196718	2141789
High	P26040	Ezrin OS=Mus musculus OX=10090 GN=Ezr PE=1 SV=3	Ezr	5	3	18	1	586	69.4	6.1	22350	ENSMUSG0	mmu:2235		997392.9	1968624	1764038	1144803	1329002	2873428		
High	Q8K4B0	Metastasis-associated protein MTA1 OS=Mus musculus OX=10090 GN=Mta1 PE=1 SV=1	Mta1	8	6	15	5	715	80.7	9.29		ENSMUSG0	Q80UI1; Q8	5154833	1915110	5765995	730998.7		1778930		4005733	454138.4
High	Q9WVK4	EH domain-containing protein 1 OS=Mus musculus OX=10090 GN=Ehd1 PE=1 SV=1	Ehd1	14	10	21	7	534	60.6	6.83	13660	ENSMUSG0	mmu:1366	1290695			1455917		1258773	1015675	6312234	1557128
High	Q9WTQ5	A-kinase anchor protein 12 OS=Mus musculus OX=10090 GN=Akap12 PE=1 SV=1	Akap12	11	15	26	15	1684	180.6	4.44	83397	ENSMUSG0	mmu:8339		8572027	4366843	7881815	1057880	887245.8	13820813	5463287	
High	Q91X78	Erlin-1 OS=Mus musculus OX=10090 GN=Erlin1 PE=1 SV=2	Erlin1	22	8	21	4	348	39.2	7.21	226144	ENSMUSG0	A0A0R4J1G	5443019	304929.4	400938.4	958480.9	581626.1	1718708	3094250		233255.9
High	Q921C5	Protein bicaudal D homolog 2 OS=Mus musculus OX=10090 GN=Bicd2 PE=1 SV=1	Bicd2	6	5	24	5	820	93.3	5.44	76895	ENSMUSG0	mmu:7689	1572891	2606329	12562118	15601216	3952090	7344183	8674220	14584470	4068212
High	Q62419	Endophilin-A2 OS=Mus musculus OX=10090 GN=Sh3gl1 PE=1 SV=1	Sh3gl1	8	3	17	3	368	41.5	5.72	20405	ENSMUSG0	mmu:2040	21598803	18811121	23745969	15480112	17883914	21351877	14643711	19841384	6977954
High	Q9WV80	Sorting nexin-1 OS=Mus musculus OX=10090 GN=Snx1 PE=1 SV=1	Snx1	9	4	14	2	522	58.9	5.22	56440		mmu:5644	950243.2	710038.2	3506913	1410861	592741.9	3629880	2274479	6097661	2481596
High	P42669	Transcriptional activator protein Pur-alpha OS=Mus musculus OX=10090 GN=Pura PE=1 SV=1	Pura	15	8	26	4	321	34.9	6.44	19290	ENSMUSG0	mmu:1929	632610			178151	1014449	1273725	432892.5	1915969	2099885
High	Q4VAA2	Protein CDV3 OS=Mus musculus OX=10090 GN=Cdv3 PE=1 SV=2	Cdv3	19	2	16	2	281	29.7	6.1	321022	ENSMUSG0	mmu:3210		2000005	3618775	7162869	2264613		8572761		
High	Q9R0N0	Galactokinase OS=Mus musculus OX=10090 GN=Galk1 PE=1 SV=2	Galk1	16	6	15	6	392	42.3	5.26	14635	ENSMUSG0	mmu:1463	2117556	2964762	2684508	2082649	4457099	2368652	1328895	9245668	4474830
High	E9Q6J5	Biorientation of chromosomes in cell division protein 1-like 1 OS=Mus musculus OX=10090 GN=Bod1l PE=1 SV=1	Bod1l	3	8	18	8	3032	327.3	5.33	665775	ENSMUSG0	E9Q6J5; m	1152306	6448271	6874281	953415	395844.2		813799		
High	Q62351	Transferrin receptor protein 1 OS=Mus musculus OX=10090 GN=Tfrc PE=1 SV=1	Tfrc	9	7	35	7	763	85.7	6.57	22042	ENSMUSG0	mmu:2204	4705057	677042.1	7658951	10508683	2789798	8621412	4278364	3861866	3578163
High	Q9D824	Pre-mRNA 3'-end-processing factor FIP1 OS=Mus musculus OX=10090 GN=Fip1l1 PE=1 SV=1	Fip1l1	5	2	10	2	581	64.9	5.77	66899	ENSMUSG0	mmu:6689		1053435	3952316	2955379	491031.8	4164394	1814647		475009
High	Q6PDQ2	Chromodomain-helicase-DNA-binding protein 4 OS=Mus musculus OX=10090 GN=Chd4 PE=1 SV=1	Chd4	2	2	13	2	1915	217.6	5.81	107932	ENSMUSG0	mmu:1079	1594201	3077915	4920248	5025152	2265693	4312233	2226825		
High	Q80WJ7	Protein LYRIC OS=Mus musculus OX=10090 GN=Mtdh PE=1 SV=1	Mtdh	7	3	22	3	579	63.8	9.33	67154	ENSMUSG0	B2RSG8; m	289249.6	8680894	7550886	7211290	4736510	1507113	10197729		
High	Q8BH59	Electrogenic aspartate/glutamate antiporter SLC25A12, mitochondrial OS=Mus musculus OX=10090 GN=Slc25a12 PE=1 SV=1	Slc25a12	19	11	31	11	677	74.5	8.25	78830	ENSMUSG0	A2BFG0; m	2685519	740149.6	2483898	1504318		3918157	7539488	15203806	4081047
High	Q05186	Reticulocalbin-1 OS=Mus musculus OX=10090 GN=Rcn1 PE=1 SV=1	Rcn1	13	5	19	5	325	38.1	4.84	19672	ENSMUSG0	mmu:1967	6933596	1453170	4695338	1818908	623378.8	5567482	2228770	2085255	1712755
High	P30416	Peptidyl-prolyl cis-trans isomerase FKBP4 OS=Mus musculus OX=10090 GN=Fkbp4 PE=1 SV=5	Fkbp4	10	4	20	4	458	51.5	5.72	14228	ENSMUSG0	mmu:1422		3851795	5022709		4362297	4670296		4176735	244372.1
High	Q8K3X4	Probable E3 ubiquitin-protein ligase IRF2BPL OS=Mus musculus OX=10090 GN=Irf2bpl PE=1 SV=1	Irf2bpl	7	3	10	3	775	80.5	8.24	238330	ENSMUSG0	mmu:2383		1746105	4636815	2402321	665960.6	751524.3	1129178	1347825	
High	Q8K4J6	Myocardin-related transcription factor A OS=Mus musculus OX=10090 GN=Mrtfa PE=1 SV=2	Mrtfa	7	5	16	4	964	102.5	5.5	223701	ENSMUSG0	mmu:2237	751736.3	2662259	8073595	1238504	2365883		2504696	2119783	850221.5
High	Q8VCM8	BOS complex subunit NCLN OS=Mus musculus OX=10090 GN=Ncln PE=1 SV=2	Ncln	3	2	17	2	563	62.9	6.49	103425	ENSMUSG0	mmu:1034	1956586	1349539	2392131	3392657	2259398	2277818	3182782	5384203	868403.9
High	Q9CZX8	40S ribosomal protein S19 OS=Mus musculus OX=10090 GN=Rps19 PE=1 SV=3	Rps19	25	5	16	5	145	16.1	10.4	20085	ENSMUSG0	mmu:2008	717506.4	2715427	2611995			1908272		6829091	7754832
High	Q8QZY1	Eukaryotic translation initiation factor 3 subunit L OS=Mus musculus OX=10090 GN=Eif3l PE=1 SV=1	Eif3l	11	5	9	5	564	66.6	6.44	223691	ENSMUSG0	mmu:2236	12087898	790084.1	2157581	3239211	1093649	2501186	3217024	2823036	1452425
High	Q5SXY1-3	Isoform 4 of Cytospin-B OS=Mus musculus OX=10090 GN=Specc1	Specc1	9	9	25	9	987	109.8	5.86			Q5SXY1-3	5759431	16654106	36272333	13937871	11906628	22340131	11900179	25383138	3109113
High	P97496	SWI/SNF complex subunit SMARCC1 OS=Mus musculus OX=10090 GN=Smarcc1 PE=1 SV=2	Smarcc1	6	5	11	4	1104	122.8	5.73	20588	ENSMUSG0	mmu:2058	1345719	1351232	7356690		1264144		903261.7	4112525	341297.2
High	Q8CGK3	Lon protease homolog, mitochondrial OS=Mus musculus OX=10090 GN=Lonp1 PE=1 SV=2	Lonp1	7	8	17	8	949	105.8	6.57	74142	ENSMUSG0	mmu:7414	235530.3	993704.3	4425919	3157472	1849515	10250168	4903195	24420756	16558171
High	Q9CXY6	Interleukin enhancer-binding factor 2 OS=Mus musculus OX=10090 GN=Ilf2 PE=1 SV=1	Ilf2	12	5	13	5	390	43	5.26	67781	ENSMUSG0	mmu:6778		289518.3		3201951	692072.4	3012804	1247543	4803753	1561633
High	Q61508	Extracellular matrix protein 1 OS=Mus musculus OX=10090 GN=Ecm1 PE=1 SV=2	Ecm1	10	5	17	5	559	62.8	6.8	13601	ENSMUSG0	mmu:1360	37497111	4939820	18631507	10425961	1480038	22415787	9095298	29402589	10331658
High	Q04899	Cyclin-dependent kinase 18 OS=Mus musculus OX=10090 GN=Cdk18 PE=1 SV=1	Cdk18	5	2	18	1	451	51.8	8.51	18557	ENSMUSG0	mmu:1855									
High	Q8R4U7	Leucine zipper protein 1 OS=Mus musculus OX=10090 GN=Luzp1 PE=1 SV=2	Luzp1	4	4	17	4	1068	119.2	7.99	269593	ENSMUSG0	A3KG96; m	1436795	3214754	5991424	3641661	2478250		799191.8		964513
High	Q64337	Sequestosome-1 OS=Mus musculus OX=10090 GN=Sqstm1 PE=1 SV=1	Sqstm1	19	6	17	6	442	48.1	5.21	18412	ENSMUSG0	mmu:1841	6942777	3509749	11655410	2368987	1340274	4117176	2990574	12307825	7479927
High	Q8VDP4	Cell cycle and apoptosis regulator protein 2 OS=Mus musculus OX=10090 GN=Ccar2 PE=1 SV=2	Ccar2	8	6	21	6	922	102.9	5.25	219158	ENSMUSG0	mmu:2191	2809918	1807008	11109665	669172.8	3058577	8546366	1205804	3154044	4002790
High	P45591	Cofilin-2 OS=Mus musculus OX=10090 GN=Cfl2 PE=1 SV=1	Cfl2	24	4	15	3	166	18.7	7.88	12632	ENSMUSG0	mmu:1263	431572.5	211388.3	500354.6	1483377	1622932			10063327	3869948
High	Q69ZA1	Cyclin-dependent kinase 13 OS=Mus musculus OX=10090 GN=Cdk13 PE=1 SV=3	Cdk13	1	2	15	1	1511	164.5	9.69	69562	ENSMUSG0	E9QKZ6; m		916655.3	1299631						
High	P35278	Ras-related protein Rab-5C OS=Mus musculus OX=10090 GN=Rab5c PE=1 SV=2	Rab5c	25	4	14	3	216	23.4	8.41	19345	ENSMUSG0	mmu:1934	1857156		913832.4			1440867		6271633	1282778
High	Q91ZJ5	UTP--glucose-1-phosphate uridylyltransferase OS=Mus musculus OX=10090 GN=Ugp2 PE=1 SV=3	Ugp2	10	6	22	6	508	56.9	7.61	216558	ENSMUSG0	mmu:2165	324236.8	4387277	5435275	11238145	3198455	6101349	11127880	10613290	2762831
High	P40124	Adenylyl cyclase-associated protein 1 OS=Mus musculus OX=10090 GN=Cap1 PE=1 SV=4	Cap1	19	6	19	5	474	51.5	7.52	12331	ENSMUSG0	mmu:1233	3854927	450143.8	2047223	13023020	505273.4	1568152	10991983	4738645	384289.1
High	Q9D0F9	Phosphoglucomutase-1 OS=Mus musculus OX=10090 GN=Pgm1 PE=1 SV=4	Pgm1	12	6	9	6	562	61.4	6.57	72157	ENSMUSG0	mmu:7215	510446.1	1630728	4532143	1916775	394582.2	2493912	1780856	1117734	5041706
High	Q91YH5	Atlastin-3 OS=Mus musculus OX=10090 GN=Atl3 PE=1 SV=1	Atl3	3	2	12	2	541	60.5	6.1	109168	ENSMUSG0	mmu:1091				2519877	348265.2	2474473	1894220	2452391	435611.5
High	O08808	Protein diaphanous homolog 1 OS=Mus musculus OX=10090 GN=Diaph1 PE=1 SV=1	Diaph1	4	5	15	5	1255	139.3	5.57	13367	ENSMUSG0	mmu:1336	7190849	3052373	15248416	6731620	4082430	8757893	5148619	20868146	6480837
High	P55302	Alpha-2-macroglobulin receptor-associated protein OS=Mus musculus OX=10090 GN=Lrpap1 PE=1 SV=1	Lrpap1	16	6	21	6	360	42.2	7.87	16976	ENSMUSG0	mmu:1697	2788349	1339058	2592919	2185942	752042.1	1847404	1490018		
High	P83882	60S ribosomal protein L36a OS=Mus musculus OX=10090 GN=Rpl36a PE=1 SV=2	Rpl36a	24	3	12	3	106	12.4	10.58	19982; 664	ENSMUSG0	mmu:1998	4908261	2760492	2069866	670110.4	680565.6	1306259	2217907		1956648
High	P02463	Collagen alpha-1(IV) chain OS=Mus musculus OX=10090 GN=Col4a1 PE=1 SV=4	Col4a1	4	5	17	5	1669	160.6	8.24	12826	ENSMUSG0	mmu:1282	14325094	691373.2	5982578	2776259	986083.8	3356325	2398145	2141927	919690.3
High	Q5F2E7	FMR1-interacting protein NUFIP2 OS=Mus musculus OX=10090 GN=Nufip2 PE=1 SV=1	Nufip2	15	8	23	8	692	75.6	8.7	68564	ENSMUSG0	mmu:6856	14553022	6386993	18488293	4596401	5286695	6829175	1701010	12546395	6386670
High	Q99KI0	Aconitate hydratase, mitochondrial OS=Mus musculus OX=10090 GN=Aco2 PE=1 SV=1	Aco2	9	6	15	6	780	85.4	7.93	11429	ENSMUSG0	mmu:1142	276342.4		855276.7			2080633	5329756		5215640
High	P05201	Aspartate aminotransferase, cytoplasmic OS=Mus musculus OX=10090 GN=Got1 PE=1 SV=3	Got1	12	4	7	4	413	46.2	7.14	14718	ENSMUSG0	mmu:1471	1088965	2737336	4602134		3794002	9646693	4779475	26548878	17148021
High	Q8BQM4	HEAT repeat-containing protein 3 OS=Mus musculus OX=10090 GN=Heatr3 PE=1 SV=1	Heatr3	6	4	14	4	679	74.3	5.01	234549	ENSMUSG0	mmu:2345	1414300	5863809	10749331	2321240	1921449	6451926	2177142	3385576	1446561
High	Q64514	Tripeptidyl-peptidase 2 OS=Mus musculus OX=10090 GN=Tpp2 PE=1 SV=3	Tpp2	6	8	27	8	1262	139.8	6.58	22019	ENSMUSG0	mmu:2201	1761930	2022915	5390307	4202612	1462072	5412226	4442623	2767101	1790331
High	Q6NZF1	Zinc finger CCCH domain-containing protein 11A OS=Mus musculus OX=10090 GN=Zc3h11a PE=1 SV=1	Zc3h11a	9	6	18	6	792	86.4	8.13	70579	ENSMUSG0	mmu:7057	381220.8	725043.7	7749955		2469458	2631488			
High	Q8C1A5	Thimet oligopeptidase OS=Mus musculus OX=10090 GN=Thop1 PE=1 SV=1	Thop1	19	11	17	11	687	78	6.06	50492		mmu:5049			1873567	3575291	283375.7	1273663	2714027	20163262	29553687
High	P63073	Eukaryotic translation initiation factor 4E OS=Mus musculus OX=10090 GN=Eif4e PE=1 SV=1	Eif4e	31	6	19	6	217	25	6.15	13684	ENSMUSG0	mmu:1368	1504886	1455976	2273756	692422.2		2090172	1615432	15833314	5554599
High	P02468	Laminin subunit gamma-1 OS=Mus musculus OX=10090 GN=Lamc1 PE=1 SV=2	Lamc1	7	10	16	10	1607	177.2	5.21			P02468	4216315	97139.83	4913292	21968066	1281002	17893596	7953805	39179849	18337794
High	P54103	DnaJ homolog subfamily C member 2 OS=Mus musculus OX=10090 GN=Dnajc2 PE=1 SV=2	Dnajc2	7	5	15	5	621	71.7	8.7	22791	ENSMUSG0	mmu:2279	904441.2	3659150	7884633	1450010	2573801	4826905	1737273	6349232	1773072
High	Q8CI94	Glycogen phosphorylase, brain form OS=Mus musculus OX=10090 GN=Pygb PE=1 SV=3	Pygb	6	5	11	3	843	96.7	6.73	110078	ENSMUSG0	mmu:1100	2538434	349007.8	1090232			1740393	912038.4	5454386	1267226
High	Q62189	U1 small nuclear ribonucleoprotein A OS=Mus musculus OX=10090 GN=Snrpa PE=1 SV=3	Snrpa	7	2	11	2	287	31.8	9.8	53607	ENSMUSG0	mmu:5360		1328111	3497179	10061774	1551753	4686342	26704245	4493766	2153679
High	Q0GNC1	Inverted formin-2 OS=Mus musculus OX=10090 GN=Inf2 PE=1 SV=1	Inf2	7	8	19	8	1273	138.5	5.21	70435		mmu:7043	4217168	3545387	14423978	2946426	2197546	4781134	2566736	7585193	2165153
High	Q99JZ7	ERBB receptor feedback inhibitor 1 OS=Mus musculus OX=10090 GN=Errfi1 PE=1 SV=1	Errfi1	11	3	11	3	461	50	7.72	74155	ENSMUSG0	mmu:7415	622004.6	2807197	959393.3						
High	Q61545	RNA-binding protein EWS OS=Mus musculus OX=10090 GN=Ewsr1 PE=1 SV=2	Ewsr1	10	5	9	5	655	68.4	9.33	14030	ENSMUSG0	mmu:1403		2060139	1626951		733787.8				
High	Q99J95	Cyclin-dependent kinase 9 OS=Mus musculus OX=10090 GN=Cdk9 PE=1 SV=1	Cdk9	5	2	15	1	372	42.7	8.79	107951	ENSMUSG0	B0R020; m									
High	Q61851	Fibroblast growth factor receptor 3 OS=Mus musculus OX=10090 GN=Fgfr3 PE=1 SV=1	Fgfr3	2	2	15	1	801	87.7	6.32			Q61564; Q6									
High	P17710	Hexokinase-1 OS=Mus musculus OX=10090 GN=Hk1 PE=1 SV=3	Hk1	12	12	40	9	974	108.2	6.8	15275	ENSMUSG0	E9PXQ3; P1	10842683	48649369	50415000	23074735	14338571	26188264	29007863	32797176	11382159
High	Q3UHU5	Microtubule cross-linking factor 1 OS=Mus musculus OX=10090 GN=Mtcl1 PE=1 SV=1	Mtcl1	1	2	11	2	1945	213.7	6.14	68617	ENSMUSG0	mmu:6861		4173053	4690926	1135718	2123970	2002062			451463.6
High	Q9CW03	Structural maintenance of chromosomes protein 3 OS=Mus musculus OX=10090 GN=Smc3 PE=1 SV=2	Smc3	10	10	17	10	1217	141.5	7.18	13006	ENSMUSG0	mmu:1300	874092.3		8975254	3214474	938011.8	1306228	273371.4	4000323	279343
High	P54822	Adenylosuccinate lyase OS=Mus musculus OX=10090 GN=Adsl PE=1 SV=2	Adsl	9	3	31	3	484	54.8	7.27	11564	ENSMUSG0	mmu:1156	5004559	5307224	10035041	2689286	3031275	11453549	6165370	18000651	3242472

High	Q9JMK2	Casein kinase I isoform epsilon OS=Mus musculus OX=10090 GN=Csnk1e PE=1 SV=2	Csnk1e	7	2	19	2	416	47.3	9.66	27373	ENSMUSG0	mmu:2737	1018995	3164807	4486132	3255509	1207656	3835944	2599197	3102220	902800.4
High	Q6P5E4	UDP-glucose:glycoprotein glucosyltransferase 1 OS=Mus musculus OX=10090 GN=Uggt1 PE=1 SV=4	Uggt1	5	7	15	7	1551	176.3	5.62	320011	ENSMUSG0	E9QQ49; m	4340396	847806	1413665	2347535	317388.1	2168143	4296612	13136240	
High	Q6A026	Sister chromatid cohesion protein PDS5 homolog A OS=Mus musculus OX=10090 GN=Pds5a PE=1 SV=3	Pds5a	7	7	14	7	1332	150.2	7.85			E9Q656; Q6	386076.3	1278633	3159029						
High	Q9D1Q6	Endoplasmic reticulum resident protein 44 OS=Mus musculus OX=10090 GN=Erp44 PE=1 SV=1	Erp44	14	5	14	5	406	46.8	5.27	76299	ENSMUSG0	mmu:7629	9844926	5961456	10222888	7280464	2761038	8797659	2768581	25707383	4689836
High	Q80U72	Protein scribble homolog OS=Mus musculus OX=10090 GN=Scrib PE=1 SV=2	Scrib	6	8	18	8	1612	174	5.12	105782	ENSMUSG0	mmu:1057		2110760	3599883	1549274	1121725	1148930	3808706	9769472	2702902
High	P84104	Serine/arginine-rich splicing factor 3 OS=Mus musculus OX=10090 GN=Srsf3 PE=1 SV=1	Srsf3	18	2	8	2	164	19.3	11.65	20383	ENSMUSG0	mmu:2038		632974.5	1807655	1959812	309471	1851424	1645587	2357313	1222492
High	Q80YX1	Tenascin OS=Mus musculus OX=10090 GN=Tnc PE=1 SV=1	Tnc	4	7	10	7	2110	231.7	4.89	21923	ENSMUSG0	mmu:2192	8280471	3336597	6696197	2371248	1483567	8637749		12493374	2908616
High	Q64737	Trifunctional purine biosynthetic protein adenosine-3 OS=Mus musculus OX=10090 GN=Gart PE=1 SV=3	Gart	9	7	21	7	1010	107.4	6.68	14450	ENSMUSG0	mmu:1445	2187721	1366167	7474254	2184345		4529907			3802868
High	Q6ZPY7	Lysine-specific demethylase 3B OS=Mus musculus OX=10090 GN=Kdm3b PE=1 SV=3	Kdm3b	3	3	12	3	1762	191.3	7.23	277250	ENSMUSG0	B9EKS2; m		1950421	2709708	2968619	1661137	2237872	1067019		
High	Q8BHC4	Dephospho-CoA kinase domain-containing protein OS=Mus musculus OX=10090 GN=Dcakd PE=1 SV=1	Dcakd	15	3	9	3	231	26.5	9.58	68087	ENSMUSG0	mmu:6808				618995.9	560948.1	2354427		6117036	2328336
High	O88207	Collagen alpha-1(V) chain OS=Mus musculus OX=10090 GN=Col5a1 PE=1 SV=2	Col5a1	3	4	9	4	1838	183.6	4.98	12831	ENSMUSG0	A3KGE3; m	6925960	540652.1	2295764				673887.1	9802153	2299089
High	Q91ZU6	Dystonin OS=Mus musculus OX=10090 GN=Dst PE=1 SV=2	Dst	1	8	16	5	7393	833.7	5.31	13518	ENSMUSG0	E9PXE5; E9	1589154	8855539	9450579	6343583	7361262	7240860	8624586	9263895	1358246
High	Q60864	Stress-induced-phosphoprotein 1 OS=Mus musculus OX=10090 GN=Stip1 PE=1 SV=1	Stip1	4	2	12	2	543	62.5	6.8	20867	ENSMUSG0	mmu:2086	366060.5	1308757	2136997	2870636	1721637	2881219	4296914		597979.4
High	Q8C5L3	CCR4-NOT transcription complex subunit 2 OS=Mus musculus OX=10090 GN=Cnot2 PE=1 SV=2	Cnot2	6	2	9	2	540	59.7	7.66	72068	ENSMUSG0	mmu:7206	627017.1	763435.6	2439611		767804.6	1311345			
High	Q6P6L0	Filamin A-interacting protein 1-like OS=Mus musculus OX=10090 GN=Filip1l PE=1 SV=2	Filip1l	5	5	18	5	1131	129.7	6.37		ENSMUSG0	B2ZAA3; B3	264533.1	1745793	6165942	751635.2	3505574	1631123	1029554	3509252	902976.5
High	Q8BYK6	YTH domain-containing family protein 3 OS=Mus musculus OX=10090 GN=Ythdf3 PE=1 SV=2	Ythdf3	8	5	31	1	585	63.9	9.04	229096	ENSMUSG0	mmu:2290	2228711	2742418	6092829	3806542	1601481	5060968	1907505	3576384	1070530
High	Q8CI11	Guanine nucleotide-binding protein-like 3 OS=Mus musculus OX=10090 GN=Gnl3 PE=1 SV=2	Gnl3	8	4	12	4	538	60.7	9.11	30877	ENSMUSG0	mmu:3087	758761.7								
High	P97315	Cysteine and glycine-rich protein 1 OS=Mus musculus OX=10090 GN=Csrp1 PE=1 SV=3	Csrp1	17	2	12	2	193	20.6	8.57	13007	ENSMUSG0	mmu:1300	847576.8	1169637	1064214	7521595	1831663	2566144	4116832	2068570	1058195
High	P08249	Malate dehydrogenase, mitochondrial OS=Mus musculus OX=10090 GN=Mdh2 PE=1 SV=3	Mdh2	31	10	24	10	338	35.6	8.68	17448	ENSMUSG0	mmu:1744			1955764					2657430	18873415
High	Q9WTK5	Nuclear factor NF-kappa-B p100 subunit OS=Mus musculus OX=10090 GN=Nfkb2 PE=1 SV=1	Nfkb2	4	3	11	3	899	96.8	6.37	18034	ENSMUSG0	mmu:1803			1847981	2027227	925267.1	1518270			
High	Q9JK92	Heat shock protein beta-8 OS=Mus musculus OX=10090 GN=Hspb8 PE=1 SV=1	Hspb8	23	4	22	4	196	21.5	5.02	80888	ENSMUSG0	mmu:8088		1126361	2160253	7149290	2294106	2348228	2185768	12198649	4872238
High	Q9DBY8	Nuclear valosin-containing protein-like OS=Mus musculus OX=10090 GN=Nvl PE=1 SV=1	Nvl	3	2	13	2	855	94.4	6.35	67459	ENSMUSG0	mmu:6745			3752051						
High	Q99LI8	Hepatocyte growth factor-regulated tyrosine kinase substrate OS=Mus musculus OX=10090 GN=Hgs PE=1 SV=2	Hgs	4	4	13	4	775	86	6.16	15239	ENSMUSG0	Q61691; Q8	1652470	1533474	8890604	6042276	1402452	8504176	6541041	9418516	3733284
High	A2AAY5	SH3 and PX domain-containing protein 2B OS=Mus musculus OX=10090 GN=Sh3pxd2b PE=1 SV=1	Sh3pxd2b	6	4	15	3	908	101.5	8.66	268396	ENSMUSG0	A2AAY5; B6	378444.7	2409313	1270626	3384053	2712932				
High	Q8VDF2	E3 ubiquitin-protein ligase UHRF1 OS=Mus musculus OX=10090 GN=Uhrf1 PE=1 SV=2	Uhrf1	3	2	11	2	782	88.2	8.31	18140	ENSMUSG0	Q3U9D7; Q	191636.9	1603421	5464432	393841.3	1594918		1734.08	256364.5	829784.9
High	Q6A058	Armadillo repeat-containing X-linked protein 2 OS=Mus musculus OX=10090 GN=Armcx2 PE=2 SV=2	Armcx2	5	3	10	3	784	81	9.33	67416	ENSMUSG0	A2AKS5; m		901569.8	1726706		3664877				
High	Q60972	Histone-binding protein RBBP4 OS=Mus musculus OX=10090 GN=Rbbp4 PE=1 SV=5	Rbbp4	5	2	8	1	425	47.6	4.89	19646	ENSMUSG0	A2A875; Q6	1640741	1739506	2876607			1616883		3137209	1639863
High	Q9D154	Leukocyte elastase inhibitor A OS=Mus musculus OX=10090 GN=Serpinb1a PE=1 SV=1	Serpinb1a	6	2	7	2	379	42.5	6.21	66222	ENSMUSG0	mmu:6622	267288.8	275628.8		2451191		2054706		5258986	1303929
High	Q9CQQ7	ATP synthase F(0) complex subunit B1, mitochondrial OS=Mus musculus OX=10090 GN=Atp5pb PE=1 SV=1	Atp5pb	16	4	18	4	256	28.9	9.06	11950	ENSMUSG0	mmu:1195		467737		581807.8	968146.5	2978717	224125.7	10444751	5751472
High	Q8CG47	Structural maintenance of chromosomes protein 4 OS=Mus musculus OX=10090 GN=Smc4 PE=1 SV=1	Smc4	4	5	8	5	1286	146.8	7.3	70099	ENSMUSG0	mmu:7009	3135182	3829148	4718936	2466350	2081643	6089381		1404151	798436.9
High	Q8CH25	SAFB-like transcription modulator OS=Mus musculus OX=10090 GN=Sltm PE=1 SV=1	Sltm	5	4	7	4	1031	116.9	7.72	66660	ENSMUSG0	mmu:6666		705337.4							
High	Q5XJY5	Coatomer subunit delta OS=Mus musculus OX=10090 GN=Arcn1 PE=1 SV=2	Arcn1	12	5	11	5	511	57.2	6.21	213827	ENSMUSG0	mmu:2138	3320593	1483118	4553039	19676322	3625685	7883617	30766121	18554047	5006205
High	Q62470	Integrin alpha-3 OS=Mus musculus OX=10090 GN=Itga3 PE=1 SV=1	Itga3	2	2	9	2	1053	116.7	6.57	16400	ENSMUSG0	mmu:1640	792614.8	1474997	4435389	9482984	1579656	2557100	7703905	3430568	516413.8
High	Q9QZQ1	Afadin OS=Mus musculus OX=10090 GN=Afdn PE=1 SV=3	Afdn	3	4	15	4	1820	206.4	6.32	17356	ENSMUSG0	mmu:1735		4491683	6867573		3008250	1655461	5925439	1836765	
High	P42208	Septin-2 OS=Mus musculus OX=10090 GN=Septin2 PE=1 SV=2	Septin2	18	7	11	7	361	41.5	6.55	18000	ENSMUSG0	B2RRZ2; m	1251998		3516334	6534442	1124153	5055906		15638169	7371066
High	P97386	DNA ligase 3 OS=Mus musculus OX=10090 GN=Lig3 PE=1 SV=2	Lig3	4	3	9	3	1015	113	8.98			E9QL81; P9		54816.57		893187.7	859288	1086914		636644.2	
High	E9Q8D0	DnaJ homolog subfamily C member 21 OS=Mus musculus OX=10090 GN=Dnajc21 PE=1 SV=1	Dnajc21	4	2	6	2	531	61.7	5.9	78244	ENSMUSG0	E9Q8D0; m		2362735	3000370	1068226	1154403				326340.7
High	B2RRE7	OTU domain-containing protein 4 OS=Mus musculus OX=10090 GN=Otud4 PE=1 SV=1	Otud4	5	5	10	5	1107	123	6.76	73945	ENSMUSG0	B2RRE7; B7	1288392	1507188	5285500	801194.9		1306226	713958.3		
High	Q8VCF0	Mitochondrial antiviral-signaling protein OS=Mus musculus OX=10090 GN=Mavs PE=1 SV=1	Mavs	9	4	10	4	503	53.4	6.37	228607	ENSMUSG0	mmu:2286	726627.9	2567819	2913433		512942				
High	O88643	Serine/threonine-protein kinase PAK 1 OS=Mus musculus OX=10090 GN=Pak1 PE=1 SV=1	Pak1	14	5	20	3	545	60.7	5.74			O88643		3044389	2105646		1398973				
High	Q62432	Mothers against decapentaplegic homolog 2 OS=Mus musculus OX=10090 GN=Smad2 PE=1 SV=2	Smad2	6	3	18	2	467	52.2	6.58	17126	ENSMUSG0	mmu:1712	437753	1703242	3179648	4083688	2158594	3206058	2560389		
High	Q6ZWY3	40S ribosomal protein S27-like OS=Mus musculus OX=10090 GN=Rps27l PE=1 SV=3	Rps27l	38	3	15	1	84	9.5	9.45	67941	ENSMUSG0	mmu:6794									
High	P17426	AP-2 complex subunit alpha-1 OS=Mus musculus OX=10090 GN=Ap2a1 PE=1 SV=1	Ap2a1	10	9	19	2	977	107.6	7.03	11771	ENSMUSG0	mmu:1177	13505417	6610837	16941553	21747984	6918452	30966480	22127759	73664539	12048924
High	Q5EG47	5'-AMP-activated protein kinase catalytic subunit alpha-1 OS=Mus musculus OX=10090 GN=Prkaa1 PE=1 SV=2	Prkaa1	5	3	17	3	559	63.9	8.12	105787	ENSMUSG0	mmu:1057	3173966	991484	2649667	4253291	1960094	931994.4	2188799	7604306	2425249
High	Q8BJS4	SUN domain-containing protein 2 OS=Mus musculus OX=10090 GN=Sun2 PE=1 SV=3	Sun2	9	6	17	6	731	81.6	7.02	223697	ENSMUSG0	mmu:2236	12281875	2556895	11640491	8920313	2532089	25169183	3469192	25076240	10217805
High	P33146	Cadherin-15 OS=Mus musculus OX=10090 GN=Cdh15 PE=1 SV=3	Cdh15	7	5	10	5	784	85.6	4.86	12555	ENSMUSG0	B2RXU1; m			1909050	3559252	2722557				302448.8
High	Q9ES00	Ubiquitin conjugation factor E4 B OS=Mus musculus OX=10090 GN=Ube4b PE=1 SV=3	Ube4b	4	3	10	3	1173	133.2	6.07	63958	ENSMUSG0	Q6DID4; Q9		1308498	3785050		914534.3				
High	Q9DC28	Casein kinase I isoform delta OS=Mus musculus OX=10090 GN=Csnk1d PE=1 SV=2	Csnk1d	7	3	10	3	415	47.3	9.74	104318	ENSMUSG0	mmu:1043	993480.4	1988121	3620242		695495.6	2051375	1335012	1942784	576076.5
High	Q9Z1G3	V-type proton ATPase subunit C 1 OS=Mus musculus OX=10090 GN=Atp6v1c1 PE=1 SV=4	Atp6v1c1	14	8	14	8	382	43.9	7.46	66335	ENSMUSG0	mmu:6633	926966.5	585996.5	3360058	3675165	692330	3912358	2538719	11737121	6941177
High	Q9DBJ3	Brain-specific angiogenesis inhibitor 1-associated protein 2-like protein 1 OS=Mus musculus OX=10090 GN=Baiap2l1 PE=1 SV=1	Baiap2l1	12	5	11	5	514	57.2	8.75	66898	ENSMUSG0	mmu:6689	2742142	1558764	2364810	6940668	362227.8	8178410	4925671	13448912	4791670
High	Q8C9B9	Death-inducer obliterator 1 OS=Mus musculus OX=10090 GN=Dido1 PE=1 SV=4	Dido1	3	4	9	4	2256	247	7.91	23856	ENSMUSG0	A2AJ47; A2		2176735	4608506			2443722			
High	Q9DBG3	AP-2 complex subunit beta OS=Mus musculus OX=10090 GN=Ap2b1 PE=1 SV=1	Ap2b1	4	4	10	3	937	104.5	5.38	71770	ENSMUSG0	mmu:7177	4776981	9725059	22751933	44397412	9715520	20856523	70449723	34231604	12378485
High	Q8R080	G2 and S phase-expressed protein 1 OS=Mus musculus OX=10090 GN=Gtse1 PE=1 SV=2	Gtse1	11	7	11	7	741	78.7	9.39	29870	ENSMUSG0	mmu:2987		1856512	7283349	244653.2	1235880	1244412	785395.2		
High	Q5PSV9	Mediator of DNA damage checkpoint protein 1 OS=Mus musculus OX=10090 GN=Mdc1 PE=1 SV=1	Mdc1	5	5	15	5	1707	184.6	5.19	240087		mmu:2400		5338102	4738610		1741876				
High	O70551	SRSF protein kinase 1 OS=Mus musculus OX=10090 GN=Srpk1 PE=1 SV=2	Srpk1	6	4	10	4	648	73	6.19	20815	ENSMUSG0	O70193; O7		3137730	4706076		3138090	1443694			
High	Q9D0R2	Threonine--tRNA ligase 1, cytoplasmic OS=Mus musculus OX=10090 GN=Tars1 PE=1 SV=2	Tars1	11	8	14	7	722	83.3	7.36	110960	ENSMUSG0	mmu:1109	401419.7		5701088			1027155	2465715	3452777	4792420
High	O88291	DBIRD complex subunit ZNF326 OS=Mus musculus OX=10090 GN=Znf326 PE=1 SV=1	Znf326	7	3	10	3	580	65.2	5.19	54367		mmu:5436	2382262	778628.5	1818748						
High	Q8QZT1	Acetyl-CoA acetyltransferase, mitochondrial OS=Mus musculus OX=10090 GN=Acat1 PE=1 SV=1	Acat1	12	3	6	3	424	44.8	8.51	110446	ENSMUSG0	mmu:1104	644081.8	639091.9	1776169						679773.8
High	Q3UHJ0	AP2-associated protein kinase 1 OS=Mus musculus OX=10090 GN=Aak1 PE=1 SV=2	Aak1	10	6	34	6	959	103.3	6.7	269774	ENSMUSG0	B2RUJ0; m		4230491	7824584	8266274	5032941	2576620	4746055		
High	P08775	DNA-directed RNA polymerase II subunit RPB1 OS=Mus musculus OX=10090 GN=Polr2a PE=1 SV=3	Polr2a	5	6	14	6	1970	217	7.37	20020	ENSMUSG0	mmu:2002	775075.3	2661566	4415632						
High	Q8VE97	Serine/arginine-rich splicing factor 4 OS=Mus musculus OX=10090 GN=Srsf4 PE=2 SV=1	Srsf4	6	3	13	1	489	55.9	11.4			Q8VE97; Q9		1313643	713812						
High	Q63932	Dual specificity mitogen-activated protein kinase kinase 2 OS=Mus musculus OX=10090 GN=Map2k2 PE=1 SV=2	Map2k2	11	4	15	2	401	44.4	7.05	26396	ENSMUSG0	mmu:2639	829817.1	1770816	1709259			1469033		2477700	
High	Q8R010	Aminoacyl tRNA synthase complex-interacting multifunctional protein 2 OS=Mus musculus OX=10090 GN=Aimp2 PE=1 SV=2	Aimp2	15	3	11	3	320	35.4	7.83	231872	ENSMUSG0	E9QP67; m	1272683	408531.3	1262199	1146612	436328.8	2895420	1033077	4055836	1174717
High	Q99K28	ADP-ribosylation factor GTPase-activating protein 2 OS=Mus musculus OX=10090 GN=Arfgap2 PE=1 SV=1	Arfgap2	5	2	8	2	520	56.6	8.18	77038	ENSMUSG0	mmu:7703		666975.6	1125397	2726071	924376.6	1618137	3627420		
High	Q8BUV3	Gephyrin OS=Mus musculus OX=10090 GN=Gphn PE=1 SV=2	Gphn	10	8	19	8	769	83.2	5.6	268566	ENSMUSG0	E9QKJ1; m		1497331	5337995		1094486	1232960		1622672	
High	P42128	Forkhead box protein K1 OS=Mus musculus OX=10090 GN=Foxk1 PE=1 SV=2	Foxk1	7	5	11	4	719	74.9	9.17	17425	ENSMUSG0	O35939; P4	377268.6	960265.4	4273818	533925.1	1315814	1571459			495682
High	P59326	YTH domain-containing family protein 1 OS=Mus musculus OX=10090 GN=Ythdf1 PE=1 SV=1	Ythdf1	8	4	21	1	559	60.8	8.95	228994	ENSMUSG0	mmu:2289	959782.3	3588072	3441569	2077399	1264042	2202206	2439585	4546605	708276.9
High	Q91ZW3	SWI/SNF-related matrix-associated actin-dependent regulator of chromatin subfamily A member 5 OS=Mus musculus OX=10090 GN=Smarc	Smarca5	12	12	17	12	1051	121.6	8.15	93762	ENSMUSG0	mmu:9376	8853897	4356232	8020316	4502229	2599863	6728086	6784202	5720112	1094120
High	Q8N9S3	Activator of 90 kDa heat shock protein ATPase homolog 2 OS=Mus musculus OX=10090 GN=Ahsa2 PE=1 SV=2	Ahsa2	7	2	10	2	331	37.6	6.44	268390	ENSMUSG0	mmu:2683	1763662	960742.1	3932868	2348803	677325	1849866	1900750	5762563	
High	Q69ZR2	E3 ubiquitin-protein ligase HECTD1 OS=Mus musculus OX=10090 GN=Hectd1 PE=1 SV=2	Hectd1	4	10	21	10	2618	289.9	5.41		ENSMUSG0	Q69ZR2	508239	3553797	3250877		438107	269814.2	219033	3919032	247382.6
High	Q8VBV7	COP9 signalosome complex subunit 8 OS=Mus musculus OX=10090 GN=Cops8 PE=1 SV=1	Cops8	23	3	5	3	209	23.2	5.2	108679	ENSMUSG0	mmu:1086								3994889	1048422
High	P46467	Vacuolar protein sorting-associated protein 4B OS=Mus musculus OX=10090 GN=Vps4b PE=1 SV=2	Vps4b	5	2	7	2	444	49.4	7.11	20479	ENSMUSG0	mmu:2047		2242116	2748775		1122749		823835.3	3640607	1263788
High	P14211	Calreticulin OS=Mus musculus OX=10090 GN=Calr PE=1 SV=1	Calr	13	6	14	6	416	48	4.49	12317	ENSMUSG0	mmu:1231	13217870	14194366	27879904	9623983	4511280	14123771	6091351	23661193	4654822
High	O55222	Integrin-linked protein kinase OS=Mus musculus OX=10090 GN=Ilk PE=1 SV=2	Ilk	6	3	13	3	452	51.3	8.07	16202	ENSMUSG0	mmu:1620	2152783	782260.8	3574019	1750995		3527917	1108088	11385611	4765803
High	Q8BX10	Serine/threonine-protein phosphatase PGAM5, mitochondrial OS=Mus musculus OX=10090 GN=Pgam5 PE=1 SV=1	Pgam5	12	4	14	4	288	32	9.04	72542	ENSMUSG0	B2RSM6; m	2484944	1058863	2505389	1174864	461366.3	3103356	2588853	10590274	2546280
High	O54931	A-kinase anchor protein 2 OS=Mus musculus OX=10090 GN=Akap2 PE=1 SV=3		6	7	21	7	893	98.5	5.21				767283.9	3974591	7299249	7335402	5346068	5835345	4632809	6532847	4603833
High	P15116	Cadherin-2 OS=Mus musculus OX=10090 GN=Cdh2 PE=1 SV=2	Cdh2	9	4	9	4	906	99.7	4.78	12558	ENSMUSG0	mmu:1255	1148071	893235.2	2935259	1531073	2822123	1524853	2682079	1551606	
High	Q9D662	Protein transport protein Sec23B OS=Mus musculus OX=10090 GN=Sec23b PE=1 SV=1	Sec23b	4	2	8	2	767	86.4	6.96	27054	ENSMUSG0	A2ANA1; m			1071527	1427837	795984.1	1039080	230500.9		
High	P54823	Probable ATP-dependent RNA helicase DDX6 OS=Mus musculus OX=10090 GN=Ddx6 PE=1 SV=1	Ddx6	11	4	14	4	483	54.2	8.66	13209	ENSMUSG0	mmu:1320	1772674	2684453	6937352	7169277	1734042	6245299	1581039		
High	P21300	Aldo-keto reductase family 1 member B7 OS=Mus musculus OX=10090 GN=Akr1b7 PE=1 SV=4	Akr1b7	4	2	10	2	316	36	7.25	11997	ENSMUSG0	mmu:1199		793340.3	2096127	2674163		2875731	2932439	1227571	
High	P70122	Ribosome maturation protein SBDS OS=Mus musculus OX=10090 GN=Sbds PE=1 SV=4	Sbds	18	5	14	5	250	28.8	8.76	66711	ENSMUSG0	mmu:6671	1228697		994950.2	113203.3	79983.07	2590347		9406552	3999075
High	Q80VD1	Protein FAM98B OS=Mus musculus OX=10090 GN=Fam98b PE=1 SV=1	Fam98b	5	2	5	2	429	45.3	8.5	68215	ENSMUSG0	A2ATZ4; m		504957.5	726563.4						
High	Q8BIH0	Histone deacetylase complex subunit SAP130 OS=Mus musculus OX=10090 GN=Sap130 PE=1 SV=2	Sap130	3	3	11	3	1057	111.2	9.91	269003	ENSMUSG0	mmu:2690		2388553	2915115	1750045	3728650	1424680	1984932	6744315	
High	Q8CFI0	E3 ubiquitin-protein ligase NEDD4-like OS=Mus musculus OX=10090 GN=Nedd4l PE=1 SV=2	Nedd4l	6	5	19	3	1004	115.3	6.15		ENSMUSG0	Q8BRT9; Q8	679052.3	6906917	4090735	1763167	3145721	3293128	3671815	4278195	
High	P33174	Chromosome-associated kinesin KIF4 OS=Mus musculus OX=10090 GN=Kif4 PE=1 SV=3	Kif4	4	5	10	5	1231	139.4	6.68	16571	ENSMUSG0	mmu:1657	1343690	296398.2	4389306		386460.7				288581.3
High	Q3UPH7	Rho guanine nucleotide exchange factor 40 OS=Mus musculus OX=10090 GN=Arhgef40 PE=1 SV=1	Arhgef40	4	5	9	5	1517	165	5.55	268739	ENSMUSG0	mmu:2687	415568.1	3764600	5759668		2495027	1119197			
High	P39061	Collagen alpha-1(XVIII) chain OS=Mus musculus OX=10090 GN=Col18a1 PE=1 SV=4	Col18a1	5	7	8	7	1774	182.1	5.62	12822	ENSMUSG0	mmu:1282	5759141	2217936	6453304	1863197	1048482	3471658	1219255	8086815	6291418
High	Q01730	Ras suppressor protein 1 OS=Mus musculus OX=10090 GN=Rsu1 PE=1 SV=3	Rsu1	21	4	7	4	277	31.5	8.88			Q01730								3965291	2487049
High	Q8BKS9	Pumilio homolog 3 OS=Mus musculus OX=10090 GN=Pum3 PE=1 SV=2	Pum3	4	3	5	3	647	72.8	9.66	52874		Q6A0E6; Q8	1104761	755482.3	3152857		731132.2	1442380			
High	Q9DBE9	pre-rRNA 2'-O-ribose RNA methyltransferase FTSJ3 OS=Mus musculus OX=10090 GN=Ftsj3 PE=1 SV=1	Ftsj3	7	6	17	6	838	95.5	8.38	56095	ENSMUSG0	mmu:5609	2930106		3482072		540581.3	1300520			405881
High	O35639	Annexin A3 OS=Mus musculus OX=10090 GN=Anxa3 PE=1 SV=4	Anxa3	13	4	9	3	323	36.4	5.76	11745	ENSMUSG0	mmu:1174	308466.3	822454.8	2538922	1487567	356295.5	1325536	949646.9		
High	Q9CQD1	Ras-related protein Rab-5A OS=Mus musculus OX=10090 GN=Rab5a PE=1 SV=1	Rab5a	23	4	12	3	215	23.6	8.15	271457	ENSMUSG0	mmu:2714	1711651	670092.1	518024.1	307026.9		360580.3		7439784	1845619
High	Q99LD8	N(G),N(G)-dimethylarginine dimethylaminohydrolase 2 OS=Mus musculus OX=10090 GN=Ddah2 PE=1 SV=1	Ddah2	15	4	6	4	285	29.6	6.01	51793	ENSMUSG0	mmu:5179	517517.8	833412.6	2675712					1744268	3004156
High	Q56A08	G-patch domain and KOW motifs-containing protein OS=Mus musculus OX=10090 GN=Gpkow PE=1 SV=2	Gpkow	5	2	7	2	488	53.8	8.07	209416	ENSMUSG0	A0JLW7; A2		1023808	1945364		864424.3				
High	Q8R3N6	THO complex subunit 1 OS=Mus musculus OX=10090 GN=Thoc1 PE=1 SV=1	Thoc1	4	2	7	2	657	75.4	4.97	225160	ENSMUSG0	mmu:2251		2000030	2362444	2470564	2275836	2197888	1838273		
High	Q80UM3	N-alpha-acetyltransferase 15, NatA auxiliary subunit OS=Mus musculus OX=10090 GN=Naa15 PE=1 SV=1	Naa15	11	12	24	12	865	100.9	7.62			Q80UM3; Q	4499017	678104.8	5000403			1717444		6053492	1707956
High	Q920B9	FACT complex subunit SPT16 OS=Mus musculus OX=10090 GN=Supt16h PE=1 SV=2	Supt16h	2	3	13	3	1047	119.7	5.66	114741		mmu:1147	9856703	4172990	16724614	3437827	1362504	3291389	3406460	15049155	5180273
High	Q4FZC9	Nesprin-3 OS=Mus musculus OX=10090 GN=Syne3 PE=1 SV=1	Syne3	6	5	7	5	975	112	6.33	212073	ENSMUSG0	Q4FZC9; Q8	644759.8								
High	Q6PDI5	Proteasome adapter and scaffold protein ECM29 OS=Mus musculus OX=10090 GN=Ecpas PE=1 SV=3		6	9	20	9	1840	203.6	7.06				1612830	2458952	10142190	180611.7	451411.7	1743945	1558958	7590835	1636666

High	Q9CZ13	Cytochrome b-c1 complex subunit 1, mitochondrial OS=Mus musculus OX=10090 GN=Uqcrc1 PE=1 SV=2	Uqcrc1	11	5	13	4	480	52.8	6.21	22273	ENSMUSG0	mmu:2227	246168.7								3035387
High	Q9D892	Inosine triphosphate pyrophosphatase OS=Mus musculus OX=10090 GN=Itpa PE=1 SV=2	Itpa	15	4	10	4	198	21.9	5.87	16434	ENSMUSG0	mmu:1643	920589.2	784222.9	2330514		511627.4	4543923	1547647	19325848	10104672
High	Q02257	Junction plakoglobin OS=Mus musculus OX=10090 GN=Jup PE=1 SV=3	Jup	9	6	18	5	745	81.7	6.14	16480	ENSMUSG0	mmu:1648	3878942		798758.4	11409026		4261746	4544344	10915888	3934466
High	Q8BRT1	CLIP-associating protein 2 OS=Mus musculus OX=10090 GN=Clasp2 PE=1 SV=1	Clasp2	6	8	15	6	1286	140.7	8.63	76499		mmu:7649	3887056	1388142	8633620	4629836	348231.6	7298146	1652539	12132719	
High	Q8BP27	Swi5-dependent recombination DNA repair protein 1 homolog OS=Mus musculus OX=10090 GN=Sfr1 PE=1 SV=2	Sfr1	11	4	10	4	319	35.2	5.15	67788	ENSMUSG0	mmu:6778	21912391	4864835	5713514	1533543	1972881	3590105	1544674	10102406	8327545
High	Q91YU6	Leucine zipper putative tumor suppressor 2 OS=Mus musculus OX=10090 GN=Lzts2 PE=1 SV=3	Lzts2	7	3	5	3	671	72.5	7.11	226154	ENSMUSG0	E9QKK6; m		642781.7	859500.4		1241166				
High	P62880	Guanine nucleotide-binding protein G(I)/G(S)/G(T) subunit beta-2 OS=Mus musculus OX=10090 GN=Gnb2 PE=1 SV=3	Gnb2	8	3	5	2	340	37.3	6	14693	ENSMUSG0	mmu:1469						1532198	1199154	3790819	2358168
High	P37913	DNA ligase 1 OS=Mus musculus OX=10090 GN=Lig1 PE=1 SV=2	Lig1	4	4	9	4	916	102.2	6.81	16881		mmu:1688	485882.6		1585802		630260.8	1734523	637688.5	1110630	
High	E9Q1P8	Interferon regulatory factor 2-binding protein 2 OS=Mus musculus OX=10090 GN=Irf2bp2 PE=1 SV=1	Irf2bp2	9	4	11	4	570	59.3	8.69	270110	ENSMUSG0	E9Q1P8; m		3484888	2631179	1843952	1599605	1422648			
High	O88448	Kinesin light chain 2 OS=Mus musculus OX=10090 GN=Klc2 PE=1 SV=1	Klc2	8	5	10	3	599	66.6	7.21			O88448			1883669	4672761	591822.8		5779990	9578511	5942732
High	O08528	Hexokinase-2 OS=Mus musculus OX=10090 GN=Hk2 PE=1 SV=1	Hk2	10	9	27	6	917	102.5	6.11	15277	ENSMUSG0	mmu:1527	404521.3			1232712	604705.5	946734.8	2217535	1357672	1096177
High	Q7TSH3	Zinc finger protein 516 OS=Mus musculus OX=10090 GN=Znf516 PE=1 SV=1	Znf516	3	3	5	3	1157	124.7	8.51	329003	ENSMUSG0	mmu:3290	955653.4	838772.4	1687557		650729.6			305976	128712.9
High	Q8BP47	Asparagine--tRNA ligase, cytoplasmic OS=Mus musculus OX=10090 GN=NARS1 PE=1 SV=2	Nars1	18	7	16	7	559	64.2	5.86	70223	ENSMUSG0	mmu:7022	3628398	973221.6	3065514		513628.6	3511086	2286712	7493528	2596035
High	Q7TQI3	Ubiquitin thioesterase OTUB1 OS=Mus musculus OX=10090 GN=Otub1 PE=1 SV=2	Otub1	20	4	9	4	271	31.3	4.94	107260	ENSMUSG0	mmu:1072			648014.1			1597803		6708525	3337399
High	P47758	Signal recognition particle receptor subunit beta OS=Mus musculus OX=10090 GN=Srprb PE=1 SV=1	Srprb	15	4	15	4	269	29.6	9.28	20818	ENSMUSG0	mmu:2081	611166.1	1289949	1780431		814850.7	2027868			
High	Q8BK64	Activator of 90 kDa heat shock protein ATPase homolog 1 OS=Mus musculus OX=10090 GN=Ahsa1 PE=1 SV=2	Ahsa1	16	6	17	6	338	38.1	5.53	217737	ENSMUSG0	mmu:2177	1524862	1006439	2230588		1250904	1902979			2074221
High	Q61316	Heat shock 70 kDa protein 4 OS=Mus musculus OX=10090 GN=Hspa4 PE=1 SV=1	Hspa4	5	4	9	3	841	94.1	5.24			Q61316	256882.6	864037.1	728024.6						1109433
High	B9EJR8	Dynein axonemal assembly factor 5 OS=Mus musculus OX=10090 GN=Dnaaf5 PE=1 SV=1	Dnaaf5	5	4	12	4	853	93.8	6.15	433956	ENSMUSG0	B9EJR8; mn	1469210	880760.2	6422181	3031814	1147038	3930305		3516772	1906250
High	O35345	Importin subunit alpha-7 OS=Mus musculus OX=10090 GN=Kpna6 PE=1 SV=2	Kpna6	12	6	10	1	536	59.9	4.94	16650	ENSMUSG0	mmu:1665	328954.1		377340.4			997036.9	895552.5	4226474	1076590
High	Q9R0I7	YLP motif-containing protein 1 OS=Mus musculus OX=10090 GN=Ylpm1 PE=2 SV=2	Ylpm1	2	2	4	2	1386	155	6.65		ENSMUSG0	E9QKY3; Q7		793681.1							
High	Q8R361	Rab11 family-interacting protein 5 OS=Mus musculus OX=10090 GN=Rab11fip5 PE=1 SV=2	Rab11fip5	4	2	8	2	645	69.5	9.07	52055	ENSMUSG0	C4IXU4; mr		551489.9	1148864		1124475	920011.8	1305004		
High	P37889	Fibulin-2 OS=Mus musculus OX=10090 GN=Fbln2 PE=1 SV=2	Fbln2	3	3	13	3	1221	131.7	4.68	14115	ENSMUSG0	G5E8B3; m	913845.8	758140	1046790				2685687		
High	Q9WV92	Band 4.1-like protein 3 OS=Mus musculus OX=10090 GN=Epb41l3 PE=1 SV=1	Epb41l3	5	4	15	3	929	103.3	5.31	13823	ENSMUSG0	mmu:1382	414724.3	326055.1	2683465		528065.6	1093588			
High	Q91VH2	Sorting nexin-9 OS=Mus musculus OX=10090 GN=Snx9 PE=1 SV=1	Snx9	4	2	8	2	595	66.5	5.52	66616	ENSMUSG0	mmu:6661	998213.3	681184.2	1688528	693570.3				2469339	757040.7
High	Q3THK7	GMP synthase [glutamine-hydrolyzing] OS=Mus musculus OX=10090 GN=Gmps PE=1 SV=2	Gmps	9	6	17	6	693	76.7	6.73	229363	ENSMUSG0	mmu:2293	2438748	2194645	6404491	1697783	452834.8	3932614	2602840	12778776	3608124
High	Q8WTY4	Anamorsin OS=Mus musculus OX=10090 GN=Ciapin1 PE=1 SV=1	Ciapin1	9	2	12	2	309	33.4	5.2	109006	ENSMUSG0	mmu:1090	8548143	3048327	6338862	805017.9	548299.1	2576928		8594462	1160012
High	Q8K1A6	Coiled-coil and C2 domain-containing protein 1A OS=Mus musculus OX=10090 GN=Cc2d1a PE=1 SV=2	Cc2d1a	2	2	4	2	943	103.6	7.84	212139	ENSMUSG0	mmu:2121			1596047					2031524	
High	P62858	40S ribosomal protein S28 OS=Mus musculus OX=10090 GN=Rps28 PE=1 SV=1	Rps28	29	2	7	2	69	7.8	10.7	54127	ENSMUSG0	mmu:5412	3294824	2377498	9809756	6387851	3647909	5735270	10163597	9625008	2211110
High	P47856	Glutamine--fructose-6-phosphate aminotransferase [isomerizing] 1 OS=Mus musculus OX=10090 GN=Gfpt1 PE=1 SV=3	Gfpt1	3	2	5	2	697	78.5	6.84	14583	ENSMUSG0	mmu:1458	1394065	412425.7	2460928	980127.8		4593950	1974306		
High	Q3UCQ1	Forkhead box protein K2 OS=Mus musculus OX=10090 GN=Foxk2 PE=1 SV=3	Foxk2	10	5	10	4	651	68.4	9.51	68837	ENSMUSG0	A2AN27; B5		1904274	2671547	1064292	1480971	616666.8		1448044	
High	Q6P9Q4	FH1/FH2 domain-containing protein 1 OS=Mus musculus OX=10090 GN=Fhod1 PE=1 SV=3	Fhod1	4	4	11	4	1197	129.5	6.28	234686	ENSMUSG0	mmu:2346	377924.3	1017502	2503944	1213903	3760395	1700778	2386870	1434640	358265.7
High	P56135	ATP synthase subunit f, mitochondrial OS=Mus musculus OX=10090 GN=Atp5mf PE=1 SV=3	Atp5mf	27	3	10	3	88	10.3	9.95	57423	ENSMUSG0	mmu:5742	343074	642197.6	966084.9	2771082	607473.2	2898540	5112221	9812630	5293850
High	Q9R190	Metastasis-associated protein MTA2 OS=Mus musculus OX=10090 GN=Mta2 PE=1 SV=1	Mta2	10	5	13	4	668	75	9.67	23942	ENSMUSG0	mmu:2394	2386939	1391427	4245663			1582428		3352339	438525.7
High	P59759	Myocardin-related transcription factor B OS=Mus musculus OX=10090 GN=Mrtfb PE=1 SV=1	Mrtfb	4	3	9	2	1080	117.5	6.16	239719		P59759		1006483	2659480		869902.9			3643075	
High	Q9CSN1	SNW domain-containing protein 1 OS=Mus musculus OX=10090 GN=Snw1 PE=1 SV=3	Snw1	17	7	12	7	536	61.4	9.48	66354		mmu:6635		4141153	2346039		734476.6				
High	Q9JM58	Cytokine receptor-like factor 1 OS=Mus musculus OX=10090 GN=Crlf1 PE=1 SV=1	Crlf1	6	2	3	2	425	46.6	8.94	12931	ENSMUSG0	mmu:1293	1512439								
High	Q9WUA3	ATP-dependent 6-phosphofructokinase, platelet type OS=Mus musculus OX=10090 GN=Pfkp PE=1 SV=1	Pfkp	4	3	6	2	784	85.4	7.11	56421	ENSMUSG0	mmu:5642									
High	Q62376	U1 small nuclear ribonucleoprotein 70 kDa OS=Mus musculus OX=10090 GN=Snrnp70 PE=1 SV=2	Snrnp70	8	4	20	4	448	52	9.94	20637	ENSMUSG0	mmu:2063	1346167	2461246	5566846		3826053	2842144	2544543	7411267	2629475
High	O54734	Dolichyl-diphosphooligosaccharide--protein glycosyltransferase 48 kDa subunit OS=Mus musculus OX=10090 GN=Ddost PE=1 SV=2	Ddost	5	2	7	2	441	49	5.83	13200	ENSMUSG0	mmu:1320	1022109	489259.7	1009995	1829702		3051565			1545597
High	Q6P9J9	Anoctamin-6 OS=Mus musculus OX=10090 GN=Ano6 PE=1 SV=1	Ano6	2	2	11	2	911	106.2	6.76	105722	ENSMUSG0	mmu:1057	1700809	1670751	2529746	4173918	1617164	3399067	2371070	4073710	1682781
High	Q9DCT2	NADH dehydrogenase [ubiquinone] iron-sulfur protein 3, mitochondrial OS=Mus musculus OX=10090 GN=Ndufs3 PE=1 SV=2	Ndufs3	10	2	3	2	263	30.1	7.17	68349	ENSMUSG0	mmu:6834					353230			2121993	3186224
High	Q9JLI8	Squamous cell carcinoma antigen recognized by T-cells 3 OS=Mus musculus OX=10090 GN=Sart3 PE=1 SV=1	Sart3	3	3	9	3	962	109.6	5.24	53890	ENSMUSG0	mmu:5389	1004696	560369.6	3236891	2910092	1783259	4565015	2561894	10737110	4164145
High	P10833	Ras-related protein R-Ras OS=Mus musculus OX=10090 GN=Rras PE=1 SV=1	Rras	12	2	4	1	218	23.7	6.79	20130	ENSMUSG0	mmu:2013		70177.38		1259071	855462.8	681576.8		360635.6	139802.3
High	P17427	AP-2 complex subunit alpha-2 OS=Mus musculus OX=10090 GN=Ap2a2 PE=1 SV=2	Ap2a2	9	8	17	1	938	104	6.93	11772	ENSMUSG0	mmu:1177									
High	E9QAT4	Protein transport protein Sec16A OS=Mus musculus OX=10090 GN=Sec16a PE=1 SV=1	Sec16a	3	4	13	4	2357	254	5.81	227648	ENSMUSG0	A2AIX1; E9	1796214	2911364	2559017	2494484	790628.7	2523443	4541134	3951901	1713343
High	Q9D903	Probable rRNA-processing protein EBP2 OS=Mus musculus OX=10090 GN=Ebna1bp2 PE=2 SV=1	Ebna1bp2	19	6	15	6	306	34.7	10.08	69072	ENSMUSG0	A2ACZ0; mr	3581427	1567927	4959326	16169877	1821226	8823668	12000459	18383892	2987939
High	P99029	Peroxiredoxin-5, mitochondrial OS=Mus musculus OX=10090 GN=Prdx5 PE=1 SV=2	Prdx5	25	3	4	3	210	21.9	8.85	54683	ENSMUSG0	mmu:5468	123992.6					890832.8	1013721		4288244
High	Q3TCJ1	BRISC complex subunit Abraxas 2 OS=Mus musculus OX=10090 GN=Abraxas2 PE=1 SV=1	Abraxas2	2	2	8	2	415	46.9	6.18	109359		mmu:1093									
High	O35643	AP-1 complex subunit beta-1 OS=Mus musculus OX=10090 GN=Ap1b1 PE=1 SV=2	Ap1b1	5	3	18	2	943	103.9	5.17	11764	ENSMUSG0	mmu:1176			1303149	2178255	1004785	2575869	1237550		
High	Q9CR68	Cytochrome b-c1 complex subunit Rieske, mitochondrial OS=Mus musculus OX=10090 GN=Uqcrfs1 PE=1 SV=1	Uqcrfs1	17	5	9	5	274	29.3	8.7	66694	ENSMUSG0	mmu:6669	10539032	2823089	5760000		623836.4	3956958	6103408	20260967	16416147
High	Q99PP7	E3 ubiquitin-protein ligase TRIM33 OS=Mus musculus OX=10090 GN=Trim33 PE=1 SV=2	Trim33	2	2	3	2	1142	123.8	6.74	94093		mmu:9409	309956.3		1752416						
High	Q60847	Collagen alpha-1(XII) chain OS=Mus musculus OX=10090 GN=Col12a1 PE=2 SV=3	Col12a1	2	6	10	6	3120	340	5.64	12816	ENSMUSG0	mmu:1281	1821311		1102437	2328301	98500.06	1872265	579640.1	740788.9	699636.7
High	E1U8D0	Protein SOGA1 OS=Mus musculus OX=10090 GN=Soga1 PE=1 SV=3		3	3	5	3	1418	159.1	6.46												
High	Q6ZPZ3	Zinc finger CCCH domain-containing protein 4 OS=Mus musculus OX=10090 GN=Zc3h4 PE=1 SV=2	Zc3h4	5	5	9	5	1304	140.9	6.27	330474		mmu:3304									
High	Q9CY64	Biliverdin reductase A OS=Mus musculus OX=10090 GN=Blvra PE=1 SV=1	Blvra	9	3	4	3	295	33.5	7.02	109778	ENSMUSG0	mmu:1097	331937.4			671610.6			801513.9	1160622	866194.3
High	Q9CYT6	Adenylyl cyclase-associated protein 2 OS=Mus musculus OX=10090 GN=Cap2 PE=1 SV=1	Cap2	8	4	5	3	476	52.8	6.43	67252	ENSMUSG0	mmu:6725							3080336		
High	Q8BJ71	Nuclear pore complex protein Nup93 OS=Mus musculus OX=10090 GN=Nup93 PE=1 SV=1	Nup93	6	6	13	6	819	93.2	5.72	71805	ENSMUSG0	mmu:7180	744927.2	1381977	4966777	2865847	1541503	2864437	1958029	2245112	617666.6
High	Q3UMF0-2	Isoform 2 of Cordon-bleu protein-like 1 OS=Mus musculus OX=10090 GN=Cobll1	Cobll1	10	9	11	9	1241	133.3	7.36			Q3UMF0-2	566587.5		5126051	4358.108	616615.8	3876.021	16627.93	1318994	663682.3
High	Q8CGC6	RNA-binding protein 28 OS=Mus musculus OX=10090 GN=Rbm28 PE=1 SV=4	Rbm28	3	2	6	2	750	84.2	9.54	68272	ENSMUSG0	E9QKF8; mr									
High	Q69Z38	Inactive tyrosine-protein kinase PEAK1 OS=Mus musculus OX=10090 GN=Peak1 PE=1 SV=4	Peak1	5	5	9	5	1735	191	6.87	244895	ENSMUSG0	E9QKY2; mr		1377000							
High	Q99MJ9	ATP-dependent RNA helicase DDX50 OS=Mus musculus OX=10090 GN=Ddx50 PE=2 SV=1	Ddx50	4	3	6	2	734	82.1	9.25	94213	ENSMUSG0	mmu:9421	1043013		1883315						
High	O08599	Syntaxin-binding protein 1 OS=Mus musculus OX=10090 GN=Stxbp1 PE=1 SV=2	Stxbp1	4	2	5	2	594	67.5	6.96	20910	ENSMUSG0	A2ARS2; A2									
High	Q68FF6	ARF GTPase-activating protein GIT1 OS=Mus musculus OX=10090 GN=Git1 PE=1 SV=1	Git1	6	4	8	4	770	85.2	6.93	216963	ENSMUSG0	Q68FF6			1266791		1034518	1690631		4560867	460065.3
High	Q6A0A2	La-related protein 4B OS=Mus musculus OX=10090 GN=Larp4b PE=1 SV=2	Larp4b	7	4	15	4	741	81.6	7.49	217980	ENSMUSG0	mmu:2179	12647115	7875949	25276584	5023231	1548710	3673329	9361258	15847768	2098505
High	Q2TPA8	Hydroxysteroid dehydrogenase-like protein 2 OS=Mus musculus OX=10090 GN=Hsdl2 PE=1 SV=1	Hsdl2	4	2	12	2	490	54.2	6.74	72479	ENSMUSG0	mmu:7247		2164429	3377084	8723076	4054112	9561421	4172424	2046846	1490619
High	Q5I1X5	RelA-associated inhibitor OS=Mus musculus OX=10090 GN=Ppp1r13l PE=1 SV=1	Ppp1r13l	3	2	6	2	824	88.9	6.86	333654	ENSMUSG0	mmu:3336	457184.6		1463051	1724208	249408.6	817844.5	901377.8	1778336	1202986
High	Q8BJH1	Zinc finger C2HC domain-containing protein 1A OS=Mus musculus OX=10090 GN=Zc2hc1a PE=1 SV=1	Zc2hc1a	9	3	7	3	324	35.1	9.88	67306	ENSMUSG0	mmu:6730		1349103	1822599	2025565	996439.6	1140912	2655505		
High	Q60668	Heterogeneous nuclear ribonucleoprotein D0 OS=Mus musculus OX=10090 GN=Hnrnpd PE=1 SV=2	Hnrnpd	7	3	8	2	355	38.3	7.81	11991	ENSMUSG0	mmu:1199		615915.7	2647057				4939626	3026518	1558781
High	P14152	Malate dehydrogenase, cytoplasmic OS=Mus musculus OX=10090 GN=Mdh1 PE=1 SV=3	Mdh1	12	3	4	3	334	36.5	6.58	17449	ENSMUSG0	mmu:1744	842370.1		328051.1	1197439		1212704	2843853	2870598	7437996
High	Q91YD9	Actin nucleation-promoting factor WASL OS=Mus musculus OX=10090 GN=Wasl PE=1 SV=1	Wasl	9	3	4	3	501	54.2	7.93	73178	ENSMUSG0	mmu:7317		1814841	3528581		1202001	3481922			
High	Q9WVE8	Protein kinase C and casein kinase substrate in neurons protein 2 OS=Mus musculus OX=10090 GN=Pacsin2 PE=1 SV=1	Pacsin2	7	4	6	4	486	55.8	5.2	23970	ENSMUSG0	mmu:2397	266222.7	765538.9	2053012	1609263		1338829		2742820	3082045
High	Q5SUE8	Ankyrin repeat domain-containing protein 40 OS=Mus musculus OX=10090 GN=Ankrd40 PE=1 SV=1	Ankrd40	10	3	3	3	363	40.5	5.1	71452	ENSMUSG0	mmu:7145		2316527	2098984						
High	P61222	ATP-binding cassette sub-family E member 1 OS=Mus musculus OX=10090 GN=Abce1 PE=1 SV=1	Abce1	6	3	5	3	599	67.3	8.34	24015	ENSMUSG0	mmu:2401	2961818		953851.3	1330901		882563.3			841064.1
High	E9Q557	Desmoplakin OS=Mus musculus OX=10090 GN=Dsp PE=1 SV=1	Dsp	2	5	6	5	2883	332.7	6.8	109620	ENSMUSG0	E9Q557; m	1534617	898062.5	3774844			882225.6	1067532		
High	Q8BUK6	Protein Hook homolog 3 OS=Mus musculus OX=10090 GN=Hook3 PE=1 SV=2	Hook3	6	4	13	4	718	83.2	5.19	320191	ENSMUSG0	mmu:3201		1185975	3781110	5518109	2383919	8286211	3006486		3254320
High	Q8K363	ATP-dependent RNA helicase DDX18 OS=Mus musculus OX=10090 GN=Ddx18 PE=1 SV=1	Ddx18	7	5	6	5	660	74.1	9.52	66942	ENSMUSG0	mmu:6694									
High	Q80YR5	Scaffold attachment factor B2 OS=Mus musculus OX=10090 GN=Safb2 PE=1 SV=2	Safb2	5	4	4	1	991	111.8	6.38	224902	ENSMUSG0	mmu:2249			1694489			1082093			
High	P28658	Ataxin-10 OS=Mus musculus OX=10090 GN=Atxn10 PE=1 SV=2	Atxn10	9	5	7	5	475	53.7	5.25	54138	ENSMUSG0	mmu:5413									905935.3
High	A2AMM0	Caveolae-associated protein 4 OS=Mus musculus OX=10090 GN=Cavin4 PE=1 SV=1	Cavin4	10	4	10	4	362	41	8.65	68016	ENSMUSG0	A2AMM0; B	115415.1			2365471			1431243	5448815	4319777
High	D3YZP9	Coiled-coil domain-containing protein 6 OS=Mus musculus OX=10090 GN=Ccdc6 PE=1 SV=1	Ccdc6	4	2	4	2	469	52.9	7.34	76551	ENSMUSG0	D3YZP9; mr	2510241	847806	2624315	3508273	782838	3610017	2441743	3367453	
High	P61021	Ras-related protein Rab-5B OS=Mus musculus OX=10090 GN=Rab5b PE=1 SV=1	Rab5b	11	2	8	1	215	23.7	8.13	19344	ENSMUSG0	mmu:1934			1496980	1597923	538694.2	1292155			
High	Q8CGF7	Transcription elongation regulator 1 OS=Mus musculus OX=10090 GN=Tcerg1 PE=1 SV=2	Tcerg1	3	3	8	3	1100	123.7	8.65	56070	ENSMUSG0	mmu:5607		398586.1	1838838	1706869	616401.7	1145558			
High	Q9D2M8	Ubiquitin-conjugating enzyme E2 variant 2 OS=Mus musculus OX=10090 GN=Ube2v2 PE=1 SV=4	Ube2v2	28	4	13	1	145	16.4	8.09	70620	ENSMUSG0	A6X924; mr									
High	Q8R3Y8	Interferon regulatory factor 2-binding protein 1 OS=Mus musculus OX=10090 GN=Irf2bp1 PE=1 SV=2	Irf2bp1	4	2	8	2	584	61.7	8.18	272359	ENSMUSG0	Q8BJC9; Q8	203911.3	1910544	3513536	1960365	562731.8	3997237	977944.5	1697695	517400.5
High	Q8CCF0	U4/U6 small nuclear ribonucleoprotein Prp31 OS=Mus musculus OX=10090 GN=Prpf31 PE=1 SV=3	Prpf31	5	4	13	4	499	55.4	5.67	68988	ENSMUSG0	E9QPM6; m	3230179		2479422	573620	1270960	976876.3	659000.7	1163865	
High	Q99PL5	Ribosome-binding protein 1 OS=Mus musculus OX=10090 GN=Rrbp1 PE=1 SV=2	Rrbp1	9	10	26	10	1605	172.8	9.33	81910	ENSMUSG0	A2AVJ8; mr	2162650	2910187	9846572	4388921	2260805	6834641	7494324	10774966	2809208
High	Q93092	Transaldolase OS=Mus musculus OX=10090 GN=Taldo1 PE=1 SV=2	Taldo1	14	5	16	5	337	37.4	7.03	21351	ENSMUSG0	mmu:2135	382351.9		142937				200823.3	828852	2191954
High	Q9R0L6	Pericentriolar material 1 protein OS=Mus musculus OX=10090 GN=Pcm1 PE=1 SV=2	Pcm1	3	5	13	5	2025	228.7	5.01	18536	ENSMUSG0	E9QLK2; mr	528941.8	3960238	2696505	1925131	1239944	2362406			
High	Q9R1T2	SUMO-activating enzyme subunit 1 OS=Mus musculus OX=10090 GN=Sae1 PE=1 SV=1	Sae1	9	3	5	3	350	38.6	5.36	56459	ENSMUSG0	mmu:5645	854681.1			1489092		1279680	1664460	1953680	593270.4
High	P31938	Dual specificity mitogen-activated protein kinase kinase 1 OS=Mus musculus OX=10090 GN=Map2k1 PE=1 SV=2	Map2k1	14	5	14	3	393	43.4	6.7	26395	ENSMUSG0	mmu:2639	2375468	1629341	4476696	1368483	612267.8	1997071	1116078	14066722	3080991
High	Q922S8	Kinesin-like protein KIF2C OS=Mus musculus OX=10090 GN=Kif2c PE=1 SV=1	Kif2c	5	3	3	2	721	81	7.97	73804	ENSMUSG0	A2AE71; mr	196302.4	274463.4	2974291			240536			
High	Q9CYL5	Golgi-associated plant pathogenesis-related protein 1 OS=Mus musculus OX=10090 GN=Glipr2 PE=1 SV=3	Glipr2	17	2	4	2	154	17.1	9.51	384009	ENSMUSG0	mmu:3840		932133.9	2523708			1056759			
High	Q8C156	Condensin complex subunit 2 OS=Mus musculus OX=10090 GN=Ncaph PE=1 SV=1	Ncaph	2	2	9	2	731	82.3	4.96	215387	ENSMUSG0	mmu:2153	1131798	1853383	6244790		590552.5	4886090			
High	Q8JZX4	Splicing factor 45 OS=Mus musculus OX=10090 GN=Rbm17 PE=1 SV=1	Rbm17	6	2	8	2	405	45.3	5.82	76938	ENSMUSG0	A2AP41; mr		1393348	1295525					3909445	883926.3
High	Q9EQQ9	Protein O-GlcNAcase OS=Mus musculus OX=10090 GN=Oga PE=1 SV=2	Oga	5	3	5	3	916	103.1	4.92	76055	ENSMUSG0	mmu:7605									
High	Q8VHE0	Translocation protein SEC63 homolog OS=Mus musculus OX=10090 GN=Sec63 PE=1 SV=4	Sec63	3	2	4	2	760	87.8	5.38	140740	ENSMUSG0	E9QKG1; m	1112590	235911.2	945039.7	835274.7	404520.8				
High	O35711	Liprin-beta-2 OS=Mus musculus OX=10090 GN=Ppfibp2 PE=1 SV=3	Ppfibp2	5	2	7	1	882	98.7	6.33	19024	ENSMUSG0	mmu:1902	970369.3		2720555	3249117	1057839	1917255			
High	Q8R1N4	NudC domain-containing protein 3 OS=Mus musculus OX=10090 GN=Nudcd3 PE=1 SV=3	Nudcd3	9	2	4	2	363	40.9	5.26	209586	ENSMUSG0	mmu:2095		1604573	1565764				1795685		
High	Q9JJ28	Protein flightless-1 homolog OS=Mus musculus OX=10090 GN=Flii PE=1 SV=1	Flii	3	2	4	2	1271	144.7	6.06	14248	ENSMUSG0	mmu:1424	290661.7		892089.1	2522157					

High	Q3TWW8	Serine/arginine-rich splicing factor 6 OS=Mus musculus OX=10090 GN=Srsf6 PE=1 SV=1	Srsf6	14	6	26	4	339	39	11.46	67996	ENSMUSG0	mmu:6799	985201.1	7398086	14133449		1029629	4828639	1674085	11569140	6935967
High	Q9CXT8	Mitochondrial-processing peptidase subunit beta OS=Mus musculus OX=10090 GN=Pmpcb PE=1 SV=1	Pmpcb	10	5	14	4	489	54.6	6.99	73078	ENSMUSG0	mmu:7307	819288.1		2568336	3985927		6255064	1581497	10420137	7772375
High	Q64213	Splicing factor 1 OS=Mus musculus OX=10090 GN=Sf1 PE=1 SV=6	Sf1	8	4	13	4	653	70.4	8.98		ENSMUSG0	E9QK02; O0		2150526	2448405	1263790	2044629	1438954	1370634		
High	Q3TXS7	26S proteasome non-ATPase regulatory subunit 1 OS=Mus musculus OX=10090 GN=Psmd1 PE=1 SV=1	Psmd1	3	3	10	3	953	105.7	5.39	70247	ENSMUSG0	B2RRP8; m	588898.7	316758.2	3081350		1265923	4170266			1123074
High	Q8BUN5	Mothers against decapentaplegic homolog 3 OS=Mus musculus OX=10090 GN=Smad3 PE=1 SV=2	Smad3	6	2	7	1	425	48.1	7.15	17127	ENSMUSG0	mmu:1712									
High	Q6A068	Cell division cycle 5-like protein OS=Mus musculus OX=10090 GN=Cdc5l PE=1 SV=2	Cdc5l	7	5	8	5	802	92.1	8.02	71702	ENSMUSG0	mmu:7170	1180216	864188.5	7549171	4049933	1147038	8502331		3516772	1538197
High	Q8K4R9	Disks large-associated protein 5 OS=Mus musculus OX=10090 GN=Dlgap5 PE=1 SV=2	Dlgap5	6	4	7	4	808	90.1	9.17	218977		mmu:2189	3387067	6574553	11428818	5637472	2348530	6242296	5474255	13649051	2944150
High	P12815	Programmed cell death protein 6 OS=Mus musculus OX=10090 GN=Pdcd6 PE=1 SV=2	Pdcd6	20	2	3	2	191	21.9	5.4	18570	ENSMUSG0	mmu:1857									
High	P42567	Epidermal growth factor receptor substrate 15 OS=Mus musculus OX=10090 GN=Eps15 PE=1 SV=1	Eps15	8	4	7	4	897	98.4	4.6	13858	ENSMUSG0	mmu:1385		262490.5	1412775			719447.1	876690		1118067
High	Q61598	Rab GDP dissociation inhibitor beta OS=Mus musculus OX=10090 GN=Gdi2 PE=1 SV=1	Gdi2	10	4	6	4	445	50.5	6.25	14569	ENSMUSG0	mmu:1456									
High	Q3URU2	Paternally-expressed gene 3 protein OS=Mus musculus OX=10090 GN=Peg3 PE=1 SV=1	Peg3	5	6	11	6	1571	178.8	5.45	18616	ENSMUSG0	mmu:1861	1375219	3985571	2576294	1044186		4051419	951064.7	2902851	1552337
High	Q80XQ2	TBC1 domain family member 5 OS=Mus musculus OX=10090 GN=Tbc1d5 PE=1 SV=2	Tbc1d5	5	3	13	3	815	91.8	6.79	72238	ENSMUSG0	E9PZJ9; mm		345064.7	3097123	1756826	508620.9	3221294	1049885		
High	P62071	Ras-related protein R-Ras2 OS=Mus musculus OX=10090 GN=Rras2 PE=1 SV=1	Rras2	11	2	4	1	204	23.4	6.01	66922	ENSMUSG0	mmu:6692	1566230					2903564			640042.5
High	Q5SUR0	Phosphoribosylformylglycinamidine synthase OS=Mus musculus OX=10090 GN=Pfas PE=1 SV=1	Pfas	2	2	4	2	1337	144.5	5.67	237823	ENSMUSG0	A4FUJ6; mn									
High	Q61187	Tumor susceptibility gene 101 protein OS=Mus musculus OX=10090 GN=Tsg101 PE=1 SV=2	Tsg101	8	3	8	3	391	44.1	6.71	22088	ENSMUSG0	Q61187			2768560		845679.9	2529187		5179119	1296731
High	Q62073	Mitogen-activated protein kinase kinase kinase 7 OS=Mus musculus OX=10090 GN=Map3k7 PE=1 SV=1	Map3k7	6	2	6	2	579	64.2	6.67	26409	ENSMUSG0	mmu:2640	866528.4	2455022	847733.1	996651.1	1387524		851022.1		
High	O88544	COP9 signalosome complex subunit 4 OS=Mus musculus OX=10090 GN=Cops4 PE=1 SV=1	Cops4	6	2	7	2	406	46.3	5.83	26891	ENSMUSG0	mmu:2689			841643.7	1003909		1467198	862492.6	9913829	3080502
High	P62962	Profilin-1 OS=Mus musculus OX=10090 GN=Pfn1 PE=1 SV=2	Pfn1	23	2	2	2	140	14.9	8.28	18643	ENSMUSG0	mmu:1864						969528.9		2068787	1838510
High	Q04447	Creatine kinase B-type OS=Mus musculus OX=10090 GN=Ckb PE=1 SV=1	Ckb	3	2	4	2	381	42.7	5.67	12709	ENSMUSG0	mmu:1270				4392178	978573.7	2470898	1427748		715935.9
High	Q9JHS4	ATP-dependent Clp protease ATP-binding subunit clpX-like, mitochondrial OS=Mus musculus OX=10090 GN=Clpx PE=1 SV=2	Clpx	3	2	6	2	634	69.2	7.71	270166	ENSMUSG0	E9QLZ8; mr	307405.2	496494.9	1391722		798731.9	1755376		3935691	2009644
High	P11087	Collagen alpha-1(I) chain OS=Mus musculus OX=10090 GN=Col1a1 PE=1 SV=4	Col1a1	3	3	7	3	1453	137.9	5.85	12842	ENSMUSG0	mmu:1284									
High	Q9D287	Pre-mRNA-splicing factor SPF27 OS=Mus musculus OX=10090 GN=Bcas2 PE=1 SV=1	Bcas2	19	3	9	3	225	26.1	5.66	68183	ENSMUSG0	mmu:6818		117578.7	394974.2					751301.3	576554.3
High	Q8BSY0	Aspartyl/asparaginyl beta-hydroxylase OS=Mus musculus OX=10090 GN=Asph PE=1 SV=1	Asph	17	9	16	9	741	83	5.08	65973	ENSMUSG0	mmu:6597	2548645	667456.9	162946.5	2414282	2352876	5909428	1439727	5880311	381007.6
High	P46664	Adenylosuccinate synthetase isozyme 2 OS=Mus musculus OX=10090 GN=Adss2 PE=1 SV=2	Adss2	8	2	4	2	456	50	6.38	11566	ENSMUSG0	mmu:1156			1138216	1856511			2986435		636602.6
High	P31324	cAMP-dependent protein kinase type II-beta regulatory subunit OS=Mus musculus OX=10090 GN=Prkar2b PE=1 SV=3	Prkar2b	5	2	3	2	416	46.1	4.98	19088	ENSMUSG0	B1B199; mr	22442288	2891761	4980340	3397884	1100928	5041737		19620606	11015570
High	P83093	Stromal interaction molecule 2 OS=Mus musculus OX=10090 GN=Stim2 PE=1 SV=2	Stim2	4	2	3	2	746	83.9	6.79	116873	ENSMUSG0	mmu:1168									
High	Q80U78	Pumilio homolog 1 OS=Mus musculus OX=10090 GN=Pum1 PE=1 SV=2	Pum1	6	6	15	4	1189	126.5	6.86	80912	ENSMUSG0	mmu:8091	1916368	1143597	9186110		1970119	3052522			834037
High	Q9CPR5	39S ribosomal protein L15, mitochondrial OS=Mus musculus OX=10090 GN=Mrpl15 PE=1 SV=1	Mrpl15	10	2	6	2	295	33.5	10.07	27395	ENSMUSG0	mmu:2739		1959503	3237836			6724731	2380577		
High	Q9R1J0	Sterol-4-alpha-carboxylate 3-dehydrogenase, decarboxylating OS=Mus musculus OX=10090 GN=Nsdhl PE=1 SV=1	Nsdhl	6	2	4	2	362	40.7	7.85	18194	ENSMUSG0	mmu:1819	195101.2							5815179	
High	Q9DCD5	Tight junction-associated protein 1 OS=Mus musculus OX=10090 GN=Tjap1 PE=1 SV=2	Tjap1	17	6	12	6	549	60.5	6.14	74094	ENSMUSG0	I7H459; mr	3260070	2176617	3268897	3125920	739293.8	6194315	3678089	10584429	2848048
High	Q8CIE6	Coatomer subunit alpha OS=Mus musculus OX=10090 GN=Copa PE=1 SV=2	Copa	7	7	11	7	1224	138.3	7.65	12847	ENSMUSG0	E9Q075; mr	2140454	422338.2	1569638	2267389		939802.4	1335353		1787829
High	Q91ZV0	Melanoma inhibitory activity protein 2 OS=Mus musculus OX=10090 GN=Mia2 PE=1 SV=3	Mia2	2	4	6	4	1396	156.4	4.55			Q8CIE3; Q8	345905.3		1234946						
High	P35441	Thrombospondin-1 OS=Mus musculus OX=10090 GN=Thbs1 PE=1 SV=1	Thbs1	5	6	8	6	1170	129.6	4.96			P35441	3576095		419346.6						692033.6
High	B1AZI6	THO complex subunit 2 OS=Mus musculus OX=10090 GN=Thoc2 PE=1 SV=1	Thoc2	2	4	12	4	1594	182.7	8.44	331401	ENSMUSG0	B1AZI6; mm	290402.2	252711.9	1101173						
High	Q9JLQ2	ARF GTPase-activating protein GIT2 OS=Mus musculus OX=10090 GN=Git2 PE=1 SV=2	Git2	2	2	3	2	708	78.7	7.72	26431	ENSMUSG0	E9QPU7; m									
High	Q8BIJ7	RUN and FYVE domain-containing protein 1 OS=Mus musculus OX=10090 GN=Rufy1 PE=1 SV=1	Rufy1	4	2	5	2	712	80.3	5.68	216724	ENSMUSG0	mmu:2167		482079.5	996174						
High	Q91ZU6-5	Isoform 5 of Dystonin OS=Mus musculus OX=10090 GN=Dst	Dst	1	2	19	1	2639	304.6	6.54			Q91ZU6-5	1104472	2199578	7080923	4986022	5391310	5923078	3572618	3932200	1071301
High	Q05A36	RNA-binding E3 ubiquitin-protein ligase MEX3C OS=Mus musculus OX=10090 GN=Mex3c PE=2 SV=2	Mex3c	2	2	3	2	652	68.5	4.98	240396	ENSMUSG0	Q05A36; Q2									
High	Q5SSI6	U3 small nucleolar RNA-associated protein 18 homolog OS=Mus musculus OX=10090 GN=Utp18 PE=1 SV=1	Utp18	6	4	4	4	552	61.2	8.78	217109	ENSMUSG0	mmu:2171			1008864						
High	Q9QWY8	Arf-GAP with SH3 domain, ANK repeat and PH domain-containing protein 1 OS=Mus musculus OX=10090 GN=Asap1 PE=1 SV=2	Asap1	2	2	6	2	1147	127.3	7.64	13196	ENSMUSG0	mmu:1319			2368598	2464772	1081309	2426139	824823.8	2454692	348059.8
High	P97465	Docking protein 1 OS=Mus musculus OX=10090 GN=Dok1 PE=1 SV=2	Dok1	4	2	4	2	482	52.4	6.57	13448	ENSMUSG0	mmu:1344	2410767	566405.4	2214146	1334486	586410.3	3438541		2454983	553822.6
High	P26638	Serine--tRNA ligase, cytoplasmic OS=Mus musculus OX=10090 GN=Sars1 PE=1 SV=3	Sars1	6	2	9	2	512	58.4	6.3	20226	ENSMUSG0	A2AFR8; mr		574935.4	768367.1		577946.8				
High	Q61941	NAD(P) transhydrogenase, mitochondrial OS=Mus musculus OX=10090 GN=Nnt PE=1 SV=2	Nnt	2	2	5	2	1086	113.8	7.64			Q61941; Q9				4432811			4512974	1772112	
High	Q91WK0	Leucine-rich repeat flightless-interacting protein 2 OS=Mus musculus OX=10090 GN=Lrrfip2 PE=1 SV=1	Lrrfip2	6	2	3	1	415	47.1	5.68	71268		mmu:7126		583946.5	857624.4					1462716	
High	Q9D6R2	Isocitrate dehydrogenase [NAD] subunit alpha, mitochondrial OS=Mus musculus OX=10090 GN=Idh3a PE=1 SV=1	Idh3a	8	3	4	3	366	39.6	6.73	67834	ENSMUSG0	mmu:6783									703779
High	Q05D44	Eukaryotic translation initiation factor 5B OS=Mus musculus OX=10090 GN=Eif5b PE=1 SV=2	Eif5b	2	2	8	2	1216	137.5	5.59	226982	ENSMUSG0	mmu:2269	1375425	1503105	5070222	1672279	1553133	2472321	2576807	3601885	973058.6
High	O54833	Casein kinase II subunit alpha' OS=Mus musculus OX=10090 GN=Csnk2a2 PE=1 SV=1	Csnk2a2	8	3	6	2	350	41.2	8.56	13000	ENSMUSG0	mmu:1300	1547348		2302879						
High	Q8K2T8	RNA polymerase II-associated factor 1 homolog OS=Mus musculus OX=10090 GN=Paf1 PE=1 SV=1	Paf1	5	2	6	2	535	60.5	4.65	54624	ENSMUSG0	mmu:5462		1324999	2095970		823047.9	1335070			323403.7
High	Q99PT1	Rho GDP-dissociation inhibitor 1 OS=Mus musculus OX=10090 GN=Arhgdia PE=1 SV=3	Arhgdia	24	2	5	2	204	23.4	5.2	192662	ENSMUSG0	mmu:1926		1144716						4389110	2027875
High	P70349	Adenosine 5'-monophosphoramidase HINT1 OS=Mus musculus OX=10090 GN=Hint1 PE=1 SV=3	Hint1	17	2	8	2	126	13.8	6.87	15254	ENSMUSG0	mmu:1525		3669390	3619995	816178.2	3273316			4472908	4028680
High	Q99L47	Hsc70-interacting protein OS=Mus musculus OX=10090 GN=St13 PE=1 SV=1	St13	6	2	2	2	371	41.6	5.26	70356	ENSMUSG0	mmu:7035									
High	Q8BL66	Early endosome antigen 1 OS=Mus musculus OX=10090 GN=Eea1 PE=1 SV=2	Eea1	4	5	11	5	1411	160.8	5.77	216238	ENSMUSG0	mmu:2162			1648194						695633.4
High	P97461	40S ribosomal protein S5 OS=Mus musculus OX=10090 GN=Rps5 PE=1 SV=4	Rps5	30	6	7	6	204	22.9	9.72	20103	ENSMUSG0	mmu:2010	768052.8	1457174	2789142	1822213	626812	980038.8	1150815	5386699	5502965
High	Q99JB8	Protein kinase C and casein kinase II substrate protein 3 OS=Mus musculus OX=10090 GN=Pacsin3 PE=1 SV=1	Pacsin3	5	2	2	2	424	48.6	6.1	80708	ENSMUSG0	mmu:8070									
High	P34022	Ran-specific GTPase-activating protein OS=Mus musculus OX=10090 GN=Ranbp1 PE=1 SV=2	Ranbp1	10	3	8	3	203	23.6	5.22	19385	ENSMUSG0	mmu:1938	2088755	3536688	10010924	3255693		8384265	2535056	11292648	2767584
High	A6X8Z5	Rho GTPase-activating protein 31 OS=Mus musculus OX=10090 GN=Arhgap31 PE=1 SV=1	Arhgap31	3	3	5	3	1425	155.2	5.77	12549	ENSMUSG0	A6X8Z5; mr		1513493	1619571						
High	O08756	3-hydroxyacyl-CoA dehydrogenase type-2 OS=Mus musculus OX=10090 GN=Hsd17b10 PE=1 SV=4	Hsd17b10	20	5	12	5	261	27.4	8.41			O08756								1083022	1250745
High	Q62417-7	Isoform 7 of Sorbin and SH3 domain-containing protein 1 OS=Mus musculus OX=10090 GN=Sorbs1	Sorbs1	6	4	5	4	836	92	7.85			Q62417-7		365008.9	560339.8	5089264	658805.6		6567992	1198993	453837.1
High	Q9ERU9	E3 SUMO-protein ligase RanBP2 OS=Mus musculus OX=10090 GN=Ranbp2 PE=1 SV=2	Ranbp2	2	4	6	4	3053	340.9	6.18	19386	ENSMUSG0	E9QM01; m									
High	Q8R4R6	Nucleoporin NUP35 OS=Mus musculus OX=10090 GN=Nup35 PE=1 SV=2	Nup35	15	3	7	3	325	34.8	9.25	69482	ENSMUSG0	A2ATJ3; mn	566469.1	1198005	4102032		749603.4	1288376	1016605	1535286	685138.4
High	A2APB8	Targeting protein for Xklp2 OS=Mus musculus OX=10090 GN=Tpx2 PE=1 SV=1	Tpx2	6	5	12	5	745	85.8	9.38	72119	ENSMUSG0	A2APB8; mr	3985132	3359583	9197502	2218912	2442593	5902982	2552895	29429140	28681063
High	P63037	DnaJ homolog subfamily A member 1 OS=Mus musculus OX=10090 GN=Dnaja1 PE=1 SV=1	Dnaja1	10	3	6	3	397	44.8	7.08	15502	ENSMUSG0	mmu:1550	550569.8	795494.7	2316702			1612789		4603677	1758620
High	Q9CZ04	COP9 signalosome complex subunit 7a OS=Mus musculus OX=10090 GN=Cops7a PE=1 SV=2	Cops7a	13	3	3	3	275	30.2	7.87	26894	ENSMUSG0	mmu:2689									1231690
High	Q60862	Origin recognition complex subunit 2 OS=Mus musculus OX=10090 GN=Orc2 PE=1 SV=1	Orc2	3	2	3	2	576	65.9	6.81	18393	ENSMUSG0	mmu:1839	331159.6	931169.3	2400725		477383				
High	Q9ESV0	ATP-dependent RNA helicase DDX24 OS=Mus musculus OX=10090 GN=Ddx24 PE=1 SV=2	Ddx24	4	3	7	3	857	96.4	9.36	27225	ENSMUSG0	mmu:2722	2023777	1279412	4612105						
High	O70251	Elongation factor 1-beta OS=Mus musculus OX=10090 GN=Eef1b PE=1 SV=5	Eef1b	10	2	5	2	225	24.7	4.69	55949	ENSMUSG0	mmu:5594	293490.8			1453214	564625.3			2430675	13295311
High	D3YXK1	Sterile alpha motif domain-containing protein 1 OS=Mus musculus OX=10090 GN=Samd1 PE=1 SV=1	Samd1	6	2	2	2	519	54.5	7.47	666704	ENSMUSG0	D3YXK1; mr		732843.5							
High	P12032	Metalloproteinase inhibitor 1 OS=Mus musculus OX=10090 GN=Timp1 PE=1 SV=2	Timp1	9	2	3	2	205	22.6	8.82	21857	ENSMUSG0	mmu:2185	3572450	504539.4	2219703	1240176	815146.3	4622938		10680069	2126226
High	P35235-1	Isoform 2 of Tyrosine-protein phosphatase non-receptor type 11 OS=Mus musculus OX=10090 GN=Ptpn11	Ptpn11	6	3	5	3	597	68.4	7.3			P35235-1									
High	Q9QYL7	Activator of basal transcription 1 OS=Mus musculus OX=10090 GN=Abt1 PE=2 SV=1	Abt1	9	2	6	2	269	30.6	10.01	30946	ENSMUSG0	mmu:3094	660268.7	1460051	3053914	1925783	784249.1	2211681	1491600	1440214	1940596
High	Q99P72	Reticulon-4 OS=Mus musculus OX=10090 GN=Rtn4 PE=1 SV=2	Rtn4	6	5	17	5	1162	126.5	4.54	68585	ENSMUSG0	mmu:6858	929136.9	1875983	1282735	4928146		1676696	6600208	4248010	697866.3
High	Q9CPQ8	ATP synthase subunit g, mitochondrial OS=Mus musculus OX=10090 GN=Atp5mg PE=1 SV=1	Atp5mg	43	3	6	3	103	11.4	9.74	27425	ENSMUSG0	mmu:2742				1196770			1573123	7848329	3586880
High	Q9JJ80	Ribosome production factor 2 homolog OS=Mus musculus OX=10090 GN=Rpf2 PE=2 SV=2	Rpf2	8	2	6	2	306	35.3	10.04	67239	ENSMUSG0	mmu:6723	873599.4	204790.5	890327.6						
High	Q99MI1	ELKS/Rab6-interacting/CAST family member 1 OS=Mus musculus OX=10090 GN=Erc1 PE=1 SV=1	Erc1	2	2	2	2	1120	128.3	5.87	111173	ENSMUSG0	mmu:1111	510446.1	380693.6	1220083		394582.2			1117734	874461.3
High	Q5NBX1	Protein cordon-bleu OS=Mus musculus OX=10090 GN=Cobl PE=1 SV=1	Cobl	2	3	6	3	1337	143.8	7.77	12808	ENSMUSG0	mmu:1280				3152059				1144646	1181190
High	Q08943	FACT complex subunit SSRP1 OS=Mus musculus OX=10090 GN=Ssrp1 PE=1 SV=2	Ssrp1	5	3	10	3	708	80.8	6.76	20833	ENSMUSG0	mmu:2083		235255.3	2594017		244569.3	1715693	843469		487170.9
High	O35071	Kinesin-like protein KIF1C OS=Mus musculus OX=10090 GN=Kif1c PE=1 SV=2	Kif1c	2	2	7	2	1100	122.4	7.03	16562	ENSMUSG0	O35071; Q5	772574.2	393320.8	2348460	2373017		1503972	1427876	4568231	1495584
High	Q3UB74	Transforming growth factor beta regulator 1 OS=Mus musculus OX=10090 GN=Tbrg1 PE=1 SV=2	Tbrg1	5	2	5	2	406	44.9	7.59	21376	ENSMUSG0	mmu:2137	242237.6	940467.6	747158.2			722325.8		1701311	572412.8
High	Q3TDQ1	Dolichyl-diphosphooligosaccharide--protein glycosyltransferase subunit STT3B OS=Mus musculus OX=10090 GN=Stt3b PE=1 SV=2	Stt3b	2	2	5	2	823	93.2	8.95	68292		mmu:6829	1854448	1002354	1567669	2968153	645940.1	2441526	2237043	1706646	
High	P61202	COP9 signalosome complex subunit 2 OS=Mus musculus OX=10090 GN=Cops2 PE=1 SV=1	Cops2	3	2	3	2	443	51.6	5.53	12848	ENSMUSG0	mmu:1284						1343989		3774463	1512882
High	Q6NSQ7	Protein LTV1 homolog OS=Mus musculus OX=10090 GN=Ltv1 PE=1 SV=2	Ltv1	4	2	6	2	470	54	4.92	353258	ENSMUSG0	mmu:3532	410546.5	1176182	2274105	180900.5	892549.6	1309422			473416.9
High	Q64511	DNA topoisomerase 2-beta OS=Mus musculus OX=10090 GN=Top2b PE=1 SV=2	Top2b	1	2	4	1	1612	181.8	8.29	21974	ENSMUSG0	mmu:2197									
High	Q8R3C0	Mini-chromosome maintenance complex-binding protein OS=Mus musculus OX=10090 GN=Mcmbp PE=1 SV=1	Mcmbp	3	2	9	2	642	72.8	5.66	210711	ENSMUSG0	D3Z3G1; m	4630275	8476766	9103730	5776513	4507008	12982653	7440705	15186871	2694221
High	Q9Z2I8	Succinate--CoA ligase [GDP-forming] subunit beta, mitochondrial OS=Mus musculus OX=10090 GN=Suclg2 PE=1 SV=3	Suclg2	9	3	6	3	433	46.8	7.02	20917	ENSMUSG0	mmu:2091	1896471	9499805	6070667	7413800	6577306	10596720	7926061	12310140	2272061
High	P57724	Poly(rC)-binding protein 4 OS=Mus musculus OX=10090 GN=Pcbp4 PE=1 SV=1	Pcbp4	6	3	23	1	403	41.4	8.34	59092		mmu:5909	1494034	724051	1597797		455503.8	2259703		3000409	932800.9
High	P21278	Guanine nucleotide-binding protein subunit alpha-11 OS=Mus musculus OX=10090 GN=Gna11 PE=1 SV=1	Gna11	7	2	3	2	359	42	5.97	14672	ENSMUSG0	mmu:1467	2158267	350615.2	1706046	1553056		1323931			
High	Q3UHC7	Disabled homolog 2-interacting protein OS=Mus musculus OX=10090 GN=Dab2ip PE=1 SV=1	Dab2ip	2	3	5	3	1189	131.6	8.72	69601	ENSMUSG0	A2AUW9; A			1071577	3657125	1645383	1663486	959968.9	1725649	789820.2
High	Q6PGF7	Exocyst complex component 8 OS=Mus musculus OX=10090 GN=Exoc8 PE=1 SV=1	Exoc8	3	2	5	2	716	81	5.4	102058	ENSMUSG0	mmu:1020	510318.4	2734269	3305363	4863204	3448006	4109753	3683901	10048486	1008472
High	Q9D7B7	Probable glutathione peroxidase 8 OS=Mus musculus OX=10090 GN=Gpx8 PE=1 SV=1	Gpx8	9	2	6	2	209	24.1	9.36	69590	ENSMUSG0	mmu:6959									
High	Q9QYH6	Melanoma-associated antigen D1 OS=Mus musculus OX=10090 GN=Maged1 PE=1 SV=1	Maged1	3	2	3	2	775	85.6	7.5	94275	ENSMUSG0	mmu:9427			1850914	892378.3		836348.1			
High	Q60710	Deoxynucleoside triphosphate triphosphohydrolase SAMHD1 OS=Mus musculus OX=10090 GN=Samhd1 PE=1 SV=3	Samhd1	5	4	12	4	658	75.8	7.93	56045	ENSMUSG0	E9Q0K6; F8	6494996	1126510	4084011			3413061		12924233	1584035
High	Q9ERK4	Exportin-2 OS=Mus musculus OX=10090 GN=Cse1l PE=1 SV=1	Cse1l	6	6	9	6	971	110.4	5.77	110750	ENSMUSG0	mmu:1107	999902.4		2196202	824909.5	461714.5	3034150		4954759	2016340
High	P21619	Lamin-B2 OS=Mus musculus OX=10090 GN=Lmnb2 PE=1 SV=2	Lmnb2	3	3	25	1	596	67.3	5.5	16907	ENSMUSG0	mmu:1690									
High	Q3TJD7-2	Isoform 2 of PDZ and LIM domain protein 7 OS=Mus musculus OX=10090 GN=Pdlim7	Pdlim7	38	6	12	1	188	21.1	9.5			Q3TJD7-2									
High	Q9QXB9	Developmentally-regulated GTP-binding protein 2 OS=Mus musculus OX=10090 GN=Drg2 PE=1 SV=1	Drg2	5	2	5	1	364	40.7	8.88	13495	ENSMUSG0	mmu:1349	641588.8	220106.5	847818	1276523		894359.4	823053.7	4081393	
High	O88746	Target of Myb1 membrane trafficking protein OS=Mus musculus OX=10090 GN=Tom1 PE=1 SV=1	Tom1	6	3	6	3	492	54.3	4.94	21968	ENSMUSG0	mmu:2196		663657.1	912662.7	1865721			3390599		556671.2
High	O70503	Very-long-chain 3-oxoacyl-CoA reductase OS=Mus musculus OX=10090 GN=Hsd17b12 PE=1 SV=1	Hsd17b12	11	4	7	4	312	34.7	9.52	56348	ENSMUSG0	mmu:5634	2088471	560999.9	1061976		792148.1	4037647		5689190	1621594
High	Q6PFD9	Nuclear pore complex protein Nup98-Nup96 OS=Mus musculus OX=10090 GN=Nup98 PE=1 SV=2	Nup98	2	2	4	2	1816	197.1	6.18	269966		mmu:2699	522940.7		1620205		673221				307970.1
High	Q8BW10	RNA-binding protein NOB1 OS=Mus musculus OX=10090 GN=Nob1 PE=1 SV=1	Nob1	12	3	7	3	403	45.4	7.31	67619	ENSMUSG0	mmu:6761	1061823	246129.7	1379421			972979.4			

High	Q6NXI6	Regulation of nuclear pre-mRNA domain-containing protein 2 OS=Mus musculus OX=10090 GN=Rprd2 PE=1 SV=1	Rprd2	2	2	2	2	1469	156.5	7.59	75137	ENSMUSG0	mmu:7513	224207.1	718388.1	353615			724145.9		1579373	
High	Q60596	DNA repair protein XRCC1 OS=Mus musculus OX=10090 GN=Xrcc1 PE=1 SV=2	Xrcc1	4	2	6	2	631	68.9	6.33	22594	ENSMUSG0	mmu:2259									
High	Q03145	Ephrin type-A receptor 2 OS=Mus musculus OX=10090 GN=Epha2 PE=1 SV=3	Epha2	7	6	6	6	977	108.8	6.23	13836	ENSMUSG0	mmu:1383	513374.7								
High	Q8CB77	Elongin-A OS=Mus musculus OX=10090 GN=Eloa PE=1 SV=3	Eloa	4	2	2	2	773	87.1	9.61	27224	ENSMUSG0	mmu:2722			862448.8		407494.5				
High	P53564	Homeobox protein cut-like 1 OS=Mus musculus OX=10090 GN=Cux1 PE=1 SV=3	Cux1	3	3	5	3	1515	165.5	6.32	13047		mmu:1304			1434290						
High	Q9R0U0	Serine/arginine-rich splicing factor 10 OS=Mus musculus OX=10090 GN=Srsf10 PE=1 SV=2	Srsf10	6	2	3	2	262	31.3	11.27	14105	ENSMUSG0	B1AV44; B1									
High	Q8R1S4	Protein MTSS 1 OS=Mus musculus OX=10090 GN=Mtss1 PE=1 SV=1	Mtss1	3	2	8	2	759	82.4	6.8	211401		mmu:2114			1412929						
High	Q0VBL3	RNA-binding protein 15 OS=Mus musculus OX=10090 GN=Rbm15 PE=1 SV=1	Rbm15	3	2	3	2	962	105.7	10.08	229700	ENSMUSG0	A0PJG5; mr									
High	Q9JMD0	BUB3-interacting and GLEBS motif-containing protein ZNF207 OS=Mus musculus OX=10090 GN=Znf207 PE=1 SV=1	Znf207	4	3	11	3	495	52.8	9.1	22680	ENSMUSG0	E9PW12; m		801637.3	846115.6		1088916		1055073		
High	Q60973	Histone-binding protein RBBP7 OS=Mus musculus OX=10090 GN=Rbbp7 PE=1 SV=1	Rbbp7	8	3	6	2	425	47.8	5.05	245688	ENSMUSG0	A2AFJ0; Q3	963417.9	258911.4			376695.4				1313383
High	Q91X43	SH3 domain-containing protein 19 OS=Mus musculus OX=10090 GN=Sh3d19 PE=1 SV=2	Sh3d19	5	3	5	3	789	86	8.53	27059	ENSMUSG0	mmu:2705		801463.1	860663.1		673048.8				
High	Q8CFI7	DNA-directed RNA polymerase II subunit RPB2 OS=Mus musculus OX=10090 GN=Polr2b PE=1 SV=2	Polr2b	3	4	7	4	1174	133.8	6.87	231329	ENSMUSG0	mmu:2313									
High	Q61210	Rho guanine nucleotide exchange factor 1 OS=Mus musculus OX=10090 GN=Arhgef1 PE=1 SV=2	Arhgef1	5	3	6	3	920	102.7	5.6	16801	ENSMUSG0	mmu:1680									
High	O88712	C-terminal-binding protein 1 OS=Mus musculus OX=10090 GN=Ctbp1 PE=1 SV=2	Ctbp1	8	3	3	3	441	47.7	6.77	13016	ENSMUSG0	mmu:1301	581628.6		728266.2	2204296	350296.2	4700697		5429813	2395939
High	F6ZDS4	Nucleoprotein TPR OS=Mus musculus OX=10090 GN=Tpr PE=1 SV=1	Tpr	3	6	10	6	2431	273.8	5.03	108989	ENSMUSG0	F6ZDS4; mr			181816.5	269383.7			277285.1	467659.3	302999
High	Q8CGZ0	Calcium homeostasis endoplasmic reticulum protein OS=Mus musculus OX=10090 GN=Cherp PE=1 SV=1	Cherp	4	3	5	3	936	106.1	9.14	27967		mmu:2796		732349.7	1060208		829141.1				
High	P17742	Peptidyl-prolyl cis-trans isomerase A OS=Mus musculus OX=10090 GN=Ppia PE=1 SV=2	Ppia	19	2	3	2	164	18	7.9	268373	ENSMUSG0	mmu:2683								2914815	1067001
High	Q01405	Protein transport protein Sec23A OS=Mus musculus OX=10090 GN=Sec23a PE=1 SV=2	Sec23a	3	2	2	2	765	86.1	7.08	20334	ENSMUSG0	mmu:2033				2038834		582662.9	486008.2		216716.8
High	Q60931	Voltage-dependent anion-selective channel protein 3 OS=Mus musculus OX=10090 GN=Vdac3 PE=1 SV=1	Vdac3	7	2	3	1	283	30.7	8.79	22335	ENSMUSG0	mmu:2233									
High	Q8VH51	RNA-binding protein 39 OS=Mus musculus OX=10090 GN=Rbm39 PE=1 SV=2	Rbm39	3	2	8	2	530	59.4	10.1	170791	ENSMUSG0	mmu:1707									
High	Q8BLY2	Threonine--tRNA ligase 2, cytoplasmic OS=Mus musculus OX=10090 GN=Tars3 PE=1 SV=1	Tars3	3	2	3	1	790	91.3	7.53	272396	ENSMUSG0	mmu:2723									
High	Q9D2G2	Dihydrolipoyllysine-residue succinyltransferase component of 2-oxoglutarate dehydrogenase complex, mitochondrial OS=Mus musculus O	Dlst	6	3	3	3	454	49	8.95	78920	ENSMUSG0	mmu:7892				1259345				2547250	4295360
High	Q9D0N7	Chromatin assembly factor 1 subunit B OS=Mus musculus OX=10090 GN=Chaf1b PE=1 SV=1	Chaf1b	4	2	4	2	572	63.1	6.55	110749	ENSMUSG0	mmu:1107	707024.9	1261113	1139347		572665.1			2603126	
High	P49718	DNA replication licensing factor MCM5 OS=Mus musculus OX=10090 GN=Mcm5 PE=1 SV=2	Mcm5	4	3	4	3	734	82.4	8.29	17218	ENSMUSG0	mmu:1721	847606.1								
High	Q8BTW9	Serine/threonine-protein kinase PAK 4 OS=Mus musculus OX=10090 GN=Pak4 PE=1 SV=1	Pak4	6	2	3	2	593	64.6	9.85	70584	ENSMUSG0	mmu:7058									
High	Q6VN19	Ran-binding protein 10 OS=Mus musculus OX=10090 GN=Ranbp10 PE=1 SV=2	Ranbp10	4	2	3	2	620	67.1	6.58	74334	ENSMUSG0	mmu:7433									
High	Q8BY87	Ubiquitin carboxyl-terminal hydrolase 47 OS=Mus musculus OX=10090 GN=Usp47 PE=1 SV=2	Usp47	1	2	8	2	1376	157.4	5.11	74996	ENSMUSG0	mmu:7499	985154.4	999395.5	3080724		934078.8	2446903		5240637	1991048
High	P28660	Nck-associated protein 1 OS=Mus musculus OX=10090 GN=Nckap1 PE=1 SV=2	Nckap1	2	2	2	2	1128	128.7	6.62	50884	ENSMUSG0	mmu:5088									
High	Q8CCP0	Ribosome quality control complex subunit NEMF OS=Mus musculus OX=10090 GN=Nemf PE=1 SV=2	Nemf	2	2	6	2	1064	121.1	6.8	66244	ENSMUSG0	mmu:6624	1225200	1165744	9985767	5982824	1920668	4540313		9443376	1240966
High	P22682	E3 ubiquitin-protein ligase CBL OS=Mus musculus OX=10090 GN=Cbl PE=1 SV=3	Cbl	3	2	7	2	913	100.5	6.67	12402	ENSMUSG0	P22682; Q3		584887.5	2896690	1320068	1081357	2088919	2410485		
High	P51150	Ras-related protein Rab-7a OS=Mus musculus OX=10090 GN=Rab7a PE=1 SV=2	Rab7a	12	2	3	2	207	23.5	6.7	19349	ENSMUSG0	mmu:1934	2116713						1232180		
High	Q9JIF7	Coatomer subunit beta OS=Mus musculus OX=10090 GN=Copb1 PE=1 SV=1	Copb1	5	4	5	4	953	107	6	70349	ENSMUSG0	mmu:7034	578672.1		1155327						
High	A2A7S8-2	Isoform 2 of Uncharacterized protein KIAA1522 OS=Mus musculus OX=10090 GN=Kiaa1522	Nhsl3	4	4	11	4	1070	110.6	10.07			A2A7S8-2			251601.5		136901.1	277884.6			
High	Q8BL97	Serine/arginine-rich splicing factor 7 OS=Mus musculus OX=10090 GN=Srsf7 PE=1 SV=1	Srsf7	10	2	4	2	267	30.8	11.9	225027	ENSMUSG0	mmu:2250									
High	Q924A2	Protein capicua homolog OS=Mus musculus OX=10090 GN=Cic PE=1 SV=2	Cic	2	2	3	2	2510	258	8.02	71722	ENSMUSG0	mmu:7172		1577384							
High	Q9QZS0	Collagen alpha-3(IV) chain OS=Mus musculus OX=10090 GN=Col4a3 PE=1 SV=2	Col4a3	1	2	5	2	1669	161.6	9.48	12828	ENSMUSG0	mmu:1282	644355.1		1200582			2330989			1678341
High	Q99NB8	Ubiquilin-4 OS=Mus musculus OX=10090 GN=Ubqln4 PE=1 SV=1	Ubqln4	5	2	3	2	596	63.5	5.03	94232	ENSMUSG0	mmu:9423		1366678	2427756		348131.9		1097050	2311480	
High	P61079	Ubiquitin-conjugating enzyme E2 D3 OS=Mus musculus OX=10090 GN=Ube2d3 PE=1 SV=1	Ube2d3	23	2	4	2	147	16.7	7.8	66105	ENSMUSG0	mmu:6610								1050538	872724.5
High	P51174	Long-chain specific acyl-CoA dehydrogenase, mitochondrial OS=Mus musculus OX=10090 GN=Acadl PE=1 SV=2	Acadl	4	2	4	2	430	47.9	8.31	11363		B2KGC6; m	2200766	1958577	1360475	1417585	10065233	1479722		2588828	1371618
High	Q3TIR3	Synembryn-A OS=Mus musculus OX=10090 GN=Ric8a PE=1 SV=2	Ric8a	4	2	2	2	530	59.8	5.68	101489	ENSMUSG0	mmu:1014									
High	Q63918	Caveolae-associated protein 2 OS=Mus musculus OX=10090 GN=Cavin2 PE=1 SV=3	Cavin2	5	2	4	2	418	46.7	5.21	20324	ENSMUSG0	mmu:2032		806419.2	2078072	2043926		2380176		3563964	1130916
High	Q9CQF3	Cleavage and polyadenylation specificity factor subunit 5 OS=Mus musculus OX=10090 GN=Nudt21 PE=1 SV=1	Nudt21	23	4	14	4	227	26.2	8.82	68219	ENSMUSG0	mmu:6821	906920.4	2658997	4808934	2644776	1395768	3403361	2782096	7429444	2111014
High	Q71FD7	Filamin-binding LIM protein 1 OS=Mus musculus OX=10090 GN=Fblim1 PE=1 SV=2	Fblim1	6	2	5	2	375	41	6.51	74202	ENSMUSG0	mmu:7420	341062.7	1045911	1033918	810257.6	735836.9	923048.4	1132531	2204967	673782
High	Q8VDN2	Sodium/potassium-transporting ATPase subunit alpha-1 OS=Mus musculus OX=10090 GN=Atp1a1 PE=1 SV=1	Atp1a1	5	5	11	5	1023	112.9	5.45	11928	ENSMUSG0	mmu:1192	1042523	1698733	2980071	1714719	2038979	825158.2	6544949	2262546	2016716
High	P70677	Caspase-3 OS=Mus musculus OX=10090 GN=Casp3 PE=1 SV=1	Casp3	10	3	4	3	277	31.5	6.92	12367	ENSMUSG0	mmu:1236	593169.4	1776053	1837892	1148678		1990485	1338336	1398908	1101519
High	Q6P5D8	Structural maintenance of chromosomes flexible hinge domain-containing protein 1 OS=Mus musculus OX=10090 GN=Smchd1 PE=1 SV=2	Smchd1	1	2	3	2	2007	225.5	7.24	74355	ENSMUSG0	mmu:7435									
High	Q9Z1W9	STE20/SPS1-related proline-alanine-rich protein kinase OS=Mus musculus OX=10090 GN=Stk39 PE=1 SV=1	Stk39	2	2	6	2	556	60.3	6.29	53416	ENSMUSG0	mmu:5341	748503.6	981263.6	2025563	1667017	1192190	1667526	1286021	1759339	701085.3
High	Q99JX4	Eukaryotic translation initiation factor 3 subunit M OS=Mus musculus OX=10090 GN=Eif3m PE=1 SV=1	Eif3m	12	4	7	4	374	42.5	5.74	98221	ENSMUSG0	mmu:9822	238612.8	114494.9	1298462		467840.9	619808.9			
High	Q3UHX0	Nucleolar protein 8 OS=Mus musculus OX=10090 GN=Nol8 PE=1 SV=2	Nol8	2	2	2	2	1147	128.6	6.55	70930	ENSMUSG0	mmu:7093		475071.8	987569.6		459053.3		1116662		
High	Q6NZC7	SEC23-interacting protein OS=Mus musculus OX=10090 GN=Sec23ip PE=1 SV=2	Sec23ip	4	3	3	3	998	110.7	5.94			Q6NZC7; Q8									
High	P70336	Rho-associated protein kinase 2 OS=Mus musculus OX=10090 GN=Rock2 PE=1 SV=1	Rock2	3	4	12	2	1388	160.5	5.99	19878		A5XDA7; m	1104052	1455922	4019175	2814701	1523097	1586140	971960.4	13827181	717563.8
High	O89079	Coatomer subunit epsilon OS=Mus musculus OX=10090 GN=Cope PE=1 SV=3	Cope	10	2	2	2	308	34.5	5.06	59042	ENSMUSG0	mmu:5904									
High	Q9DBG7	Signal recognition particle receptor subunit alpha OS=Mus musculus OX=10090 GN=Srpra PE=1 SV=1	Srpra	6	3	5	3	636	69.6	8.95	67398	ENSMUSG0	mmu:6739	3824179	12068146	18991648	12841345	12072287	10407895	16808551	15341346	2721825
High	P70288	Histone deacetylase 2 OS=Mus musculus OX=10090 GN=Hdac2 PE=1 SV=1	Hdac2	10	4	12	1	488	55.3	5.91	15182		B2RRP3; m	537465.4		521819.9		296668.5				
High	Q9Z207	Protein diaphanous homolog 3 OS=Mus musculus OX=10090 GN=Diaph3 PE=1 SV=1	Diaph3	2	2	5	2	1171	133.6	7.61	56419	ENSMUSG0	mmu:5641		753000.9	1456027						
High	Q61081	Hsp90 co-chaperone Cdc37 OS=Mus musculus OX=10090 GN=Cdc37 PE=1 SV=1	Cdc37	8	2	3	2	379	44.6	5.34	12539	ENSMUSG0	mmu:1253									
High	Q99KQ4	Nicotinamide phosphoribosyltransferase OS=Mus musculus OX=10090 GN=Nampt PE=1 SV=1	Nampt	6	3	22	3	491	55.4	7.15	59027	ENSMUSG0	mmu:5902	2675497	2211329	4344874	2048944	600790.9	4756164	1373337	6585491	2003151
High	Q9DBG5	Perilipin-3 OS=Mus musculus OX=10090 GN=Plin3 PE=1 SV=1	Plin3	7	3	13	3	437	47.2	5.62	66905	ENSMUSG0	mmu:6690	402394.8	1537995	3526391	6022760	1968148	4067578	5474253	4762686	
High	Q9DB90	Nonsense-mediated mRNA decay factor SMG9 OS=Mus musculus OX=10090 GN=Smg9 PE=1 SV=1	Smg9	8	3	7	3	520	57.6	7.01	71997	ENSMUSG0	mmu:7199		954520.9		5616262	1869260	3877688			
High	Q3U1G5	Interferon-stimulated 20 kDa exonuclease-like 2 OS=Mus musculus OX=10090 GN=Isg20l2 PE=1 SV=2	Isg20l2	8	2	4	2	368	41	10.01	229504		mmu:2295	220695.3	422063.3	340291.1						
High	Q3TC93	HCLS1-binding protein 3 OS=Mus musculus OX=10090 GN=Hs1bp3 PE=1 SV=2	Hs1bp3	6	2	3	2	395	43.7	4.98	58240	ENSMUSG0	E9QLQ4; m		520734.8	843421.9		510985.4		874563.1		
High	A2BH40	AT-rich interactive domain-containing protein 1A OS=Mus musculus OX=10090 GN=Arid1a PE=1 SV=1	Arid1a	4	5	12	5	2283	241.9	6.68	93760	ENSMUSG0	A2BH40; m		1867882	3375663						
High	Q9QYI5	DnaJ homolog subfamily B member 2 OS=Mus musculus OX=10090 GN=Dnajb2 PE=1 SV=3	Dnajb2	7	2	3	2	324	35.6	5.91	56812	ENSMUSG0	mmu:5681									
High	Q5SFM8	RNA-binding protein 27 OS=Mus musculus OX=10090 GN=Rbm27 PE=1 SV=3	Rbm27	3	3	7	3	1060	118.5	9.19	225432	ENSMUSG0	E9QM85; m	4501227	6390555	9822282	2145818		2096815	1309944		983279.9
High	Q9R0E2	Procollagen-lysine,2-oxoglutarate 5-dioxygenase 1 OS=Mus musculus OX=10090 GN=Plod1 PE=1 SV=1	Plod1	5	4	14	4	728	83.5	6.54	18822	ENSMUSG0	mmu:1882	2027605		1628574		241882.1	2142417	522848.6	2111939	735293.9
High	Q62280	Protein SSXT OS=Mus musculus OX=10090 GN=Ss18 PE=1 SV=2	Ss18	3	2	7	2	418	45.8	6.46	268996	ENSMUSG0	mmu:2689		3829406	4885117	1007946	3997714				
High	Q8VC70	RNA-binding motif, single-stranded-interacting protein 2 OS=Mus musculus OX=10090 GN=Rbms2 PE=1 SV=1	Rbms2	13	4	7	4	383	40.7	9.07	56516	ENSMUSG0	mmu:5651		1221189	1635806	1170591	924251.1		1512043		
High	Q9CRB9	MICOS complex subunit Mic19 OS=Mus musculus OX=10090 GN=Chchd3 PE=1 SV=1	Chchd3	7	2	3	2	227	26.3	8.37	66075	ENSMUSG0	mmu:6607			931717.6			1229218		5360636	1954916
High	Q9R045	Angiopoietin-related protein 2 OS=Mus musculus OX=10090 GN=Angptl2 PE=2 SV=2	Angptl2	5	2	3	2	493	57.1	7.75	26360	ENSMUSG0	mmu:2636	1655605								
High	P11688	Integrin alpha-5 OS=Mus musculus OX=10090 GN=Itga5 PE=1 SV=3	Itga5	2	2	4	2	1053	115	5.95	16402	ENSMUSG0	E9QN40; m									
High	P61089	Ubiquitin-conjugating enzyme E2 N OS=Mus musculus OX=10090 GN=Ube2n PE=1 SV=1	Ube2n	13	2	6	2	152	17.1	6.57	93765	ENSMUSG0	mmu:9376			941383.8			1378079	2011091	4192003	2185398
High	Q91XU0	ATPase WRNIP1 OS=Mus musculus OX=10090 GN=Wrnip1 PE=1 SV=2	Wrnip1	7	4	10	4	660	71.7	6.18	78903	ENSMUSG0	mmu:7890	1969414	2992393	5093111	661759.4	2716193	2677763	945502.9	5152684	3589935
High	P35486	Pyruvate dehydrogenase E1 component subunit alpha, somatic form, mitochondrial OS=Mus musculus OX=10090 GN=Pdha1 PE=1 SV=1	Pdha1	5	2	2	2	390	43.2	8.19	18597	ENSMUSG0	mmu:1859								3065254	1747204
High	O09106	Histone deacetylase 1 OS=Mus musculus OX=10090 GN=Hdac1 PE=1 SV=1	Hdac1	12	6	16	3	482	55	5.48	433759	ENSMUSG0	mmu:4337	4126033	837628.9	1932708	1795676	1421465	4871051	2071933	5100947	366972.3
High	Q3TW96	UDP-N-acetylhexosamine pyrophosphorylase-like protein 1 OS=Mus musculus OX=10090 GN=Uap1l1 PE=1 SV=1	Uap1l1	6	2	4	2	507	56.6	5.43	227620	ENSMUSG0	A1L3D3; m									
High	P11531	Dystrophin OS=Mus musculus OX=10090 GN=Dmd PE=1 SV=3	Dmd	0	2	3	2	3678	425.6	5.94	13405	ENSMUSG0	A2A9Z0; m						808868.1		3187591	
High	Q99N94	39S ribosomal protein L9, mitochondrial OS=Mus musculus OX=10090 GN=Mrpl9 PE=1 SV=2	Mrpl9	7	2	10	2	265	30.2	10.08	78523	ENSMUSG0	mmu:7852	533190.8	5971602	6019975	1951961	4448183	8220164	3423178	721086.3	1794029
High	Q9EQC5	N-terminal kinase-like protein OS=Mus musculus OX=10090 GN=Scyl1 PE=1 SV=1	Scyl1	2	2	3	2	806	89.1	6.44	78891	ENSMUSG0	mmu:7889	555934.9		1304917	1199793					
High	P61957	Small ubiquitin-related modifier 2 OS=Mus musculus OX=10090 GN=Sumo2 PE=1 SV=1	Sumo2	24	2	6	2	95	10.9	5.5	170930	ENSMUSG0	A2A9X2; m	325304.4	677536.6	1972888	3497437		2679588	1495863		129054.6
High	Q8C0N1	Kinesin-like protein KIF2B OS=Mus musculus OX=10090 GN=Kif2b PE=1 SV=1	Kif2b	3	2	2	1	668	75.4	8.84	73470	ENSMUSG0	mmu:7347									
High	Q9D1H7	Golgi to ER traffic protein 4 homolog OS=Mus musculus OX=10090 GN=Get4 PE=1 SV=2	Get4	6	2	10	2	327	36.5	5.41	67604	ENSMUSG0	mmu:6760	736453.6		1853604	3212964	1346585				
High	Q6GQT9	BOS complex subunit NOMO1 OS=Mus musculus OX=10090 GN=Nomo1 PE=1 SV=1	Nomo1	7	7	9	7	1214	133.3	6.09	211548	ENSMUSG0	mmu:2115	1592970	419121.4	889515.9		338223.7				588995.8
High	Q8CH77	Neuron navigator 1 OS=Mus musculus OX=10090 GN=Nav1 PE=1 SV=2	Nav1	3	3	4	3	1875	202.2	8.06	215690	ENSMUSG0	mmu:2156		731301.9							
High	Q9Z1X4	Interleukin enhancer-binding factor 3 OS=Mus musculus OX=10090 GN=Ilf3 PE=1 SV=2	Ilf3	5	4	10	4	898	96	8.76	16201	ENSMUSG0	mmu:1620	613006.3							2063839	739132.6
High	Q8C7R4	Ubiquitin-like modifier-activating enzyme 6 OS=Mus musculus OX=10090 GN=Uba6 PE=1 SV=1	Uba6	2	2	7	2	1053	117.9	6.11	231380	ENSMUSG0	mmu:2313	193332.7	3812.152	59417.55			278476.7		805829.9	416553.5
High	Q99JR1	Sideroflexin-1 OS=Mus musculus OX=10090 GN=Sfxn1 PE=1 SV=3	Sfxn1	7	2	2	2	322	35.6	9.23	14057	ENSMUSG0	mmu:1405									
High	Q9QXN3	Activating signal cointegrator 1 OS=Mus musculus OX=10090 GN=Trip4 PE=1 SV=2	Trip4	3	2	4	2	581	66.2	7.55	56404	ENSMUSG0	E9QK64; m	1654608	2537684	4046261	3240559	1921449	7564698	2177142	37640108	14986753
High	Q9Z0R6	Intersectin-2 OS=Mus musculus OX=10090 GN=Itsn2 PE=1 SV=2	Itsn2	1	2	2	2	1659	188.8	8.13			Q8C9C3; Q9									
High	Q6PB44	Tyrosine-protein phosphatase non-receptor type 23 OS=Mus musculus OX=10090 GN=Ptpn23 PE=1 SV=2	Ptpn23	2	2	3	2	1692	185.1	6.8	104831	ENSMUSG0	mmu:1048		799671.5	2947295	3272076	1024340	2979303	2008158	2106581	573731
High	Q8BU11	TOX high mobility group box family member 4 OS=Mus musculus OX=10090 GN=Tox4 PE=1 SV=3	Tox4	3	2	2	2	619	65.9	5.01	268741	ENSMUSG0	mmu:2687									
High	P09405	Nucleolin OS=Mus musculus OX=10090 GN=Ncl PE=1 SV=2	Ncl	5	4	10	4	707	76.7	4.75	17975	ENSMUSG0	mmu:1797		701044.9	1986138		1907974	1151968	987866.9		806131.3
High	Q9D7H3	RNA 3'-terminal phosphate cyclase OS=Mus musculus OX=10090 GN=RtcA PE=1 SV=2	RtcA	5	2	2	2	366	39.2	7.9	66368	ENSMUSG0	mmu:6636	650014.1		1671158	2484853	392900.2	2359436		8832145	
High	P46638	Ras-related protein Rab-11B OS=Mus musculus OX=10090 GN=Rab11b PE=1 SV=3	Rab11b	17	4	7	4	218	24.5	5.94	19326	ENSMUSG0	mmu:1932	516315.5								1267571
High	O35841	Apoptosis inhibitor 5 OS=Mus musculus OX=10090 GN=Api5 PE=1 SV=2	Api5	6	2	4	2	504	56.8	5.92	11800	ENSMUSG0	mmu:1180	1617648	1362123	2150539	1980574	1494937	1797825	3569105	6542097	3120177
High	Q6P5F9	Exportin-1 OS=Mus musculus OX=10090 GN=Xpo1 PE=1 SV=1	Xpo1	3	4	10	4	1071	123	6.07	103573	ENSMUSG0	mmu:1035	1783599		2267565			4597398		5000699	2355542
High	Q9DC23	DnaJ homolog subfamily C member 10 OS=Mus musculus OX=10090 GN=Dnajc10 PE=1 SV=2	Dnajc10	5	4	7	4	793	90.5	6.96	66861	ENSMUSG0	A2ASA2; m	15344002	3133336	14770947	2873760	1479648	5379285	6830781	40082926	14932185
High	Q8BTV2	Cleavage and polyadenylation specificity factor subunit 7 OS=Mus musculus OX=10090 GN=Cpsf7 PE=1 SV=2	Cpsf7	5	2	7	2	471	52	8	269061	ENSMUSG0	mmu:2690		744129.4	3638738	1307211	621347.4	894374.5			
High	Q8BP71	RNA binding protein fox-1 homolog 2 OS=Mus musculus OX=10090 GN=Rbfox2 PE=1 SV=2	Rbfox2	7	2	6	2	449	47.3	6.55	93686	ENSMUSG0	mmu:9368		916229.6	1957816						
High	P70279	Surfeit locus protein 6 OS=Mus musculus OX=10090 GN=Surf6 PE=1 SV=1	Surf6	8	3	5	3	355	41.2	10.52	20935	ENSMUSG0	mmu:2093	1199548	1734980	2507534	381410.2	691947.4	1631335	375998	2805946	478773.8

High	Q9DCR2	AP-3 complex subunit sigma-1 OS=Mus musculus OX=10090 GN=Ap3s1 PE=1 SV=2	Ap3s1	15	2	2	2	193	21.7	5.39	11777	ENSMUSG0	mmu:1177									
High	Q99MD9	Nuclear autoantigenic sperm protein OS=Mus musculus OX=10090 GN=Nasp PE=1 SV=2	Nasp	2	2	5	2	773	83.9	4.37	50927	ENSMUSG0	mmu:5092	797040.1	988811.9	2753380	743171.4	1433049	808702.8	727527.1		
High	Q91XI1	tRNA-dihydrouridine(47) synthase [NAD(P)(+)]-like OS=Mus musculus OX=10090 GN=Dus3l PE=1 SV=1	Dus3l	3	2	7	2	637	71	7.97	224907		mmu:2249	1462938	791047.9	2824074	1878915	452939.7	3403881	1492876	3625576	833448.3
High	Q8K1I7	WAS/WASL-interacting protein family member 1 OS=Mus musculus OX=10090 GN=Wipf1 PE=1 SV=1	Wipf1	12	5	14	5	493	50.1	11.41	215280	ENSMUSG0	mmu:2152	261568.9	3640192	3889889	888941.6	3642088				
High	Q6PDG5	SWI/SNF complex subunit SMARCC2 OS=Mus musculus OX=10090 GN=Smarcc2 PE=1 SV=2	Smarcc2	2	2	5	1	1213	132.5	5.59	68094	ENSMUSG0	mmu:6809			536584.2						
High	Q99JX7	Nuclear RNA export factor 1 OS=Mus musculus OX=10090 GN=Nxf1 PE=1 SV=3	Nxf1	3	2	2	2	618	70.3	8.73	53319	ENSMUSG0	mmu:5331	3773194	1392912	1843606	6491158	1043258	7683039		3469438	1250010
High	Q6IR34	G-protein-signaling modulator 1 OS=Mus musculus OX=10090 GN=Gpsm1 PE=1 SV=3	Gpsm1	4	2	2	2	673	74.3	6.23	67839	ENSMUSG0	A2AIX7; A2			1776942		987155.6	1247617			
High	Q8CAF4	NHS-like protein 1 OS=Mus musculus OX=10090 GN=Nhsl1 PE=1 SV=3	Nhsl1	2	3	6	3	1587	169.3	7.49	215819	ENSMUSG0	mmu:2158	448999.3	1019929	927505.3						
High	P97760	DNA-directed RNA polymerase II subunit RPB3 OS=Mus musculus OX=10090 GN=Polr2c PE=1 SV=2	Polr2c	5	2	14	2	275	31.4	4.92	20021	ENSMUSG0	mmu:2002	770898.8	2175507	4080800	1767033		6723546	1524474	4253892	1872785
High	P62715	Serine/threonine-protein phosphatase 2A catalytic subunit beta isoform OS=Mus musculus OX=10090 GN=Ppp2cb PE=1 SV=1	Ppp2cb	11	2	2	2	309	35.6	5.43	19053	ENSMUSG0	mmu:1905									
High	Q6DVA0	LEM domain-containing protein 2 OS=Mus musculus OX=10090 GN=Lemd2 PE=1 SV=1	Lemd2	5	2	6	2	511	57.5	9.01	224640	ENSMUSG0	mmu:2246	318517.8	1893761	2357443	4394797	1551255	4244128	4613806	13971409	2775338
High	Q05512	Serine/threonine-protein kinase MARK2 OS=Mus musculus OX=10090 GN=Mark2 PE=1 SV=3	Mark2	5	3	6	3	776	86.3	9.67	13728		mmu:1372	1594719	1063567	2031543			994563.8		5524845	
High	P61290	Proteasome activator complex subunit 3 OS=Mus musculus OX=10090 GN=Psme3 PE=1 SV=1	Psme3	11	2	4	2	254	29.5	5.95	19192	ENSMUSG0	mmu:1919	503332.9	334009.6	1142756			1372415		2057834	1385543
High	Q6IRU5	Clathrin light chain B OS=Mus musculus OX=10090 GN=Cltb PE=1 SV=1	Cltb	8	2	2	2	229	25.2	4.63	74325	ENSMUSG0	mmu:7432						1554148	941524.9		411195.9
High	P70335	Rho-associated protein kinase 1 OS=Mus musculus OX=10090 GN=Rock1 PE=1 SV=1	Rock1	3	5	7	3	1354	158.1	5.86	19877	ENSMUSG0	mmu:1987									
High	Q80U58	Pumilio homolog 2 OS=Mus musculus OX=10090 GN=Pum2 PE=1 SV=2	Pum2	2	3	7	1	1066	114.2	7.08	80913	ENSMUSG0	mmu:8091									
High	P50543	Protein S100-A11 OS=Mus musculus OX=10090 GN=S100a11 PE=1 SV=1	S100a11	35	4	8	4	98	11.1	5.45	20195	ENSMUSG0	mmu:2019		1791606	2284470	2413596	2941233	1759305	1311099	4566096	2412205
High	Q61024	Asparagine synthetase [glutamine-hydrolyzing] OS=Mus musculus OX=10090 GN=Asns PE=1 SV=3	Asns	3	2	5	2	561	64.2	6.58	27053	ENSMUSG0	mmu:2705	1922435	1231457	2088231	1201250	988358.3	3446707	2655255		
High	Q9JKX6	ADP-sugar pyrophosphatase OS=Mus musculus OX=10090 GN=Nudt5 PE=1 SV=1	Nudt5	10	2	4	2	218	24	5.48	53893	ENSMUSG0	A2ATT6; mr	396755	434743.7	739015.9			2391950		6475715	1059673
High	Q9CX56	26S proteasome non-ATPase regulatory subunit 8 OS=Mus musculus OX=10090 GN=Psmd8 PE=1 SV=2	Psmd8	9	4	9	4	353	39.9	9.58	57296	ENSMUSG0	mmu:5729								3177278	4381006
High	Q9WV06	Ankyrin repeat domain-containing protein 2 OS=Mus musculus OX=10090 GN=Ankrd2 PE=1 SV=3	Ankrd2	10	3	3	3	328	36.7	6.35	56642	ENSMUSG0	B2RS77; mr									
High	Q9Z2B9	Ribosomal protein S6 kinase alpha-4 OS=Mus musculus OX=10090 GN=Rps6ka4 PE=1 SV=2	Rps6ka4	3	2	5	2	773	85.6	8.27	56613	ENSMUSG0	mmu:5661	687496.5	3869710	7286734	5509940		4363564	3770166		1564824
High	Q80TP3	E3 ubiquitin-protein ligase UBR5 OS=Mus musculus OX=10090 GN=Ubr5 PE=1 SV=2	Ubr5	2	3	4	3	2792	308.2	5.85	70790		mmu:7079	343652	1560318	1390431						
High	Q9DBR0	A-kinase anchor protein 8 OS=Mus musculus OX=10090 GN=Akap8 PE=1 SV=1	Akap8	4	3	4	3	687	76.2	5.14	56399	ENSMUSG0	mmu:5639	1094666	693996.8	3027742	1235861	986727.9	2677223			
High	Q61655	ATP-dependent RNA helicase DDX19A OS=Mus musculus OX=10090 GN=Ddx19a PE=1 SV=2	Ddx19a	7	3	4	3	478	53.9	6.67	13680	ENSMUSG0	mmu:1368									
High	Q91YE3	Egl nine homolog 1 OS=Mus musculus OX=10090 GN=Egln1 PE=1 SV=2	Egln1	8	3	11	3	400	43.1	8.29	112405	ENSMUSG0	mmu:1124	389642.4	2646499	4624955	3004209	1581859	4035086	2804160	2490953	995247.5
High	P35569	Insulin receptor substrate 1 OS=Mus musculus OX=10090 GN=Irs1 PE=1 SV=1	Irs1	2	2	3	2	1233	130.6	8.73	16367		mmu:1636									
High	P53569	CCAAT/enhancer-binding protein zeta OS=Mus musculus OX=10090 GN=Cebpz PE=1 SV=2	Cebpz	3	3	5	3	1052	120.2	5.74	12607		mmu:1260	281774.9	525243.2	1355729	2394264	820785.1			1372534	
High	Q3TDD9	Protein phosphatase 1 regulatory subunit 21 OS=Mus musculus OX=10090 GN=Ppp1r21 PE=1 SV=2	Ppp1r21	4	4	7	4	780	88.3	6.9	73825	ENSMUSG0	B2RPZ6; mr	529166.8		828453.6	1386001	462120.9	571624.6		3787324	993546.3

Supplemental Table 3: ATOX1 proximal protein hits in myoblasts, myocytes, and myotubes

Myoblasts

Gene Symbol	Score Sequest HT: Sequest HT	Abundance Ratio: (Myoblasts (MB), PLUS Bis-Phenol (BP)) / (Myoblasts (MB), MINUS Bis Phenol (BP))	Abundance Ratio P-Value: (Myoblasts (MB), PLUS Bis-Phenol (BP)) / (Myoblasts (MB), MINUS Bis Phenol (BP))	Abundance: F3: Sample, Myoblasts (MB), PLUS Bis-Phenol (BP)	Abundance: F5: Sample, Myoblasts (MB), PLUS Bis-Phenol (BP)	Abundance: F6: Sample, Myoblasts (MB), PLUS Bis-Phenol (BP)
Tubb1	636.19	100	1E-17	489580.313	1122725.25	2467769.75
Synpo2l	270.93	100	1E-17	214626.453	1776565.44	1811817.33
Hspa1l	180.79	100	1E-17	4223702.38	9190227.06	20911355.4
Trap1	164.74	100	1E-17	5505893.41	1247811.5	5008754.25
Lpp	163.34	100	1E-17	4211149.14	48971708.7	40941746.4
Eif4a2	162.41	100	1E-17	1939413.88	2729522.75	6607072.5
Ruvbl2	127.6	100	1E-17	4229878.69	14824305.5	23435115.5
Caprin1	108.35	100	1E-17	6605959.28	30306866.3	105529458
Tmpo	107.39	100	1E-17	1415111.38	1090933.75	3772220.25
Cltc	95.23	100	1E-17	10447307.8	3240014.47	7926013.44
Septin11	75.03	100	1E-17	2301171.5	10287485.3	15709633.8
G3bp2	73.52	100	1E-17	1241273.75	13436811.3	17907049.5
Eef1e1	64.47	100	1E-17	1115781.13	1386052.63	4525055.5
Ranbp3	61.5	100	1E-17	337396.25	9573832.75	11684778.8
Fubp1	60.25	100	1E-17	266222.688	1196996.44	4116212
Rnh1	55.73	100	1E-17	923140.125	2793159.06	410767.313
Dync1li1	55.54	100	1E-17	2917456.39	5170171.16	7673219.56
Col3a1	54.69	100	1E-17	12254552.6	12826720.4	18187374.3
Gfap	54.61	100	1E-17	1071877.38	3876138.5	4394676.5
Ndufs1	53.89	100	1E-17	11803850.4	4450499.78	19009079.9
Erp29	52.6	100	1E-17	10004806.9	3353692.53	7404769.38
Picalm	50.5	100	1E-17	3180771.7	4183363.47	10053729.6
Fxr1	47.64	100	1E-17	2471158	7993789.25	10869210.2
Rsl1d1	47.05	100	1E-17	8798221.28	8240467.5	6444460.78
Ipo9	46.15	100	1E-17	6484964.13	688284.625	10604166
Pdlim2	45.74	100	1E-17	162870.391	431513.719	454990.75
Spart	45.02	100	1E-17	960953.313	3712568.63	8613773.38
Mat2a	42.21	100	1E-17	3108784.31	3917922.06	7781673.06
Xrn2	39.47	100	1E-17	1618030.66	2309708.75	9277973.63
Specc1l	39.27	100	1E-17	939243.656	10999713.1	14713072.4
Ogt	39.14	100	1E-17	1827546.13	2347184.38	13844659
Ikbip	39	100	1E-17	1427093.13	452019.75	741862.875
Cops3	37.93	100	1E-17	239797.219	206923.984	829487.438
P4ha1	37.89	100	1E-17	757031.125	173298.5	473997
Col4a2	37.04	100	1E-17	23667557.1	2005196.63	9100169.06
Cttnbp2nl	36.93	100	1E-17	672443.25	8240271.56	14256701
Impdh2	35.23	100	1E-17	2617878.91	2034040.53	9125910.38
Rai14	35.22	100	1E-17	6174198.06	2915510.63	13588335.6
Scamp3	35.13	100	1E-17	395871.313	1786470.88	2664770.13
Eif4g3	33.67	100	1E-17	2174689.44	5450692.3	9939667.31
Ttll12	32.71	100	1E-17	1246749.69	1523635.44	5026932.13
Crip2	31.35	100	1E-17	642040.25	17458925.1	13717252.9
Psmd5	31.29	100	1E-17	1818717.56	2758065.06	6557850.63
Lmnb1	30.99	100	1E-17	675920.313	9461158.25	10336880.8
Gnai2	30.72	100	1E-17	2767732.5	300634.094	1111622.13
Psmc6	30.35	100	1E-17	2479866.78	437624.047	2294817
Map1a	30.31	100	1E-17	1157879.47	4396681.75	2902220.38
Eif3e	30.23	100	1E-17	3732457.81	5406434.5	5564803.38
Map2k4	29.96	100	1E-17	654942.563	3800468.75	4738297.75
Txlna	29.22	100	1E-17	384451.063	5413135.06	5659168.25
Ahcy	29.04	100	1E-17	2819996.38	577343.25	1622499
Ruvbl1	28.85	100	1E-17	2315516.81	7326967.91	12394344.4
Syncrip	28.83	100	1E-17	303074.563	3019111.88	9046357.81
Rin1	27.67	100	1E-17	1369437.53	2400847.97	7525848.25
Tjp2	27.58	100	1E-17	446730.031	2681708.13	9889631.25
Ehd2	25.88	100	1E-17	662986.063	713614.313	535293.375
Timm50	25.85	100	1E-17	2927248.13	911905.078	5533138
Ddx56	25.16	100	1E-17	2048491.38	1389883.25	2463859.75
Ipo4	24.8	100	1E-17	1788520.38	1364982.63	6375306.25
Hnrnpl	24.47	100	1E-17	579722.172	4273521.75	5426414.38
Ncapd2	24.34	100	1E-17	3129727.78	1686983.38	11501180.4
Gcn1	23.91	100	1E-17	1423388.38	2430814.53	5633794.25
Lemd3	23.49	100	1E-17	448711.5	5185806.13	11770606.8
Nisch	23.24	100	1E-17	501324.531	993712	1404768.63
Nefm	23.16	100	1E-17	4296350.5	3451686.5	6001751.5
Hnrnpab	22.79	100	1E-17	714468.063	5541862.88	10481334.1
Mcm6	22.65	100	1E-17	5103119.22	2596636.47	12711049.1
FAM120A	22.52	100	1E-17	2406480.81	2527752.88	7098468
Kif2a	22.45	100	1E-17	2432870.88	1243863.38	5956421.13
Colgalt1	21.77	100	1E-17	3618033	1287331.95	4155687.38
Hspa4l	21.56	100	1E-17	1886566.84	1324707.75	8108556.13
Pls3	21.27	100	1E-17	400387.125	2672235.25	5426833.75
Slain2	21.11	100	1E-17	2314926.5	3752356.5	5703805
Ipo7	20.6	100	1E-17	1086027.88	565085.5	4436098
Atad3	20.55	100	1E-17	848323.875	2789746.38	2832410
Pfkl	20.27	100	1E-17	1362658.63	1301796.25	2579625
Pck2	20.25	100	1E-17	5284120.25	1215712.44	6298318.94
Wiz	20.11	100	1E-17	2467498.75	6396825.38	12549766
Nap1l1	19.87	100	1E-17	10144267.2	2410888.97	6428303.81
Shc1	19.67	100	1E-17	745095.125	2420936.25	3971277.5
Ppfibp1	19.43	100	1E-17	1275726.3	519309.602	3807837.27
Top1	18.77	100	1E-17	1937241.44	1049843.13	3320521.19
Nup50	18.74	100	1E-17	433927.094	1919039.88	3041810.13
Dnm1l	18.68	100	1E-17	1088483.28	2192258.75	6181801.5
Cars1	18.43	100	1E-17	1296328.75	1147305.11	6033562
Wnk1	18.12	100	1E-17	1756897.5	10245914.9	10275981.9
Csde1	18.03	100	1E-17	1837940.78	3179969.72	12891624
Usp8	17.9	100	1E-17	1808834.22	2989946.03	8468531.44
Klc1	17.22	100	1E-17	1203918.88	1849420.38	3185814.5
Bod1l	16.69	100	1E-17	1152306.38	6448271	6874281.13
Mtdh	16.52	100	1E-17	289249.594	8680893.63	7550886.13
Smarcc1	16.04	100	1E-17	1345718.72	1351232.41	7356689.63
Luzp1	15.79	100	1E-17	1436794.66	3214754.44	5991423.56
Sqstm1	15.79	100	1E-17	6942777.44	3509749.41	11655409.8
Cfl2	15.54	100	1E-17	431572.531	211388.328	500354.594
Cap1	15.12	100	1E-17	3854926.63	450143.813	2047222.94
Got1	14.49	100	1E-17	1088964.94	2737335.5	4602134.16
Zc3h11a	14.22	100	1E-17	381220.77	725043.719	7749955.2
Eif4e	14.16	100	1E-17	1504886.28	1455975.88	2273756.25
Ppp1cb	14.14	100	1E-17	1005216.59	665707.188	3658400.56
Pygb	13.89	100	1E-17	2538433.69	349007.844	1090232
Errfi1	13.68	100	1E-17	622004.625	2807196.66	959393.25
Fhl2	13.56	100	1E-17	960335.063	458724.156	1027961.69
Pds5a	12.99	100	1E-17	386076.281	1278633.13	3159028.75
Pfkm	12.94	100	1E-17	1207654.5	262770.813	606271.438
Tnc	12.78	100	1E-17	8280470.63	3336597.28	6696196.94
Zc3h18	12.59	100	1E-17	735624.125	2937451.5	6750973.88
Slc25a11	12.35	100	1E-17	1245756.63	847401.406	1914241.25
Stip1	11.94	100	1E-17	366060.531	1308757	2136996.5
Cnot2	11.84	100	1E-17	627017.063	763435.563	2439610.75
Filip1l	11.75	100	1E-17	264533.094	1745793.28	6165941.75
Csrp1	11.69	100	1E-17	847576.813	1169637.25	1064213.5
Rbbp4	11.19	100	1E-17	1640740.63	1739505.75	2876607
Rps6ka3	11.16	100	1E-17	299491.031	1585422.63	3177099.13
Ergic1	10.87	100	1E-17	2002314.13	291545.844	1428666.56
Otud4	10.8	100	1E-17	1288391.81	1507188.38	5285499.75
Uqcrq	10.7	100	1E-17	639003.5	1307038.66	2427558.63
Mavs	10.68	100	1E-17	726627.875	2567819.25	2913432.75
Smad2	10.62	100	1E-17	437752.969	1703241.88	3179647.88

Myocytes

Gene Symbol	Score Sequest HT: Sequest HT	Abundance Ratio: (Myocytes (MC), PLUS Bis-Phenol (BP)) / (Myocytes (MC), MINUS Bis Phenol (BP))	Abundance Ratio P-Value: (Myocytes (MC), PLUS Bis-Phenol (BP)) / (Myocytes (MC), MINUS Bis Phenol (BP))	Abundance: F10: Sample, Myocytes (MC), PLUS Bis-Phenol (BP)	Abundance: F11: Sample, Myocytes (MC), PLUS Bis-Phenol (BP)	Abundance: F12: Sample, Myocytes (MC), PLUS Bis-Phenol (BP)
Ldb3	72.83	100	1E-17	9515903.25	1461395.63	3715698.5
Atl3	15.05	100	1E-17	2519877.41	348265.188	2474472.88
Dcakd	12.29	100	1E-17	618995.875	560948.125	2354427.47
Ckb	4.68	100	1E-17	4392177.5	978573.688	2470898.25
Atp1a1	2.06	100	1E-17	1714719.13	2038979.47	825158.188
Tubb1	636.19	100	1E-17	1670924.38	1093811.63	3962823.75
Hspa1l	180.79	100	1E-17	7582901.5	799988.5	2965405.75
Caprin1	108.35	100	1E-17	38436617.8	26856372.7	36755261.9
Ranbp3	61.5	100	1E-17	7846772.69	7003727.25	6602668.94
Erp29	52.6	100	1E-17	6682536.5	1734811.84	9855910.13
Spart	45.02	100	1E-17	4953127.38	2876800.39	5011357.63
Septin8	44.23	100	1E-17	5417116.94	7372539.5	5522996.88
Xrn2	39.47	100	1E-17	5889091.44	5896313.06	3064705.56
Ikbip	39	100	1E-17	1923955.13	966136.938	2126113
Mapre1	38.47	100	1E-17	12430335	699394.563	12563349.5
Cops3	37.93	100	1E-17	2051345	370249.656	4233210.13
Col4a2	37.04	100	1E-17	6815323.75	644235.438	6394829.5
Cttnbp2nl	36.93	100	1E-17	4608295	5905669	3814531.75
Scamp3	35.13	100	1E-17	8343834.5	2519945.25	1246751.13
Dbnl	33.16	100	1E-17	5639491.38	3985268.63	1827003.75
Crip2	31.35	100	1E-17	41730959.8	8284662.75	15684032
Map1a	30.31	100	1E-17	2508608.69	1622444.38	2183737.75
Ahcy	29.04	100	1E-17	2647688	831732.5	2438428.75
Syncrip	28.83	100	1E-17	5752528.5	3231235.63	4482187.75
Gps1	25.15	100	1E-17	3557519.88	2541552.75	2026926.38
Gcn1	23.91	100	1E-17	3242224	1238110.47	7197101.88
Kif2a	22.45	100	1E-17	1190774.13	2406026.25	3077495
Stam	20.48	100	1E-17	1807742.63	3185069.38	3489329.88
Nap1l1	19.87	100	1E-17	5110430.25	6509781.53	4974882.5
Shc1	19.67	100	1E-17	3195424.25	1987613.88	2692585.25
Eif4h	18.98	100	1E-17	13647020.5	3406474.25	4360395.81
Dnm1l	18.68	100	1E-17	2923399.25	2266391.25	5133491.38
Snx18	18.5	100	1E-17	3158430.88	1724355.94	1428673.75
Cars1	18.43	100	1E-17	3703975	1063771.88	4905088.88
Aspscr1	17.34	100	1E-17	5785641.5	4443574.88	2299710.25
Ezr	17.31	100	1E-17	1764038.13	1144803.13	1329001.63
Mtdh	16.52	100	1E-17	7211290.38	4736509.56	1507112.5
Irf2bpl	16.39	100	1E-17	2402320.69	665960.563	751524.25
Ilf2	16	100	1E-17	3201950.5	692072.438	3012804.25
Sqstm1	15.79	100	1E-17	2368987	1340274	4117175.75
Mtcl1	13.49	100	1E-17	1135717.88	2123970	2002061.75
Srsf3	12.9	100	1E-17	1959812	309471	1851424
Tnc	12.78	100	1E-17	2371248.44	1483567.25	8637748.5
Stip1	11.94	100	1E-17	2870635.75	1721636.75	2881218.81
Filip1l	11.75	100	1E-17	751635.188	3505574.34	1631122.75
Csrp1	11.69	100	1E-17	7521594.5	1831662.88	2566143.5
Nfkb2	11.57	100	1E-17	2027227.38	925267.125	1518270.38
Atp5pb	11.17	100	1E-17	581807.75	968146.531	2978717.38
Smad2	10.62	100	1E-17	4083688.25	2158593.81	3206057.88
Aak1	9.89	100	1E-17	8266274	5032940.63	2576620
Aimp2	9.49	100	1E-17	1146612.13	436328.844	2895420
Arfgap2	9.46	100	1E-17	2726071	924376.563	1618136.5
Foxk1	9.38	100	1E-17	533925.125	1315814.19	1571458.69
Sap130	8.46	100	1E-17	1750044.63	3728650.25	1424680.38
Thoc1	8.04	100	1E-17	2470563.5	2275836	2197887.5
Supt16h	7.96	100	1E-17	3437826.63	1362503.94	3291388.5
Irf2bp2	7.7	100	1E-17	1843952	1599605.06	1422648.13
Hk2	7.69	100	1E-17	1232712	604705.5	946734.75
Fhod1	7.31	100	1E-17	1213903	3760395.38	1700777.94
Atp5mf	7.31	100	1E-17	2771082	607473.188	2898539.75
Ano6	7.14	100	1E-17	4173917.5	1617164.19	3399067.47
Sec16a	7.01	100	1E-17	2494483.75	790628.688	2523443.25
Col12a1	6.61	100	1E-17	2328300.72	98500.0625	1872264.63
Cobll1	6.31	100	1E-17	4358.1084	616615.766	3876.02124
Ppp1r13l	6.05	100	1E-17	1724207.63	249408.563	817844.5
Zc2hc1a	6.03	100	1E-17	2025565.25	996439.563	1140911.63
Hook3	5.83	100	1E-17	5518109	2383919.13	8286210.75
Rab5b	5.79	100	1E-17	1597923.38	538694.188	1292155.13
Tcerg1	5.75	100	1E-17	1706868.63	616401.688	1145557.5
Pcm1	5.65	100	1E-17	1925131.25	1239943.53	2362406
Sf1	5.3	100	1E-17	1263789.88	2044629.25	1438953.5
Tbc1d5	4.94	100	1E-17	1756825.75	508620.938	3221294
Prkar2b	4.57	100	1E-17	3397884.25	1100928.25	5041737
Abt1	3.72	100	1E-17	1925783.38	784249.063	2211680.75
Ltv1	3.47	100	1E-17	180900.531	892549.625	1309421.63
Dab2ip	3.36	100	1E-17	3657125	1645383.25	1663485.5
Cse1l	3.31	100	1E-17	824909.5	461714.5	3034150.38
Ctbp1	2.41	100	1E-17	2204295.5	350296.219	4700696.63
Nudt21	2.08	100	1E-17	2644775.5	1395768.25	3403361.25
Smg9	1.97	100	1E-17	5616261.5	1869260.25	3877687.5
Nasp	1.72	100	1E-17	743171.438	1433048.5	808702.813
Prdx4	177.43	100	1E-17	14842105.1	5651021.5	29321407.5
Anxa3	8.12	100	1E-17	1487566.5	356295.5	1325535.5
Ganab	73.23	100	1E-17	15664627.3	4580945.38	19941326.4
Samd4a	23.94	100	1E-17	639234.688	1447131.38	610689
Myof	18.39	100	1E-17	10704098.8	3007845	7747942.63
Ywhah	70.32	100	1E-17	18806169	9984165.25	11574055.5
Itga3	11.03	100	1E-17	9482983.75	1579655.69	2557100
Ciapin1	7.39	100	1E-17	805017.875	548299.125	2576928
Stk39	2.04	100	1E-17	1667016.88	1192190.25	1667525.63
Immt	74.55	100	1E-17	755395.438	365408.313	838010.813
Tfrc	16.66	100	1E-17	10508683.4	2789798	8621412.19
Farp1	23.83	100	1E-17	6750538.88	2315543.56	4986230.5
Eif3l	16.18	100	1E-17	3239210.75	1093648.88	2501186
G6pdx	21.96	100	1E-17	2022939	2518673.44	6369506.5
Plod2	64.56	100	1E-17	4516341	512649.719	13809353.3
Gmps	7.39	100	1E-17	1697782.63	452834.781	3932613.88
Sart3	7.07	100	1E-17	2910092.25	1783258.94	4565015.13
Sbds	8.61	100	1E-17	113203.297	79983.0703	2590347.31

Myotubes

Gene Symbol	Score Sequest HT: Sequest HT	Abundance Ratio: (Myotubes (MT), PLUS Bis-Phenol (BP)) / (Myotubes (MT), MINUS Bis Phenol (BP))	Abundance Ratio P-Value: (Myotubes (MT), PLUS Bis-Phenol (BP)) / (Myotubes (MT), MINUS Bis Phenol (BP))	Abundance: F16: Sample, Myotubes (MT), PLUS Bis-Phenol (BP)	Abundance: F17: Sample, Myotubes (MT), PLUS Bis-Phenol (BP)	Abundance: F19: Sample, Myotubes (MT), PLUS Bis-Phenol (BP)
P4ha1	37.89	100	1E-17	1231400.88	2526904	1241524.63
Gnb2	7.77	100	1E-17	1199153.88	3790818.75	2358167.75
Atp5mg	3.59	100	1E-17	1573123.25	7848328.75	3586880
Erp29	52.6	100	1E-17	5089276	16611915.8	4072172.81
Ikbip	39	100	1E-17	4389893.5	7393576.75	1657285.34
Mapre1	38.47	100	1E-17	12233217	8326532	2393523.63
Map1a	30.31	100	1E-17	2640289.38	3602011.75	316791.219
Syncrip	28.83	100	1E-17	8959507.25	8904760.88	3517209.69
Tjp2	27.58	100	1E-17	1500427.13	4014758.63	328297.625
Gps1	25.15	100	1E-17	5237647.75	5938706	3389766.75
Stam	20.48	100	1E-17	5512146.5	5748502.25	1303533.5
Nap1l1	19.87	100	1E-17	699192.188	7457822.13	2690214.13
Dnm1l	18.68	100	1E-17	2077023.38	11938351.9	2880349.41
Gnb1	17.33	100	1E-17	880018.438	3196717.75	2141788.75
Smarcc1	16.04	100	1E-17	903261.688	4112524.5	341297.188
Ilf2	16	100	1E-17	1247542.75	4803752.88	1561632.5
Sqstm1	15.79	100	1E-17	2990573.5	12307825	7479927.38
Pygb	13.89	100	1E-17	912038.438	5454385.75	1267226.25
Srsf3	12.9	100	1E-17	1645586.5	2357313.25	1222492.13
Filip1l	11.75	100	1E-17	1029554.38	3509252.13	902976.5
Atp5pb	11.17	100	1E-17	224125.734	10444750.9	5751471.88
Tars1	10.01	100	1E-17	2465714.88	3452776.5	4792420.13
Aimp2	9.49	100	1E-17	1033076.88	4055836	1174716.5
Hectd1	9.14	100	1E-17	219032.953	3919031.56	247382.578
Vps4b	8.79	100	1E-17	823835.25	3640607	1263788.25
Hk2	7.69	100	1E-17	2217535	1357671.75	1096177.25
Kpna6	7.51	100	1E-17	895552.5	4226474	1076590.38
Blvra	6.54	100	1E-17	801513.875	1160622.13	866194.313
Cobll1	6.31	100	1E-17	16627.9316	1318993.63	663682.25
Ppp1r13l	6.05	100	1E-17	901377.75	1778335.5	1202986.13
Sae1	5.64	100	1E-17	1664460	1953679.5	593270.375
Cops4	4.76	100	1E-17	862492.563	9913829.25	3080501.63
Nup35	3.88	100	1E-17	1016604.75	1535285.63	685138.438
Tpr	2.34	100	1E-17	277285.063	467659.281	302999
Nudt21	2.08	100	1E-17	2782096.25	7429444.25	2111013.94
Ube2n	1.85	100	1E-17	2011091	4192003	2185398
Mrtfa	16.37	100	1E-17	2504695.75	2119783.25	850221.5
Stk39	2.04	100	1E-17	1286020.88	1759339.38	701085.313
Casp3	2.05	100	1E-17	1338336	1398908	1101518.75
Farp1	23.83	100	1E-17	3456159.25	2355272.5	1363659
Eif3l	16.18	100	1E-17	3217023.75	2823036	1452424.63
G6pdx	21.96	100	1E-17	1147914	4201858.38	3318901.44
Plod2	64.56	100	1E-17	4404642.88	28227455.8	9381388.13
Gmps	7.39	100	1E-17	2602840	12778776.4	3608123.5
Khdrbs1	132.35	100	1E-17	46094596	13640667.1	3338299.29
Lgals3	47.57	100	1E-17	17574787	19725645.8	5854612
Cpsf6	20.55	100	1E-17	7367839.78	813074.25	2033886.73
G3bp2	73.52	100	1E-17	9024963.88	2701439.5	2036814.5
Hnrnpl	24.47	100	1E-17	7001679	1881744	348051.031
Ranbp1	3.94	100	1E-17	2535056	11292648	2767583.63
Capg	47.43	100	1E-17	9105168.25	3890363.25	972720.25
Ncln	16.35	100	1E-17	3182782	5384203	868403.875
Ruvbl1	28.85	100	1E-17	10894898.6	15034064.8	5995801.72
Adsl	13.06	100	1E-17	6165369.5	18000650.8	3242472.13
Mthfd1l	55.13	100	1E-17	2672604.81	6653260.38	5988658.06
FAM120A	22.52	100	1E-17	5423738.63	4319077	4459691.25
Ptpn23	1.77	100	1E-17	2008157.5	2106581.25	573730.984
Jup	7.87	100	1E-17	4544343.5	10915887.5	3934466.44
Heatr3	14.42	100	1E-17	2177142.25	3385576.25	1446561.25
Trip12	48.89	100	1E-17	2408861	4350976.38	923702.031
Ilk	8.72	100	1E-17	1108087.5	11385611	4765802.5
Kif1c	3.5	100	1E-17	1427876.25	4568231.25	1495583.5
Picalm	50.5	100	1E-17	4224097.88	6075103.5	1890809.13
Egln1	1.61	100	1E-17	2804159.77	2490952.75	995247.5
Klc2	7.69	100	1E-17	5779990.13	9578511.13	5942732.25
Rin1	27.67	100	1E-17	5053687.38	10009826.8	3647917.53
Slc25a12	16.49	100	1E-17	7539488.25	15203805.5	4081047.19
Mcm6	22.65	100	1E-17	4768826.5	8047487.5	279624.188
Rcn1	16.46	100	1E-17	2228770.44	2085255.25	1712754.63
Rps5	3.96	100	1E-17	1150814.75	5386698.88	5502965.44
Eif4e	14.16	100	1E-17	1615432.13	15833313.8	5554598.75
Hdac1	1.83	100	1E-17	2071933.13	5100947	366972.344
Csnk1e	13.02	100	1E-17	2599196.88	3102219.63	902800.438
Rtn4	3.65	100	1E-17	6600208.25	4248010.25	697866.313
Ddx42	22.44	100	1E-17	2739854.38	6210988.5	366724.656
Asph	4.63	100	1E-17	1439726.63	5880311	381007.563

Mtatp8	10.43	100	1E-17	677222.563	1701413.25	2007111
Pycr3	10.4	100	1E-17	644043.375	2549783	4345677.19
Znf326	9.97	100	1E-17	2382261.56	778628.5	1818748.13
Acat1	9.93	100	1E-17	644081.75	639091.875	1776169.25
Polr2a	9.69	100	1E-17	775075.25	2661566.19	4415631.5
Map2k2	9.52	100	1E-17	829817.125	1770815.88	1709259.13
Aimp2	9.49	100	1E-17	1272682.75	408531.281	1262198.75
Hip1r	9.43	100	1E-17	1412611.97	566060.453	5237200.25
Foxk1	9.38	100	1E-17	377268.563	960265.375	4273817.63
Ythdf1	9.34	100	1E-17	959782.25	3588072.25	3441568.63
Hectd1	9.14	100	1E-17	508239	3553796.94	3250877.11
Smarca4	9.08	100	1E-17	1791033.19	1797121.63	3699478.75
Ilk	8.72	100	1E-17	2152782.88	782260.813	3574019.38
Pgam5	8.72	100	1E-17	2484943.94	1058862.5	2505389.19
Pakap	8.69	100	1E-17	767283.938	3974591.25	7299248.66
Cdh2	8.69	100	1E-17	1148070.5	893235.188	2935259.22
Arhgef40	8.33	100	1E-17	415568.094	3764600.38	5759667.88
Pum3	8.24	100	1E-17	1104761.38	755482.313	3152857.13
Ppm1g	7.97	100	1E-17	2593194.95	2226573.53	5520722.39
Supt16h	7.96	100	1E-17	9856703.16	4172989.88	16724613.5
Znf516	7.68	100	1E-17	955653.438	838772.438	1687557.16
Nars1	7.65	100	1E-17	3628398.25	973221.625	3065513.81
Srprb	7.6	100	1E-17	611166.063	1289948.88	1780431.13
Hspa4	7.56	100	1E-17	256882.641	864037.125	728024.563
Dnaaf5	7.54	100	1E-17	1469209.88	880760.234	6422181.06
Kpna1	7.51	100	1E-17	1666742	10030249.2	11782500.3
Fbln2	7.46	100	1E-17	913845.75	758140	1046790.25
Epb41l3	7.44	100	1E-17	414724.281	326055.094	2683465
Snx9	7.4	100	1E-17	998213.25	681184.188	1688528
Rps28	7.35	100	1E-17	3294824.25	2377498.44	9809756.25
Gfpt1	7.35	100	1E-17	1394065.25	412425.688	2460928.38
Fhod1	7.31	100	1E-17	377924.25	1017501.78	2503943.94
Atp5mf	7.31	100	1E-17	343074.031	642197.625	966084.875
Mta2	7.28	100	1E-17	2386939.25	1391427.31	4245662.69
Ddost	7.14	100	1E-17	1022109.13	489259.656	1009995.31
Ano6	7.14	100	1E-17	1700809.44	1670750.63	2529745.56
Sec16a	7.01	100	1E-17	1796214.13	2911364.44	2559017.25
Nup93	6.4	100	1E-17	744927.188	1381977	4966776.75
Psmd6	6.16	100	1E-17	1125795.75	311773.563	1165829.63
Irf2bp1	5.71	100	1E-17	203911.25	1910543.63	3513536.25
Pcm1	5.65	100	1E-17	528941.75	3960237.81	2696505
Map2k1	5.62	100	1E-17	2375467.75	1629340.69	4476695.75
Kif2c	5.61	100	1E-17	196302.391	274463.375	2974290.5
Sec63	5.45	100	1E-17	1112590.44	235911.219	945039.688
Psmd1	5.29	100	1E-17	588898.688	316758.188	3081349.5
Cdc5l	5.25	100	1E-17	1180215.91	864188.516	7549170.94
Dlgap5	5.17	100	1E-17	3387067	6574552.5	11428817.6
Map3k7	4.84	100	1E-17	866528.438	2455022.25	847733.063
Prkar2b	4.57	100	1E-17	22442288	2891760.5	4980339.5
Pum1	4.51	100	1E-17	1916367.69	1143596.75	9186109.88
Tjap1	4.42	100	1E-17	3260070	2176617.25	3268896.75
Copa	4.41	100	1E-17	2140453.56	422338.156	1569638.09
Thoc2	4.31	100	1E-17	290402.156	252711.938	1101172.66
Dst	4.25	100	1E-17	1104471.5	2199578.25	7080922.5
Eif5b	4.04	100	1E-17	1375424.63	1503105.13	5070222
Ranbp1	3.94	100	1E-17	2088754.81	3536688.19	10010924.4
Znf622	3.89	100	1E-17	455166.563	496547.531	1539256.44
Nup35	3.88	100	1E-17	566469.125	1198005.13	4102031.88
Dnaja1	3.82	100	1E-17	550569.75	795494.688	2316701.75
Orc2	3.79	100	1E-17	331159.594	931169.313	2400725.25
Ddx24	3.79	100	1E-17	2023777.28	1279412.38	4612104.5
Abt1	3.72	100	1E-17	660268.688	1460050.63	3053913.75
Atp6v1h	3.69	100	1E-17	2417116.5	1503105.13	6169709.25
Prkaca	3.68	100	1E-17	1164231.5	393236.438	1766558
Rtn4	3.65	100	1E-17	929136.906	1875982.69	1282735.25
Rpf2	3.56	100	1E-17	873599.438	204790.469	890327.625
Tbrg1	3.5	100	1E-17	242237.563	940467.625	747158.188
Stt3b	3.5	100	1E-17	1854448.25	1002354.19	1567668.5
Ltv1	3.47	100	1E-17	410546.531	1176182.38	2274105.25
Suclg2	3.45	100	1E-17	1896471.06	9499805.38	6070667.38
Pcbp4	3.39	100	1E-17	1494033.63	724051	1597797.13
Gna11	3.37	100	1E-17	2158267.25	350615.219	1706046.25
Drg2	3.28	100	1E-17	641588.75	220106.516	847818
Hsd17b12	3.28	100	1E-17	2088470.88	560999.875	1061975.75
Nob1	2.9	100	1E-17	1061823.19	246129.688	1379421.47
Rprd2	2.77	100	1E-17	224207.109	718388.063	353615.031
Tab2	2.27	100	1E-17	302406.125	557298.625	1820807.13
Chaf1b	2.22	100	1E-17	707024.938	1261112.88	1139346.88
Nudt21	2.08	100	1E-17	906920.391	2658996.5	4808933.63
Acot9	2.07	100	1E-17	1535698	185783.219	619596.063
Fblim1	2.07	100	1E-17	341062.688	1045911.13	1033917.5
Eif3m	2.04	100	1E-17	238612.813	114494.93	1298461.75
Isg20l2	1.97	100	1E-17	220695.328	422063.273	340291.063
Nomo1	1.8	100	1E-17	1592970.31	419121.438	889515.875
Uba6	1.79	100	1E-17	193332.656	3812.1521	59417.5469
Ctsz	1.79	100	1E-17	2469653.25	947430.563	2614263.5
Nasp	1.72	100	1E-17	797040.125	988811.938	2753380.44
Dus3l	1.7	100	1E-17	1462937.88	791047.875	2824074
Nhsl1	1.68	100	1E-17	448999.281	1019928.88	927505.313
Mark2	1.66	100	1E-17	1594718.75	1063566.63	2031542.63
Psme3	1.66	100	1E-17	503332.875	334009.625	1142756.38
Asns	1.64	100	1E-17	1922434.56	1231456.94	2088230.66
Cebpz	1.61	100	1E-17	281774.875	525243.188	1355729.13

Supplemental Table 4: Comparison of ATOX1 proximal proteins to ATP7A binding partners

Myoblasts overlap

Comstra et al.	Rutaraj et al.	Both
EIF4A2	Trap1	Eif4a2
RUVBL2	Lpp	Ruvbl2
CAPRIN1	Eif4a2	Caprin1
G3BP2	Ruvbl2	Fxr1
FXR1	Caprin1	Crip2
IPO9	Cltc	Ahcy
OGT	Rnh1	Syncrip
CRIP2	Fxr1	Csde1
EIF3E	Mat2a	Ppp1cb
TXLNA	Impdh2	Pgam5
AHCY	Scamp3	Hspa4
RUVBL1	Ttll12	Copa
SYNCRIP	Crip2	Cltc
FAM120A	Psmd5	Rnh1
KIF2A	Gnai2	Scamp3
IPO7	Ahcy	Dnm1l
NAP1L1	Syncrip	Pygb
SHC1	Tjp2	Snx9
CSDE1	Hnrnpl	Eif5b
SMARCC1	Hnrnpab	Asns
SQSTM1	Pls3	
EIF4E	Pfkl	
PPP1CB	Top1	
PFKM	Dnm1l	
STIP1	Csde1	
CNOT2	Cap1	
RBBP4	Zc3h11a	
AIMP2	Ppp1cb	
SMARCA4	Pygb	
PGAM5	Zc3h18	
CDH2	Csrp1	
PPM1G	Mavs	
HSPA4	Smad2	
KPNA1	Acat1	
SEC16A	Pgam5	
PSMD1	Supt16h	
PRKAR2B	Nars1	
PUM1	Hspa4	
COPA	Snx9	
RANBP1	Gfpt1	
DNAJA1	Map2k1	
EIF3M	Copa	
CLTC	Eif5b	
EEF1E1	Rtn4	
RNH1	Gna11	
DYNC1LI1	Hsd17b12	
SCAMP3	Asns	
EIF4G3		
PSMC6		
DNM1L		
PYGB		
OTUD4		
SNX9		
RPS28		
PSMD6		
SEC63		
EIF5B		
DDX24		
ATP6V1H		
ASNS		

Myocytes overlap

Comstra et al.	Rutaraj et al.	Both
CAPRIN1	ATP1A1	CAPRIN1
MAPRE1	CAPRIN1	CRIP2
CRIP2	SCAMP3	AHCY
AHCY	DBNL	SYNCRIP
SYNCRIP	CRIP2	EZR
KIF2A	AHCY	YWHAH
NAP1L1	SYNCRIP	EIF3L
SHC1	EIF4H	SCAMP3
EZR	DNM1L	DBNL
SQSTM1	EZR	EIF4H
STIP1	ILF2	DNM1L
AIMP2	SRSF3	
IRF2BP2	CSRP1	
SEC16A	SMAD2	
TBC1D5	ARFGAP2	
PRKAR2B	SUPT16H	
CTBP1	RAB5B	
SMG9	YWHAH	
SAMD4A	ITGA3	
YWHAH	EIF3L	
IMMT		
EIF3L		
SCAMP3		
DBNL		
EIF4H		
DNM1L		
ZC2HC1A		
TCERG1		
CSE1L		
PRDX4		
FARP1		
GMPS		

Myotubes overlap

Comstra et al.	Rutaraj et al.	Both
GNB2	MAPRE1	SYNCRIP
SYNCRIP	SYNCRIP	EIF3L
TJP2	NAP1L1	KHDRBS1
DNM1L	SMARCC1	DNM1L
GNB1	SQSTM1	PYGB
ILF2	AIMP2	
PYGB	KPNA6	
SRSF3	TPR	
EIF3L	EIF3L	
KHDRBS1	KHDRBS1	
LGALS3	G3BP2	
HNRNPL	RANBP1	
CAPG	RUVBL1	
JUP	FAM120A	
RTN4	PTPN23	
	EIF4E	
	DNM1L	
	PYGB	
	CASP3	
	FARP1	
	GMPS	
